# Supplementary material for: Association of skirt size and postmenopausal breast cancer risk in older women: a cohort study within the UK Collaborative Trial of Ovarian Cancer Screening (UKCTOCS)
Source: BMJ Open. 2014 Sep 3;4(9):e005400. doi: 10.1136/bmjopen-2014-005400 (PMC4185339; doi:10.1136/bmjopen-2014-005400)
Supplement: Author's manuscript [file bmjopen-2014-005400.draft_revisions.pdf]

BMJ Open

Association of skirt size and postmenopausal breast cancer risk in older women: a cohort study within the UK Collaborative Trial of Ovarian Cancer Screening (UKCTOCS)

Journal:	*BMJ Open*
Manuscript ID:	bmjopen-2014-005400
Article Type:	Research
Date Submitted by the Author:	03-Apr-2014

Complete List of Authors:	Fourkala, Evangelia-Ourania; UCL Institute for Women’s Health and NIHR University College London Hospitals Biomedical Research Centre, Department of Women’s Cancer<br>Burnell, Matthew; University College London, Women's Cancer<br>Cox, Catharine; University College London, Women's Cancer<br>Ryan, Andy; University College London, Gynaecological Cancer Research Centre<br>Salter, Laura; University College London Hospitals NHS Foundation Trust,<br>Gentry-Maharaj, Aleksandra; University College London, Women's Cancer<br>Parmar , Mahesh ; University College London, Hub for Trials Methodology<br>Jacobs, Ian; University of Manchester, ; University College London, Gynaecological Cancer Research Centre<br>Menon, Usha; University College London, Gynaecological Oncology

**Primary Subject Heading**:	Oncology
Secondary Subject Heading:	Epidemiology
Keywords:	ONCOLOGY, Breast tumours < ONCOLOGY, Epidemiology < ONCOLOGY

SCHOLARONE™
Manuscripts

For peer review only - http://bmjopen.bmj.com/site/about/guidelines.xhtml

Title: Association of skirt size and postmenopausal breast cancer risk in older women: a cohort study within the UK Collaborative Trial of Ovarian Cancer Screening (UKCTOCS)

Authors: Evangelia-Ourania Fourkala[1]*, Matthew Burnell[1]*, Catherine Cox[1], Andy Ryan[1], Laura Currin Salter[2], Aleksandra Gentry-Maharaj[1], Mahesh Parmar[3], Ian Jacobs[1, 4], Usha Menon[1]**

*joint; ** corresponding author

Affiliations:

[1]Women's Cancer, Institute for Women's Health, University College London, London, UK

[2]University College London Hospitals NHS Foundation Trust, London, UK

[3]MRC Clinical Trials Unit, London, UK

[4]Academic Health Science Centre, University of Manchester, Manchester, UK

Corresponding author: Usha Menon, Gynaecological Cancer Research Centre, Women's Cancer, Institute for Women's Health, University College London, 149 Tottenham Road, London W1T 7DN, UK. u.menon@ucl.ac.uk

For peer review only - http://bmjopen.bmj.com/site/about/guidelines.xhtml

Abstract:

Objectives: Several studies suggest that overall and central-obesity are associated with increased breast cancer (BC) risk in postmenopausal women. However, there are no studies investigating changes of central obesity and BC. We report on the association of BC risk with self-reported skirt size (SS) (waist-circumference proxy) changes between twenties and sixties.

Design: Prospective cohort-study.

Setting: UK Collaborative Trial of Ovarian Cancer Screening (UKCTOCS) involving the nine trial centres in England.

Participants: Postmenopausal-women aged>50 with no known history of breast cancer prior to or on the day of completion of the study-entry questionnaire.

Interventions: At recruitment and at follow-up (current study-entry), women were asked to complete a questionnaire. Women were followed-up via 'flagging' at the NHS Information Centre in England and the Hospital Episode Statistics.

Main outcome-measure: Time to initial breast cancer diagnosis.

Results: Between 2nd January '05 and 1st July '10, 92834 women (median age 64.0) completed the baseline questionnaire. During median follow-up of 3.19 years (25th-75thcentile: 2.46-3.78), 1090 women developed BC. Univariate analysis showed body mass index (BMI) (Hazard Ratio (HR)=1.01, 95% CI: 1.00-1.02, *p*=0.03), SS at baseline (sixties) (HR=1.05; 95% CI: 1.01-1.08, *p*=0.01) and change in SS per 10-years (CSS) (HR=1.38; 95% CI: 1.13-1.69, *p*=0.001) to be associated with BC risk but not SS at twenties (HR=1.01; 95% CI: 0.96-1.06, *p*=0.74). Cox regression model showed the covariate adjusted HR for CSS to be the only significant obesity measure 1.45 (95% CI: 1.22-1.71, *p*=0.001).

For peer review only - http://bmjopen.bmj.com/site/about/guidelines.xhtml

Conclusions: CSS is associated with BC risk independent of BMI. A unit increase in UK SS (e.g.12 to 14) every 10-years between twenties and sixties increases postmenopausal BC risk by 45%. Validation of these results could provide women with a simple and easy to understand message.

Trial registration: International Standard Randomised Controlled Trial Number (ISRCTN)-22488978

For peer review only

Strengths and limitations of this study:

- To the best of our knowledge, this is the first study investigating the association of waist changes using skirt size as a proxy and breast cancer risk.
- We report on a prospective cohort of 94,000 women with comprehensive follow-up through data linkage to multiple national databases showing that between twenties and sixties, an increase in skirt size by one unit every decade increases the risk of postmenopausal breast cancer by 45% while decrease in skirt size is associated with lowering of risk.
- There is a possibility of underestimation of self-reported skirt size, however, if current SS is uniformly underestimated then there is merely a rescaling of CSS so that the strength of the relationship is unaffected. Furthermore, recall bias of the skirt size in the twenties maybe a limitation but and unless this inability is systematically related to future BC, measurement error can only result in underestimating the strength of the true relationship between CSS and BC risk.

For peer review only - http://bmjopen.bmj.com/site/about/guidelines.xhtml

- Given that obesity is now emerging as a global epidemic, from a public health prospective these findings are significant as they provide women with a simple and easy to understand message.

Keywords: breast cancer, risk, skirt size, waist circumference, BMI, postmenopausal

(Manuscript word count: 3 500; No of tables: 5; Number of figures: 2)

For peer review only

For peer review only - http://bmjopen.bmj.com/site/about/guidelines.xhtml

Introduction

Breast cancer(BC) is a major global health concern with 1 384 000 women being diagnosed every year.[1] Significant advances in BC prevention have been accompanied by growing controversies surrounding BC screening. Both highlight the need for better risk stratification. During the last few years a growing number of studies have reported on anthropometric factors, particularly on the relationship between BC risk and overall and/or central obesity.[2-4]

A number of adiposity measures exist. The oldest and most common measure is body mass index(BMI) which is an empirical proxy for human body fat based on an individual's weight and height. Central fat is another measure of obesity. It is usually estimated by waist circumference(WC), waist-hip ratio(WHR), waist-height ratio(WHtR) or the conicity index which evaluates WC in relation to height and weight. An adequate and easy to use proxy measure for WC is clothing (trouser/skirt) size which has been suggested to be independent of BMI.[5] It has also been shown to be a reliable and feasible measure to obtain an estimate of WC at the population level and to predict the percentage of the population at increased health risk.[6]

There is a large body of evidence that high BMI is associated with increased BC risk in postmenopausal women and reduced risk in premenopausal women.[3, 4, 7] Another suggested BC risk predictor is adult weight gain, which typically reflects an increase in body fat.[8-10] Results concerning weight loss are more equivocal; there are studies that show no significant effect upon risk, while some suggest postmenopausal weight loss may be protective.[11-15] Furthermore, the association of central obesity with BC risk is unclear with studies reporting conflicting results.[2, 16] Among postmenopausal women, several studies [17-24] but not all [14, 25-29] have shown

For peer review only - http://bmjopen.bmj.com/site/about/guidelines.xhtml

For peer review only

WC or WHR to be positively associated with increased BC risk.[30] There are, however, no studies examining changes in WC or WHR and BC risk.

The UK Collaborative Trial of Ovarian Cancer Screening(UKCTOCS) [31] with comprehensive follow-up provides the opportunity to examine the association of body size changes with BC risk in a large prospective cohort. Furthermore, the availability of socioeconomic, reproductive, life style and cancer family history data makes it possible to determine whether these factors moderate or potentially affect the association of body changes with BC risk.

For peer review only

For peer review only - http://bmjopen.bmj.com/site/about/guidelines.xhtml

Methods

Setting

UKCTOCS is a multicentre randomised controlled trial of ovarian cancer screening in the general population. The trial invited 1.2 million women of whom 202 638 were recruited between 17thApril 2001 and 27thSeptember 2005 through 13 trial centres in England, Wales and Northern Ireland. The detailed trial design and eligibility criteria have been described elsewhere.[31] All subjects were followed-up through a 'flagging study' with the Health and Social Care National Health Service(NHS) Information Centre providing regular notification of any cancer registrations or deaths in the cohort. In addition, all women were sent a follow-up questionnaire (current study-entry questionnaire) 3-4 years after recruitment. Women provided written consent for use of their data in secondary studies. Ethical approval was obtained from the Joint University College London(UCL)/UCL Hospital Committees on the Ethics of Human Research (REC reference:06/Q0505/102, June 2008).

Subjects

Participants recruited from England who returned the follow-up (current study-entry) questionnaire prior to 2nd July 2010 (censorship date). Women recruited from Northern Ireland and Wales were excluded as Hospital Episode Statistics(HES) was only available for women residing in England.

Data collection

Questionnaires

Recruitment questionnaire: This was completed at recruitment to the trial and included information on variable listed in Table 1A and 1B. This included height and weight which was used to calculate BMI and the postcode which was used to derive the Index of Multiple

For peer review only

Deprivation(IMD). The latter is the official measure of deprivation in England and includes multiple domains such as income, employment, health and disability, education, skills and training, barriers to housing and services, living environment and crime.

Follow-up (current study-entry) questionnaire: At follow-up two specific questions related to skirt size(SS) were asked: (1) What was your SS when you were in your twenties? (2) What is your SS now? Women could choose from 13 SS categories ranging from 6-30. Information on education, lifestyle factors, continuing use of HRT, health status and cancer diagnosis post recruitment as listed in Table 1A and 1B were also collected.

BC notification

For the purpose of this analysis, up-to-date data was provided by the NHS Information Centre on 10th July 2012. HES data was also obtained for all the participants in England for the period between 6th of August 1998 when HES started and 31st of March 2010 when the last upload was obtained. The ICD-9 and ICD-10 codes used to identify women with BC were 174* and C50*, respectively. Women also self-reported BC. Both at recruitment and at current study-entry, women were asked specifically whether they had ever been diagnosed with BC and if so, details of diagnosis date and details of treating physician.[32]

Statistical Analysis

The two SS questions were used to create a variable reflecting change in SS over time. The change in SS variable(CSS) was calculated as an increase in SS per 10 years. Note that a 'one unit' increase in SS means an increase by two nominal values, for example from size 10 to size 12. The date of completion of the study-entry questionnaire was the date used for time of current SS. This was available in 89.8% of the women and when this date was unknown (missing data) then the date of questionnaire entry into the trial management system was

For peer review only - http://bmjopen.bmj.com/site/about/guidelines.xhtml

used. Women were asked to record SS in their twenties, so this time point was approximated by the date of their 25thbirthday. Therefore, women were considered to be 'at risk' from WC changes from the age of 25. Thereafter, until date of study-entry questionnaire, there is 'delayed entry' or 'left-truncation'. This takes into account the fact that a woman's contribution to the analysis is conditional on having survived (from developing BC for the first time) until date of questionnaire. Women with BC previous to study-entry questionnaire completion date were excluded from the analysis. Equating the date for a given age (here, when age=25) with onset of risk means that the analysis is already age-adjusted so that there is no need for age as an additional covariate.

As there can be delays in cancer notification by the National Health and Social Care NHS Information Centre [32], the censorship date was set as 1stJuly 2010, two years prior to the date (10th of July 2012) when data was provided by the NHS Information Centre agencies. This ensured that as far as possible, all BCs in the stated period of observation were identified. Those who died or were lost to follow-up during the study period were censored at that time point.

Summaries of all identified potential confounding risk factors were performed. These variables were also included individually in a Cox regression model to obtain univariable estimates of their hazard ratio relating to BC risk. The CSS was categorised into 4 groups (group 1=decrease in SS per 10 years; group 2=increase of 0 to 0.5 SS per 10 years; group 3=increase of 0.5 to 1 SS per 10 years; group 4=increase of 1+ SS per 10 years) and further summaries of the potential confounders created for each of the 4 groups (supplementary material). Pearson correlation coefficients were calculated for all pairings of the four waist size proxies: SS in their twenties, current SS, CSS and BMI.

For peer review only - http://bmjopen.bmj.com/site/about/guidelines.xhtml

To obtain an appropriate estimate of the relative hazard of BC of increasing SS, a Cox regression model including CSS (uncategorised) and additional risk factors that remained significant at the 5% level following a backward stepwise elimination was fitted. Significance tests were based on robust standard errors that allowed for any residual correlational structure within regional centres. The functional form for CSS was determined by the use of fractional polynomials, taking powers from the restricted set (-2, -1, -0.5, 0, 0.5, 1, 2, 3), to determine the appropriate relationship between CSS and the (log) relative hazard. Cox-Snell residuals were used to test the model fit, and the proportional hazards assumption used in Cox regression was tested using the scaled Schoenfeld residuals for each variable in the final model. The (undue) influence of any outliers was assessed by calculating the change in the parameter estimate when an observation is deleted, known as dfbetas. Predicted relative hazards for a range of CSS values were calculated given the estimated multivariable model, and plotted. To obtain estimates of absolute risk for certain scenarios, survival time was assumed to be exponentially distributed, and the same terms fitted again in an exponential regression model. Survival curves with the same covariate pattern following both the Cox and exponential model fitting were plotted to ensure that the constant hazard was a reasonable assumption when averaged over the timeframe. An estimate of absolute risk was calculated by using the covariate-specific hazard for 1 year and for 5 years.

In the primary analysis, missing data in CSS or any other confounder was handled by case-wise deletion. To assess the robustness of estimation with such an approach to potential biases, multiple imputation was used to calculate the corresponding model estimates with missing data imputed appropriately. Specifically, a multivariate normal distribution formed by all potential predictors was used for the imputed random draws, and 20 fully imputed datasets were created with which to obtain valid estimates and standard errors.

For peer review only - http://bmjopen.bmj.com/site/about/guidelines.xhtml

Results

157 997 women residing in England were recruited to the trial between 17th April 2001 and 29th September 2005. 11 659 (9 440 withdrew, 533 moved away, 1 686 died) women were not sent the study-entry (UKCTOCS follow-up) questionnaire. The remaining 146 338 women were sent the questionnaire between 28th June 2005 and 17th of March 2009 and 112 945 (77.2%) responded by 31st December 2010. 10 689 of these women completed the questionnaire after the censorship date of 1st July 2010 and were therefore excluded.

Eight women did not give consent for flagging. The remaining 102 249 were successfully flagged with the NHS Information Centre. Of these, 9 342 (9.1%) were excluded as they were diagnosed with BC before or on the day of completion of the study-entry questionnaire. In addition, questionnaire completion date could not be calculated in 10 women and 62 were lost to follow-up by the NHS Information Centre prior to questionnaire completion. The remaining 92 834 women entered the study and were followed-up for a median of 3.19 years (25th-75th centile: 2.46-3.78). During the study period, 1 090 (1.2 %) women developed BC.

The reproductive and lifestyle characteristics of the 92 834 women included into the analysis are summarised in Table 1 A and B. Briefly, the women were mainly White, with a median year of birth at 1943 (Interquartile range (IQR): 1937-1947), 19.6% had a University degree and their median IMD (using 2008 data) was 14 (IQR: 9-25). Most women were overweight (median recruitment BMI 25.6, IQR: 23.2-28.8) at trial recruitment (median recruitment age 60.2 (IQR: 55.8-65.8)), had a median SS in the twenties of 12 (IQR: 12-14) and at study-entry of 14 (IQR: 12-16) (median age 64.0 (IQR: 59.7-69.7)). The median CSS was 0.21 (IQR: 0.20-0.43) per 10 years, which is equivalent to an increase of 1 unit in SS (e.g. 10 to

For peer review only - http://bmjopen.bmj.com/site/about/guidelines.xhtml

12) in 50 years. At entry 91 717 number of women (Supplementary Tables S1A and S1B) provided information on the four groups of CSS.

The Pearson's correlation coefficient was r=0.61 between SS at twenties and at current study-entry questionnaire completion, r=0.33 between the SS at twenties and recruitment BMI, r=0.64 between SS at current study-entry questionnaire completion and recruitment BMI and r=0.50 between CSS and recruitment BMI (all p-values<0.0005) (Table 2). As SS at twenties was relatively uncorrelated with CSS (r=-0.15), it was also used as a potential predictor in the Cox model.

Amongst the univariable Cox regressions, all measures of adiposity had significant hazard ratios (HRs) at the 5% level, except for SS at twenties, with CSS having the smallest p-value (p=0.001), compared to SS at current study-entry (p=0.01) and recruitment BMI (p=0.03) (Table 1A and Table 1B). Other significant predictors of BC risk (p<0.05) were age at last period, number pregnancies greater than 6 months, infertility treatment, family history of BC in both mother and sister, number of pregnancies less than 6 months and HRT use (current) at study-entry. Only increases in pregnancies greater than 6 months had a protective effect (Table 1A and Table 1B).

The final Cox model for CSS included 91 368 women, with 1 069 BCs as 1 474 women had missing values for either CSS or one of the included confounders. There was a strong positive association between CSS and BC risk (HR=1.45 per 10 years, 95%CI: 1.22-1.71; p<0.0005). For each unit increase in SS per 10 years the hazard rate of BC increased by 45%. Other predictors significant at the 5% level were age at last period (HR=1.02, 95%CI:1.01-1.04; p<0.0005), infertility (HR=1.39, 95%CI:1.01-1.90; p=0.04), maternal BC history (HR=1.71,

For peer review only - http://bmjopen.bmj.com/site/about/guidelines.xhtml

For peer review only

95%CI:1.57-1.87; $p<0.0005$), number of pregnancies greater than six months (HR=0.91, 95%CI:0.88-0.95; $p<0.0005$) (Table 3). SS at twenties was not retained. There were no interactions with CSS that improved the model. Adding recruitment BMI into the existing model did not improve prediction ($p=0.137$ for recruitment BMI, whilst CSS remained strongly significant, $p=0.001$) (Supplementary Table S2).

Table 4 and Figure 1 show how the model relates BC risk to CSS. For example, for those with an increase of 2 SS every 10 years, the estimated relative hazard more than doubles (adjusted HR 2.21; 95%CI:1.40-2.86). Figure 2 gives the estimated survival functions when CSS=0, CSS=1 and CSS=0.21 (the median CSS value), with all other covariate set at their mean value, and starting at the earliest observed age entering the study. These functions are plotted for both the Cox and exponential model and show that the exponential model is a reasonable approximation over the timeframe considered here. The derived estimates of absolute risk for 1 year were 0.33% (95%CI: 0.30-0.37%) when CSS=0 and 0.48% (95%CI: 0.42-0.54%) when CSS=1 (HR=1.45). These estimates compare with 0.36% (95%CI: 0.33-0.39%) when CSS=0.21 (median CSS) and the raw incidence rate of 0.38% (1090 cancers in 3.07 mean study years). The 5 year absolute risks are 1.66% (95%CI: 1.47-1.84) when CSS=0 and 2.41% (95%CI: 2.11%-2.71%) when CSS=1 and a raw incidence rate of 1.91%. Tests and plots based on the Schoenfeld residuals showed no evidence of any violation of the proportional hazards assumption (plot for CSS – Supplementary Figure S1). The p-values for the test of the PH assumption for each of the five variables ranged from 0.584 to 0.888 indicating the validity of the model. The Cox-Snell residuals plotted against their cumulative hazard showed a virtual line of unity indicating good model fit (Supplementary Figure S2). Use of fractional polynomials of the CSS variable showed the most appropriate functional form was linear, with tests of powers of degrees 1 and 2 giving p-values of over 0.7. The

For peer review only

dfbetas calculated for each observation showed that only for 12 of them did the deletion result in a change of the log-hazard estimate of over 0.01 (approximately equal to a change in the HR of 0.01) with a maximum value of 0.017.

A sensitivity analysis was carried out to investigate the effect of missing data in the independent variables on the model estimation. Data were missing for one or more variables in 1.6% and for CSS in 0.7% of the sample. Twenty completed datasets were created using multiple imputation and the subsequent HR and standard error estimates did not change substantively compared to the original model with case-wise deletion for missing values (Supplementary Table S3).

For peer review only

For peer review only - http://bmjopen.bmj.com/site/about/guidelines.xhtml

Discussion

This is to date the only study that we are aware of investigating the association of change in SS and BC risk. Our data suggests that a unit increase in UK SS (e.g. 12 to 14) every 10 years between the twenties and sixties increases the risk of postmenopausal BC by 45%. In this cohort this translated into an increase in 5 year absolute risk of BC from 1 in 60 to 1 in 41 with each unit increase in SS. SS has been found to be a reliable measure for changes in WC and a measure that women may relate and understand better in comparison to other adiposity measures such as BMI.[5] Use of fractional polynomials of the CSS variable strongly suggest that changes in SS are linearly related to the log hazard rate. This model implies that reductions in SS decrease the risk of BC in the manner plotted in Figure 1, though it should be noted that in almost 75% of the women in this study, SS increased over their adult lifetime.

The major strengths of this study are its prospective cohort design, sample size, comprehensive follow-up through national registries, HES and self-reporting resulting in high quality data and evaluation of numerous possible confounders including life style choices and socioeconomic status. Due to missing data in the covariates, case wise deletion meant 1 474 women were dropped from the Cox regression model. However, a sensitivity analysis using 20 completed sets of multiply imputed data showed that the missing data had little effect on the overall outcome of the study. The absolute risk of BC in this cohort over a median follow-up of 3.2 years was 1.2% as only women in their sixties with no prior history of BC were eligible for this analysis. In keeping with other studies, family history of BC, infertility, age at last period, HRT use were associated with increased BC risk while pregnancies lasting over 6 months were protective.[33] Limitations of the study are the relatively short follow-up period, the possibility of underestimation of self-reported SS and recall bias of the SS in the twenties. However, if current SS is uniformly underestimated across the sample then there is merely a rescaling of the

For peer review only - http://bmjopen.bmj.com/site/about/guidelines.xhtml

CSS variable so that the strength of the relationship is unaffected, although the HR relating to a unit increase would be smaller. The same scenario applies to modern downsizing in SS to reflect increasing BMI (for example. a SS of 12 in their mid-twenties is now equivalent to, say, a SS of 10) assuming that this downsizing trend does not noticeably occur over the period of 'current SS'. All dates of FU questionnaire were between 2005 and 2010 with 97% between 2006 and 2009. For the true relationship to be weaker than reported here would require that underestimation was more prevalent amongst those that did not get BC compared to those that did. Furthermore, unless the inability to accurately recall SS in the twenties is systematically related to future BC or a confounder, measurement error can only result in underestimating the strength of the true relationship between CSS and risk of BC.

The findings that CSS is associated with BC risk (CSS per 10 years HR=1.45, 95%CI: 1.22-1.71; *p*=0.0002) and that addition of BMI to the model does not improve risk prediction, are consistent with previous studies addressing the relationship between body weight and BC risk showing that adult weight gain is a better predictor of BC risk than absolute weight or BMI.[8-10, 30] Increases in WC have also been reported to increase risk of other cancers such as pancreatic [34], endometrial [35] and ovarian [36] cancer. A more equivocal issue in the literature is whether weight loss has a protective effect. Our findings that a decrease in SS is associated with decreased risk are in line with the studies showing reduction of weight can reduce risk of postmenopausal BC.[11, 12]

Absolute SS in the sixties (HR=1.045, 95%CI: 1.011-1.081, p=0.010) but not SS in the twenties was associated with a small increase in BC risk and was shown to be a better predictor compared to BMI. Our findings come in agreement with other studies [30] and in keeping with findings of a comparison of adiposity measures in postmenopausal women where WC was

For peer review only - http://bmjopen.bmj.com/site/about/guidelines.xhtml

found to be a stronger risk factor than BMI for a variety of conditions.[37] Absolute WC has also been associated with increased risk of other cancers such as lung [38] and colon.[39]

In our study changes in SS per 10 years was a better predictor of BC risk compared to absolute SSs. Although the exact mechanism of these relationships need to be better understood, there is a suggestion that body fat around the waist is more metabolically active than adipose tissue elsewhere.[40] Obesity is known to increase oestrogen levels as a result of aromatisation of androstenedione in adipose tissue as well as affect insulin resistance and chronic inflammation, well known factors shown to increase BC risk.[41]

We report for the first time that increase in central adiposity, as indicated by increase in SS per 10 years, is a significant risk factor for BC. Given that obesity is now emerging as a global epidemic, from a public health prospective these findings provide women with a simple and easy to understand message – between twenties and sixties, increase by one SS every 10 years increases the risk of postmenopausal BC by 45% while decrease in SS is associated with lowering of risk. Further validation of these findings is needed.

Conclusion

Convincing evidence is available that high body weight, BMI and adult weight gain, which typically reflect an increase in body fat, are associated with increased breast cancer risk. To the best of our knowledge, this is the first study investigating the association of waist changes using skirt size as a proxy and breast cancer risk. Between twenties and sixties, an increase in skirt size by one unit every decade increases the risk of postmenopausal breast cancer by 45%. Validation of these results could provide women with a simple and easy to understand message.

For peer review only - http://bmjopen.bmj.com/site/about/guidelines.xhtml

Acknowledgements

The authors are particularly grateful to the women throughout the UK who are participating in the trial and to the entire medical, nursing and administrative staff who work on UKCTOCS.

Footnotes

Author's contributions

UM, EOF and MB designed the study. EOF, CC, LCS and UM undertook the literature research. EOF, MB and UM drafted the manuscript. MB and EOF prepared the figures and tables and MB undertook the statistical analysis. EOF, AR, AGM, and UM were involved in data collection. EOF, CC and LCS were involved in data cleaning. All authors contributed to interpretation of the data and critically revised the manuscript and approved the final version.

Funding

UKCTOCS was core funded by the Medical Research Council, Cancer Research UK and National Institute of Health Research (NIHR) with additional support from the Eve Appeal and the NIHR Biomedical Research Centre at UCLH/UCL. The views expressed in the publication are those of the authors and not necessarily those of the funders. The funding source or the sponsor had no role in data collection, data analysis, data interpretation, or writing of the report. The researchers are independent from the funders.

Competing interests

UM and IJ have a financial interest through Abcodia Ltd in the third party exploitation of clinical trials biobanks, which have been developed through the research at UCL. During part of the trial IJ had a consultancy arrangement with Becton Dickinson in the field of tumour markers.

For peer review only - http://bmjopen.bmj.com/site/about/guidelines.xhtml

Patient Consent

Obtained

Ethical approval

The study was approved by the Joint University College London/University College London Hospital Committees on the Ethics of Human Research (REC reference: 06/Q0505/102, June 2008). Approvals for the use of hospital episode statistics data were obtained as part of the standard hospitals episode statistics approval process.

Provenance and peer review

Not commissioned; externally peer reviewed.

Data sharing statement

No additional data are available.

For peer review only

References:

1. http://globocan.iarc.fr/factsheet.asp.

2. Harvie, M., Hooper, L. & Howell, A.H. Central obesity and breast cancer risk: a systematic review. *Obes Rev* 2003;**4**:157-173.

3. Friedenreich, C.M. Review of anthropometric factors and breast cancer risk. *Eur J Cancer Prev* 2001;**10**:15-32.

4. Wiseman, M. The second World Cancer Research Fund/American Institute for Cancer Research expert report. Food, nutrition, physical activity, and the prevention of cancer: a global perspective. *Proc Nutr Soc* 2008;**67**:253-256.

5. Hughes, L.A.E., Schouten, L.J., Goldbohm, R.A. et al. Self-reported Clothing Size as a Proxy Measure for Body Size. *Epidemiology* 2009;**20**:673-676.

6. Battram, D.S., Beynon, C. & He, M. The reliability and validity of using clothing size as a proxy for waist circumference measurement in adults. *Applied physiology, nutrition, and metabolism = Physiologie appliquee, nutrition et metabolisme* 2011;**36**:183-190.

7. Cheraghi, Z., Poorolajal, J., Hashem, T. et al. Effect of body mass index on breast cancer during premenopausal and postmenopausal periods: a meta-analysis. *PLoS One* 2012;**7**:e51446.

8. Eliassen, A.H., Colditz, G.A., Rosner, B. et al. Adult weight change and risk of postmenopausal breast cancer. *Jama* 2006;**296**:193-201.

9. Lahmann, P.H., Schulz, M., Hoffmann, K. et al. Long-term weight change and breast cancer risk: the European prospective investigation into cancer and nutrition (EPIC). *Br J Cancer* 2005;**93**:582-589.

10. Magnusson, C., Baron, J., Persson, I. et al. Body size in different periods of life and breast cancer risk in post-menopausal women. *Int J Cancer* 1998;**76**:29-34.

For peer review only

For peer review only - http://bmjopen.bmj.com/site/about/guidelines.xhtml

11. Eliassen, A.H., Colditz, G.A., Rosner, B. et al. Adult weight change and risk of postmenopausal breast cancer. *JAMA* 2006;**296**:193-201.

12. Parker, E.D. & Folsom, A.R. Intentional weight loss and incidence of obesity-related cancers: the Iowa Women's Health Study. *Int J Obes Relat Metab Disord* 2003;**27**:1447-1452.

13. Radimer, K.L., Ballard-Barbash, R., Miller, J.S. et al. Weight change and the risk of late-onset breast cancer in the original Framingham cohort. *Nutrition and cancer* 2004;**49**:7-13.

14. Morimoto, L.M., White, E., Chen, Z. et al. Obesity, body size, and risk of postmenopausal breast cancer: the Women's Health Initiative (United States). *Cancer Causes Control* 2002;**13**:741-751.

15. Barnes-Josiah, D., Potter, J.D., Sellers, T.A. et al. Early body size and subsequent weight gain as predictors of breast cancer incidence (Iowa, United States). *Cancer Causes Control* 1995;**6**:112-118.

16. Connolly, B.S., Barnett, C., Vogt, K.N. et al. A meta-analysis of published literature on waist-to-hip ratio and risk of breast cancer. *Nutr Cancer* 2002;**44**:127-138.

17. Huang, Z., Willett, W.C., Colditz, G.A. et al. Waist circumference, waist : hip ratio, and risk of breast cancer in the Nurses' Health Study. *Am J Epidemiol* 1999;**150**:1316-1324.

18. Mannisto, S., Pietinen, P., Pyy, M. et al. Body-size indicators and risk of breast cancer according to menopause and estrogen-receptor status. *Int J Cancer* 1996;**68**:8-13.

19. Ogundiran, T.O., Huo, D., Adenipekun, A. et al. Case-control study of body size and breast cancer risk in Nigerian women. *Am J Epidemiol* 2010;**172**:682-690.

20. Wu, A.H., Yu, M.C., Tseng, C.C. et al. Body size, hormone therapy and risk of breast cancer in Asian-American women. *Int J Cancer* 2007;**120**:844-852.

For peer review only

For peer review only - http://bmjopen.bmj.com/site/about/guidelines.xhtml

21. Kaaks, R., Van Noord, P.A., Den Tonkelaar, I. et al. Breast-cancer incidence in relation to height, weight and body-fat distribution in the Dutch "DOM" cohort. *Int J Cancer* 1998;**76**:647-651.

22. Nemesure, B., Wu, S.Y., Hennis, A. et al. Body size and breast cancer in a black population--the Barbados National Cancer Study. *Cancer Causes Control* 2009;**20**:387-394.

23. Folsom, A.R., Kushi, L.H., Anderson, K.E. et al. Associations of general and abdominal obesity with multiple health outcomes in older women: the Iowa Women's Health Study. *Arch Intern Med* 2000;**160**:2117-2128.

24. Fagherazzi, G., Chabbert-Buffet, N., Fabre, A. et al. Hip circumference is associated with the risk of premenopausal ER-/PR- breast cancer. *Int J Obes (Lond)* 2012;**36**:431-439.

25. Muti, P., Stanulla, M., Micheli, A. et al. Markers of insulin resistance and sex steroid hormone activity in relation to breast cancer risk: a prospective analysis of abdominal adiposity, sebum production, and hirsutism (Italy). *Cancer Causes Control* 2000;**11**:721-730.

26. Sonnenschein, E., Toniolo, P., Terry, M.B. et al. Body fat distribution and obesity in pre- and postmenopausal breast cancer. *Int J Epidemiol* 1999;**28**:1026-1031.

27. Lahmann, P.H., Hoffmann, K., Allen, N. et al. Body size and breast cancer risk: findings from the European Prospective Investigation into Cancer And Nutrition (EPIC). *Int J Cancer* 2004;**111**:762-771.

28. den Tonkelaar, I., Seidell, J.C., Collette, H.J. et al. Obesity and subcutaneous fat patterning in relation to breast cancer in postmenopausal women participating in the Diagnostic Investigation of Mammary Cancer Project. *Cancer* 1992;**69**:2663-2667.

For peer review only - http://bmjopen.bmj.com/site/about/guidelines.xhtml

29. Petrek, J.A., Peters, M., Cirrincione, C. et al. Is body fat topography a risk factor for breast cancer? *Ann Intern Med* 1993;**118**:356-362.

30. Pischon, T., Nothlings, U. & Boeing, H. Obesity and cancer. *Proc Nutr Soc* 2008;**67**:128-145.

31. Menon, U., Gentry-Maharaj, A., Ryan, A. et al. Recruitment to multicentre trials--lessons from UKCTOCS: descriptive study. *Bmj* 2008;**337**:a2079.

32. Fourkala, E.O., Gentry-Maharaj, A., Burnell, M. et al. Histological confirmation of breast cancer registration and self-reporting in England and Wales: a cohort study within the UK Collaborative Trial of Ovarian Cancer Screening. *Br J Cancer* 2012;**106**:1910-1916.

33. Veronesi, U., Boyle, P., Goldhirsch, A. et al. Breast cancer. *Lancet* 2005;**365**:1727-1741.

34. Aune, D., Greenwood, D.C., Chan, D.S. et al. Body mass index, abdominal fatness and pancreatic cancer risk: a systematic review and non-linear dose-response meta-analysis of prospective studies. *Ann Oncol* 2012;**23**:843-852.

35. Yang, T.Y., Cairns, B.J., Allen, N. et al. Postmenopausal endometrial cancer risk and body size in early life and middle age: prospective cohort study. *Br J Cancer* 2012;**107**:169-175.

36. Canchola, A.J., Chang, E.T., Bernstein, L. et al. Body size and the risk of ovarian cancer by hormone therapy use in the California Teachers Study cohort. *Cancer Causes Control* 2010;**21**:2241-2248.

37. Hartz, A., He, T. & Rimm, A. Comparison of adiposity measures as risk factors in postmenopausal women. *J Clin Endocrinol Metab* 2012;**97**:227-233.

For peer review only - http://bmjopen.bmj.com/site/about/guidelines.xhtml

38. Kabat, G.C., Kim, M., Hunt, J.R. et al. Body mass index and waist circumference in relation to lung cancer risk in the Women's Health Initiative. *Am J Epidemiol* 2008;**168**:158-169.

39. Moore, L.L., Bradlee, M.L., Singer, M.R. et al. BMI and waist circumference as predictors of lifetime colon cancer risk in Framingham Study adults. *Int J Obes (Lond)* 2004;**28**:559-567.

40. Bjorntorp, P. "Portal" adipose tissue as a generator of risk factors for cardiovascular disease and diabetes. *Arteriosclerosis* 1990;**10**:493-496.

41. Calle, E.E. & Kaaks, R. Overweight, obesity and cancer: epidemiological evidence and proposed mechanisms. *Nat Rev Cancer* 2004;**4**:579-591.

For peer review only

For peer review only - http://bmjopen.bmj.com/site/about/guidelines.xhtml

Table legends

Table 1A. Baseline characteristics and hazard ratios for BC of the identified factors (continuous variables).

Table 1B. Baseline characteristics and hazard ratios for BC of the identified factors (categorical variables).

Table 2. Correlation between skirt size (SS) at twenties, at follow-up (study-entry), change of SS (CSS) per 10 year and body mass index (BMI).

Table 3. Adjusted hazard ratios of BC according to all significant factors.

Table 4. Relative hazards for a range of skirt size (SS) changes per 10 years (CSS) according to estimated multivariable model.

Supplementary tables legends

Supplementary Table S1A. Baseline characteristics of the identified factors (continuous variables) for group 1=decease in skirt size (SS) per 10 years; group 2=increase of 0 to 0.5 SS per 10 years; group 3=increase of 0.5 to 1 SS per 10 years; group 4=increase of 1+ SS per 10 years.

Supplementary Table S1B. Baseline characteristics of the identified factors (categorical variables) for group 1=decease in skirt size (SS) per 10 years; group 2=increase of 0 to 0.5 SS per 10 years; group 3=increase of 0.5 to 1 SS per 10 years; group 4=increase of 1+ SS per 10 years.

For peer review only

For peer review only - http://bmjopen.bmj.com/site/about/guidelines.xhtml

Supplementary Table S2. Hazard ratios for breast cancer (BC) adding body mass index (BMI) into the model.

Supplementary Table S3. Subsequent calculated hazard ratios for breast cancer (BC) by creating twenty completed datasets with multiple imputation.

Figure legends

Figure 1. Relative hazards for a range of skirt size (SS) changes per 10 years (CSS) with 95% confidence bands.

Figure 2. Estimated survival functions for 3 values of change of skirt SS (CSS) (CSS=0, CSS=1, CSS=0.21 (median CSS)) with other covariate values set to their mean value. Solid lines represent estimated curves based on the Cox model, dashed lines represent estimated curves based on the exponential model.

Supplementary figure legends

Supplementary Figure S1. Plot of the scaled Schoenfeld residuals for change of skirt size (CSS) with smoother.

Supplementary Figure S2. Cox-Snell residuals plotted against the estimated cumulative hazard of the change of skirt size (CS) residuals with line of unity for comparison.

For peer review only - http://bmjopen.bmj.com/site/about/guidelines.xhtml

Tables

Table 1A. Baseline characteristics and hazard ratios for BC of the identified factors (continuous variables)

Factor	Mean	SD	p25	p50	p75	Hazard Ratio	Standard Error	Lower 95%CI	Upper 95%CI	p-value
Skirt size at twenty (F)	12.509	2.553	12	12	14	1.008	0.024	0.962	1.056	0.739
Skirt size at study entry (F)	15.134	3.452	12	14	16	1.045	0.018	1.011	1.081	**0.010**
CSS (F)	0.277	0.292	0.202	0.214	0.425	1.382	0.141	1.132	1.688	**0.001**
BMI (R)	26.505	5.073	23.203	25.598	28.821	1.012	0.006	1.001	1.023	**0.029**
IMD (R)	18.745	14.132	9	14	25	1.001	0.002	0.996	1.005	0.778
Age at Last Period (R)	48.975	6.009	46.264	49.977	52.704	1.025	0.006	1.014	1.036	**0.000**
Age at First Period (R)	12.959	1.607	12	13	14	1.02	0.019	0.983	1.059	0.293
No of pregnancies less than 6 months (R)	0.459	0.896	0	0	1	1	0	1	1	0.082
No of pregnancies greater than 6 months (R)	2.02	1.235	2	2	3	1.065	0.033	1.002	1.132	**0.045**
No of cigarettes ever smoked in 100 000s (F)	136.758	251.99	0	0	180	0.912	0.023	0.868	0.096	**0.000**

R variables collected using recruitment questionnaire; F variables collected using follow up questionnaire; BC=BC; BMI=Body mass index; IMD=Index of multiple deprivation ; CI=Confidence interval

For peer review only

Table 1B. Baseline characteristics and hazard ratios for BC of the identified factors (categorical variables)

Factor		All Women N (%)	Hazard Ratio	Standard Error	Lower 95%CI	Upper 95%CI	p-value
Ethnicity (R)	White	90,011 (97.35)	ref				
	Black	832 (0.90)	1.307	0.496	0.622	2.750	0.480
	Asian	490 (0.53)	0.827	0.263	0.443	1.541	0.549
	Chinese	166 (0.18)	0.554	0.554	0.078	3.936	0.555
	Other	669 (0.72)	0.693	0.311	0.288	1.668	0.413
Education (F)	O-Level	35,986 (38.76)	1.208	0.074	1.070	1.363	0.002
	A-Level	14,406 (15.52)	1.204	0.096	1.030	1.407	0.020
	Clerical or commercial qualification	25,767 (27.75)	1.085	0.072	0.953	1.236	0.219
	Nursing or teaching	13,174 (14.19)	1.051	0.090	0.888	1.243	0.565
	Degree college/University	18,202 (19.61)	1.000	0.077	0.859	1.163	0.996
	Other than above qualification	28,752 (30.97)	0.828	0.057	0.724	0.947	0.006
Hysterectomy (R)	No	76,318 (82.20)	ref				
	Yes	16,524 (17.80)	0.961	0.077	0.821	1.125	0.621
Pill Use (R)	No	37,083 (39.94)	ref				
	Yes	55,759 (60.06)	0.971	0.064	0.654	0.852	1.106
HRT Use at recruitment (R)	No	73,544 (79.21)	ref				
	Yes	19,298 (20.79)	1.119	0.081	0.972	1.289	0.118
HRT Use at baseline (F)	No	85,866 (92.50)	ref				
	Yes	6,963 (7.50)	1.232	0.131	1.001	1.517	**0.049**
Sterilisation (R)	No	74,550 (80.30)	ref				
	Yes	18,292 (19.70)	0.952	0.739	0.817	1.108	0.523
Infertility (R)	No	89,646 (96.56)					
	Yes	3,196 (3.44)	1.459	0.208	1.103	1.931	**0.008**
Alcohol (units per week) (F)	None	21,086 (22.97)	ref				
	Less than 1	16,084 (17.52)	1.012	0.099	0.835	1.226	0.905
	1 to 3	18,717 (20.39)	1.063	0.099	0.886	1.275	0.511
	4 to 6	13,813 (15.05)	1.039	0.106	0.851	1.268	0.710
	7 to 10	11,056 (12.04)	0.949	0.106	0.762	1.182	0.640
	11 to 15	6,549 (7.13)	0.985	0.132	0.758	1.280	0.910
	16 to 30	2,990 (3.26)	1.271	0.209	0.920	1.755	0.146

For peer review only

For peer review only - http://bmjopen.bmj.com/site/about/guidelines.xhtml

	21+	1,501 (1.64)	1.331	0.291	0.866	2.044	0.192
Mother BC History (R)	No	86,931 (93.63)	ref				
	Yes	5,911 (6.37)	1.688	0.169	1.387	2.054	**0.000**
Grandmother BC History (R)	0	89,148 (96.02)	ref				
	1	3,621(3.90)	1.158	0.171	0.867	1.547	0.321
	2	73 (0.08)	4.760	2.385	1.783	12.708	0.002
Daughter BC History (R)	0	92,526 (99.66)	ref				
	1	307 (0.33)	0.570	0.404	0.142	2.284	0.427
	2	7 (0.01)	1.87E-12	4.57-E06	0		1.000
	3	1 (0.00)	1.88E-12	0.000	0		1.000
	5	1 (0.00)	1.87E-12	0.000	0		1.000
Sister BC History (R)	0	88,805 (95.65)	ref				
	1	3,821 (4.12)	1.519	0.193	1.183	1.949	**0.001**
	2	207 (0.22)	2.577	1.056	1.155	5.753	**0.021**
	3	7 (0.01)	7.02E-13	2.84E-06	0		1.000
	4	2 (0.00)	7.03E-13	6.53E-06	0		1.000
Mother Ovarian Cancer History (R)	No	91, 394 (98.44)	ref				
	Yes	1,448 (1.56)	0.643	0.195	0.355	1.164	0.145
Grandmother Ovarian Cancer History (R)	0	92,347 (99.47)	ref				
	1	495 (0.53)	1.044	0.427	0.468	2.328	0.917
	2	1 (0.00)	1.03E-10	0.000			1.000
Daughter Ovarian Cancer History (R)	0	92,739 (99.89)	ref				
	1	103 (0.11)	0.787	0.787	0.111	5.593	0.810
Sister Ovarian Cancer History (R)	No	92,023 (99.12)	ref				
	Yes	819 (0.88)	1.148	0.342	0.634	2.081	0.648

R variables collected using recruitment questionnaire; F variables collected using follow up questionnaire; BC= BC; CSS= Change of skirt size per 10 years; HRT= hormone replacement therapy; CI=Confidence interval

For peer review only - http://bmjopen.bmj.com/site/about/guidelines.xhtml

For peer review only

Table 2. Correlation between skirt size at twenties, at study entry (follow-up), change of skirt size (CSS) per 10 year and body mass index (BMI)

	Skirt size at twenties	Skirt size at baseline	BMI	CSS
Skirt size at twenties	1.000			
Skirt size at study entry (follow-up)	0.606 (p<0.0005)	1.000		
BMI	0.330 (p<0.0005)	0.643 (p<0.0005)	1.000	
CSS	-0.1529 (p<0.0005)	0.693 (P<0.0005)	0.497 (p<0.0005)	1.000

BMI=Body mass index; CSS=change of skirt size per 10 years

Table 3. Adjusted hazard ratios of BC according to all significant factors

**Risk factor**	**Hazard Ratio**	**Standard Error**	**Lower 95%CI**	**Upper 95%CI**	**p-value**
Age at last period	1.025	0.005	1.014	1.034	0.000
Infertility	1.389	0.223	1.012	1.905	0.041
Mother BC history	1.711	0.075	1.570	0.948	0.000
Pregnancy greater than 6 months	0.914	0.017	0.882	0.948	0.000
CSS	1.446	0.125	1.220	1.713	0.001

BC=BC; CSS=Change of skirt size per 10 years; CI=Confidence interval

For peer review only - http://bmjopen.bmj.com/site/about/guidelines.xhtml

Table 4. Relative hazards for a range of skirt size changes per 10 years (CSS) according to estimated multivariable model

CSS	Hazard ratio*	Standard error	Lower 95%CI	Upper 95%CI
-1.0	0.686	0.060	0.569	0.803
-0.5	0.828	0.360	0.758	0.898
0.0	1.000			
0.5	1.208	0.525	1.105	1.311
1.0	1.458	0.127	1.209	1.707
1.5	1.761	0.230	1.310	2.211
2.0	2.212	0.370	1.400	2.852

*Relative hazard estimated at mean values for continuous variables and at the baseline group for categorical variables; CSS=Change of skirt size per 10 years

CI=Confidence interval

Figures

For peer review only - http://bmjopen.bmj.com/site/about/guidelines.xhtml

Figure 1. Relative hazards for a range of skirt size changes per 10 years (CSS) with 95% confidence bands.

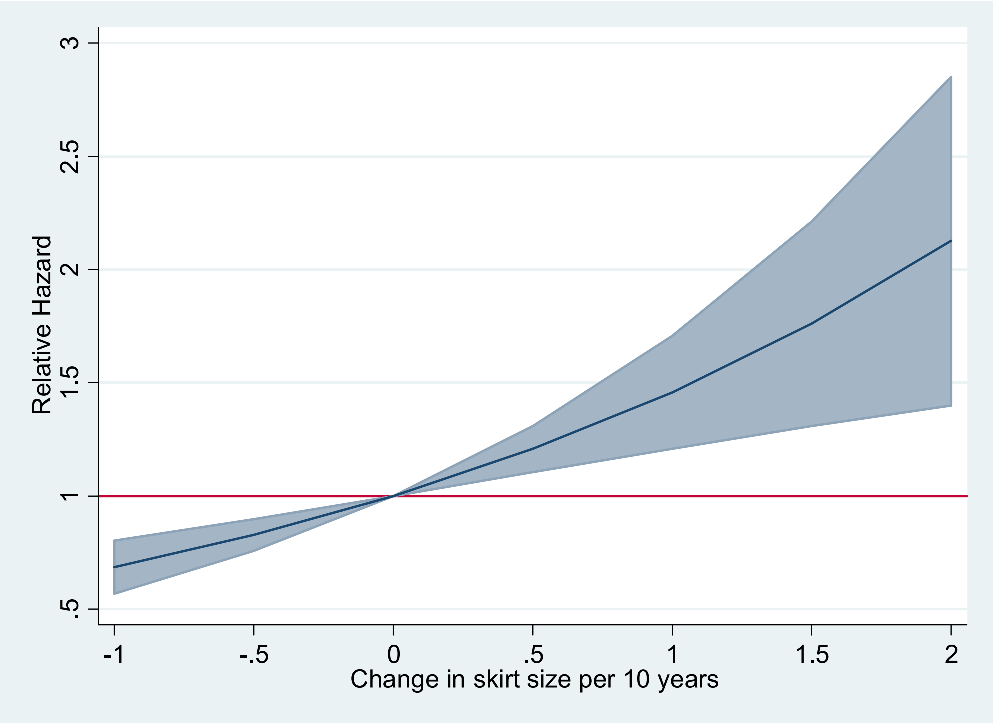

For peer review only - http://bmjopen.bmj.com/site/about/guidelines.xhtml

Figure 2. Estimated survival functions for 3 values of CSS (CSS=0, CSS=1, CSS=0.21 (median CSS)) with other covariate values set to their mean value. Solid lines represent estimated curves based on the Cox model, dashed lines represent estimated curves based on the exponential model.

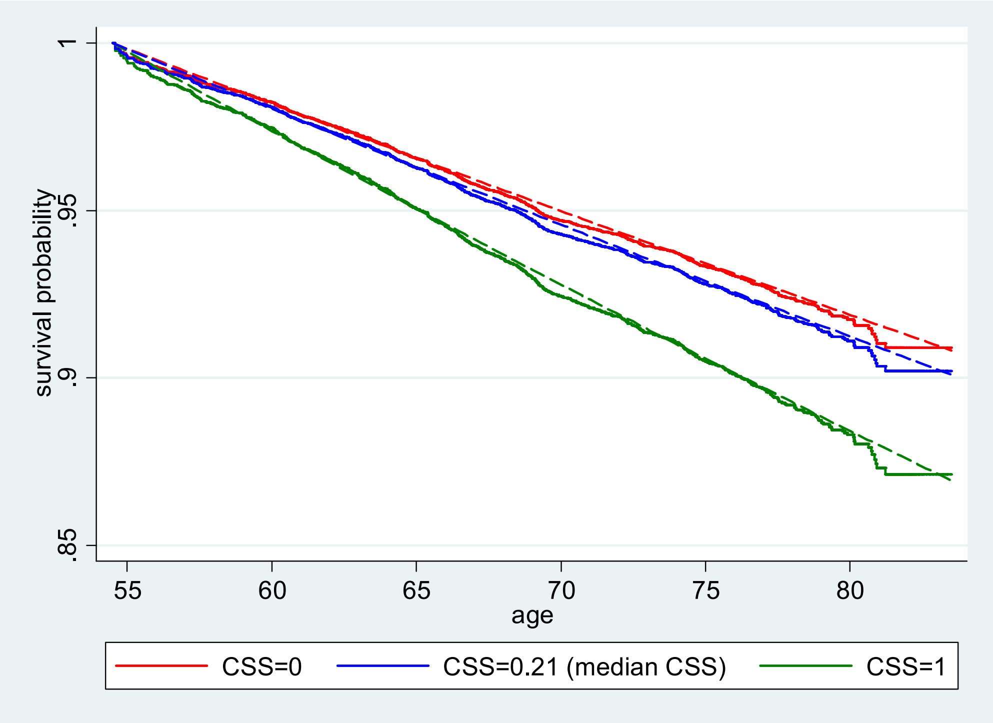

For peer review only - http://bmjopen.bmj.com/site/about/guidelines.xhtml

Supplementary Table S1A. Baseline characteristics of the identified factors (continuous variables) for group 1=decease in skirt size per 10 years; group 2=increase of 0 to 0.5 skirt sizes per 10 years; group 3=increase of 0.5 to 1 skirt sizes per 10 years; group 4=increase of 1+ skirt sizes per 10 years

Factor	Group	Mean	SD	p25	p50	p75
BMI (R)	1	23.618	4.079	21.290	23.030	25.027
	2	26.402	4.269	23.745	25.715	28.340
	3	30.826	5.293	27.344	30.258	33.583
	4	35.303	6.340	31.153	35.073	39.147
	Total	26.496	5.068	23.203	25.598	28.147
IMD (R)	1	17.728	13.440	8	14	24
	2	18.396	13.939	8	14	25
	3	21.149	15.236	10	16	29
	4	24.223	16.490	11	20	34
	Total	18.720	14.118	9	14	25
Age at Last Period (R)	1	49.098	5.841	46.634	50.004	52.646
	2	49.061	5.966	46.360	50.004	52.756
	3	48.490	6.355	45.257	49.802	52.580
	4	48.182	6.482	44.649	49.591	52.660
	Total	48.976	6.005	46.269	49.977	52.703
Age at First Period (R)	1	12.953	1.587	12	13	14
	2	12.981	1.590	12	13	14
	3	12.892	1.678	12	13	14
	4	12.785	1.678	11	13	14
	Total	12.958	1.770	12	13	14
No of pregnancies greater than 6 months (R)	1	0.433	0.868	0	0	1
	2	0.444	0.869	0	0	1
	3	0.539	0.998	0	0	1
	4	0.654	1.166	0	0	1
	Total	0.458	0.895	0	0	1

For peer review only

No of pregnancies less than 6 months (R)	1	1.985	1.193	1	2	3
	2	2.111	1.203	2	2	3
	3	2.275	1.347	2	2	3
	4	2.394	1.509	2	2	3
	Total	2.108	1.231	2	2	3
No of cigarettes ever smoked in 100 000s (F)	1	135.105	246.220	0	0	180
	2	128.294	242.221	0	0	150
	3	168.094	285.889	0	0	240
	4	209.809	328.366	0	10	320
	Total	136.916	251.995	0	0	180

R variables collected using recruitment questionnaire; F variables collected using follow up questionnaire; BMI=Body mass index; IMD=Index of multiple deprivation

For peer review only

Supplementary Table S1B. Baseline characteristics of the identified factors (categorical variables) for group 1=decease in skirt size per 10 years; group 2=increase of 0 to 0.5 skirt sizes per 10 years; group 3=increase of 0.5 to 1 skirt sizes per 10 years; group 4=increase of 1+ skirt sizes per 10 years

Factor		Group 1 N (%)	Group 2 N (%)	Group 3 N (%)	Group 4 N (%)	All Women N (%)
**Ethnicity (R)**	White	21,931 (98.65)	53,636 (97.49)	11,701 (95.650	1,714 (92.90)	88,982 (97.42)
	Black	105 (0.47)	599 (1.09)	275 (2.25)	77 (4.17)	1,056 (1.16)
	Asian	43 (0.19)	296 (0.54)	111(0.91)	32 (1.7)	482 (0.53)
	Chinese	43 (0.19)	102 (0.19)	18 (0.15)	0 (0.00)	163 (0.18)
	Other	109 (0.49)	382 (0.69)	140 (1.14)	22 (1.19)	653 (0.71)
**Education (F)**	O-Level	9,333 (41.79)	21,488 (38.90)	4,284 (34.84)	538 (29.02)	35,643 (38.86)
	A-Level	4,041 (18.10)	8,317 (15.06)	1,677 (13.64)	239 (12.89)	14,274 (15.56)
	Clerical or commercial qualification	6,314 (28.27)	15,648 (28.33)	3,148 (25.60)	423 (22.82)	25,533 (27.84)
	Nursing or teaching	3,377 (15.12)	7,771 (14.07)	1,644 (13.37)	252 (13.59)	13,044 (14.22)
	Degree college/University	5,190 (23.24)	10,502 (19.01)	2,048 (16.66)	296 (15.97)	18,036 (19.66)
	Other than above qualification	6,039 (27.04)	17,032 (30.83)	4,499 (36.59)	787 (42.45)	28,357 (30.92)
**Hysterectomy (R)**	No	18,829 (84.32)	45,329 (82.06)	9,766 (79.42)	1,451 978.26)	75,375 (82.18)
	Yes	3,502 (15.68)	9,907 (17.94)	2,530 (20.58)	403 (21.74)	16,342 (17.82)
**Pill Use (R)**	No	8,422 (37.71)	22,092 (40.00)	5,163 (41.99)	813 (43.85)	36,490 (39.79)
	Yes	13,909 (62.29)	33,144 (60.00)	7,133 (58.01)	1,041 (56.15)	55,227 (60.21)
**HRT Use at recruitment (R)**	No	17,148 (76.79)	43,797 (79.29)	(10,069 (81.89)	1,593 (85.92)	72,607 (79.16)
	Yes	5,183 (23.21)	11,439 (20.71)	2.227 (18.11)	261 (14.08)	19,110 (20.84)
**HRT Use at baseline (F)**	No	20,272 (90.78)	51,183 (92.67)	11,596 (94.31)	1,778 (95.20)	84,829 (92.50)
	Yes	2,058 (9.22)	4,048 (7.33)	699 (5.69)	76 (4.10)	6,881 (7.50)
**Sterilisation (R)**	No	18,154 (81.30)	44,465 (80.50)	9,616 (78.20)	1,391 (75.03)	73,626 (80.28)
	Yes	4,177 (18.70)	10,771 (19.50)	2,680 (21.80)	463 (24.97)	18,091 (19.72)
**Infertility (R)**	No	21,495 (96.26)	53,364 (96.61)	11,903 (96.80)	1,790 (96.55)	88,552 (96.55)
	Yes	836 (3.74)	1,872 (3.39)	393 (3.20)	64 (3.45)	3,165 (3.45)
**Alcohol (units per week)**	None	4,632 (20.95)	11,805 (21.59)	3,510 (28.87)	772 (42.23)	20,719 (22.82)
	Less than 1	3,384 (15.30)	9,612 (17.58)	2,523 (20.75)	377 (20.62)	15,896 (17.51)
	1 to 3	4,343 (19.64)	11,599 (21.21)	2,309 (18.99)	287 (15.70)	18,896 (20.42)
	4 to 6	3,545 (16.03)	8,539 (15.62)	1,444 (11.88)	172 (9.41)	13,700 (15.09)

For peer review only

	7 to 10	3,063 (13.85)	6,694 (12.24)	1,121 (9.22)	102 (5.58)	10,980 (12.10)
	11 to 15	1,926 (13.85)	3,834 (7.01)	670 (5.51)	58 (3.17)	6,488 (7.15)
	16 to 30	803 (3.63)	362 (2.98)	362 (2.98)	33 (1.81)	2,971 (3.27)
	21+	416 (1.88)	219 (1.80)	219 (1.80)	27 (1.48)	1,486 (1.64)
**Mother BC History (R)**	No	20,937 (93.76)	51,711 (93.62)	11,490 (93.45)	1,733 (93.47)	85,871 (93.63)
	Yes	1,394 (6.24)	3,525 (6.38)	806 (6.55)	121 (6.53)	5,846 (6.37)
**Grandmother BC History (R)**	0	21,423 (95.93)	53,025 (96.00)	11,829 (96.20)	1,780 (96.01)	88,057 (96.01)
	1	886 (3.97)	2,179 (3.94)	451 (3.67)	72 (3.88)	3,588 (3.91)
	2	22 (0.10)	32 (0.06)	16 (0.13)	2 (0.11)	72 (0.08)
**Daughter BC History (R)**	0	22,272 (99.74)	55,050 (99.66)	12,238 (99.53)	1,848 (99.68)	91,408 (99.66)
	1	58 (0.26)	180 (0.33)	56 (0.46)	6 (0.32)	300 (0.33)
	2	0 (0.00)	5 (0.01)	2 (0.02)	0 (0.00)	7 (0.01)
	3	1 (0.00)	0 (0.00)	0 (0.00)	0 (0.00)	1 (0.00)
	5	0 (0.00)	1 (0.00)	0 (0.00)	0 (0.00)	1 (0.00)
**Sister BC History (R)**	0	21,443 (96.02)	52,827 (95.64)	11,714 (95.27)	1,753 (94.55)	87,737 (95.66)
	1	852 (3.82)	2,276 (4.12)	546 (4.44)	94 (5.07)	3,768 (4.11)
	2	34 (0.15)	126 (0.23)	36 (0.29)	7 (0.38)	203 (0.22)
	3	2 (0.01)	5 (0.01)	0 (0.00)	0 (0.00)	7 (0.01)
	4	0 (0.00)	2 (0.00)	0 (0.00)	0 (0.00)	2 (0.00)
**Mother Ovarian Cancer History (R)**	No	21,997 (98.50)	54,354 (98.40)	12,099 (98.40)	1,832 (98.81)	90,282 (98.44)
	Yes	334 (1.50)	882 (1.60)	197 (1.60)	22 (1.19)	1,435 (1.56)
		22,226 (99.53)	54,953 (99.49)	12,207 (99.28)	1,842 (99.35)	91,228 (99.47)
**Grandmother Ovarian Cancer History (R)**	0	22,226 (99.53)	54,953 (99.49)	12,207 (99.28)	1,842 (99.35)	91,228 (99.47)
	1	105 (0.47)	282 (0.51)	89 (0.72)	12 (0.65)	488 (0.53)
	2	0 (0.00)	1 (0.00)	0 (0.00)	0 (0.00)	1 (0.00)
**Daughter Ovarian Cancer History (R)**	0	22,309 (99.90)	55,176 (99.89)	12,281 (99.88)	1,848 (99.68)	91,614 (99.89)
	1	22 (0.10)	60 (0.11)	15 (0.12)	6 (0.32)	103 (0.11)
**Sister Ovarian Cancer History (R)**	0	22,145 (99.17)	54,762 (99.14)	12,169 (98.97)	1,835 (98.98)	90,911 (99.12)
	1	186 (0.83)	474 (0.86)	127 (1.03)	19 (1.02)	806 (0.88)

R variables collected using recruitment questionnaire; F variables collected using follow up questionnaire; BC= Breast cancer; HRT= hormone replacement therapy

For peer review only

Supplementary Table S2. Hazard ratios for BC adding body mass index (BMI) into the model

Risk factor	Hazard ratio	Standard error	Lower 95% CI	Upper 95% CI	p-value
Age at last period	1.024	0.005	1.014	1.101	0.000
Infertility	1.394	0.226	1.015	1.015	0.056
Mother BC history	1.721	0.075	1.581	1.581	0.000
Pregnancy greater than 6 months	0.911	0.017	0.877	0.877	0.006
CSS	1.382	0.134	1.142	1.142	0.001
BMI	1.005	0.004	0.998	0.998	0.137

BC=Breast cancer; BMI=Body mass index; CSS=change of skirt size per ten year

Supplementary Table S3. Subsequent calculated hazard ratios for BC by creating twenty completed datasets with multiple imputation

Risk factor	Hazard ratio	Standard error	Lower 95% CI	Upper 95% CI	p-value
Age at last period	1.025	0.005	1.015	1.035	0.000
Infertility	1.362	0.220	0.992	1.870	0.056
Mother BC history	1.675	0.079	1.527	1.839	0.000
Pregnancy greater than 6 months	0.918	0.017	0.884	0.953	0.006
CSS	1.421	0.119	1.207	1.672	0.001

BC=Breast cancer; CSS=change of skirt size per ten year

For peer review only

Supplementary Figure S1. Plot of the scaled Schoenfeld residuals for CSS with smoother.

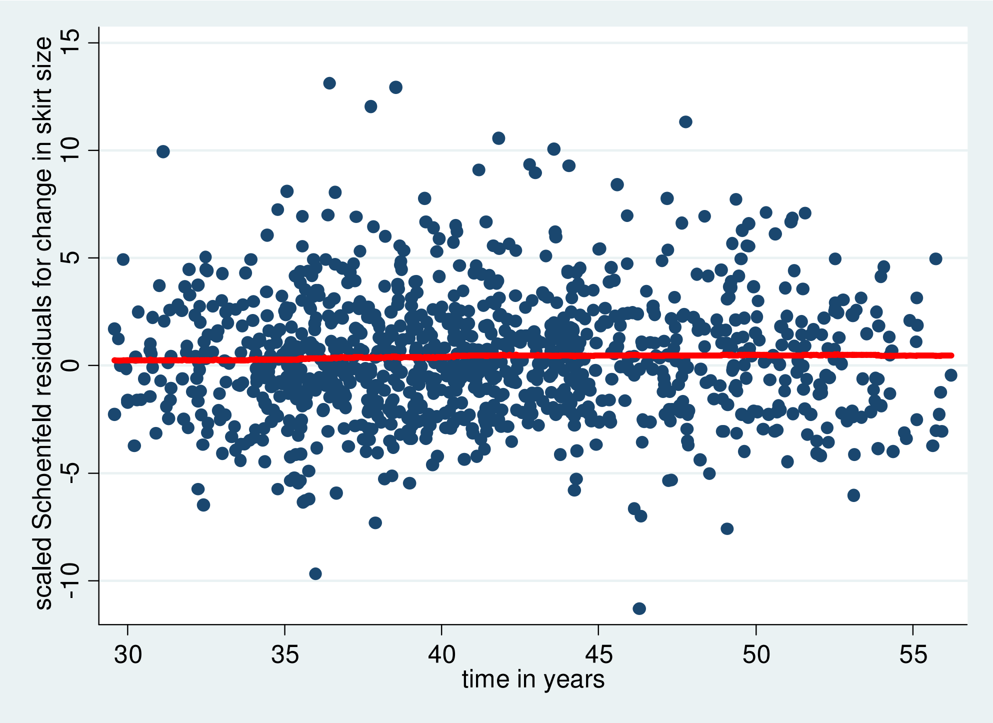

For peer review only - http://bmjopen.bmj.com/site/about/guidelines.xhtml

Supplementary Figure S2. Cox-Snell residuals plotted against the estimated cumulative hazard of the CS residuals with line of unity for comparison.

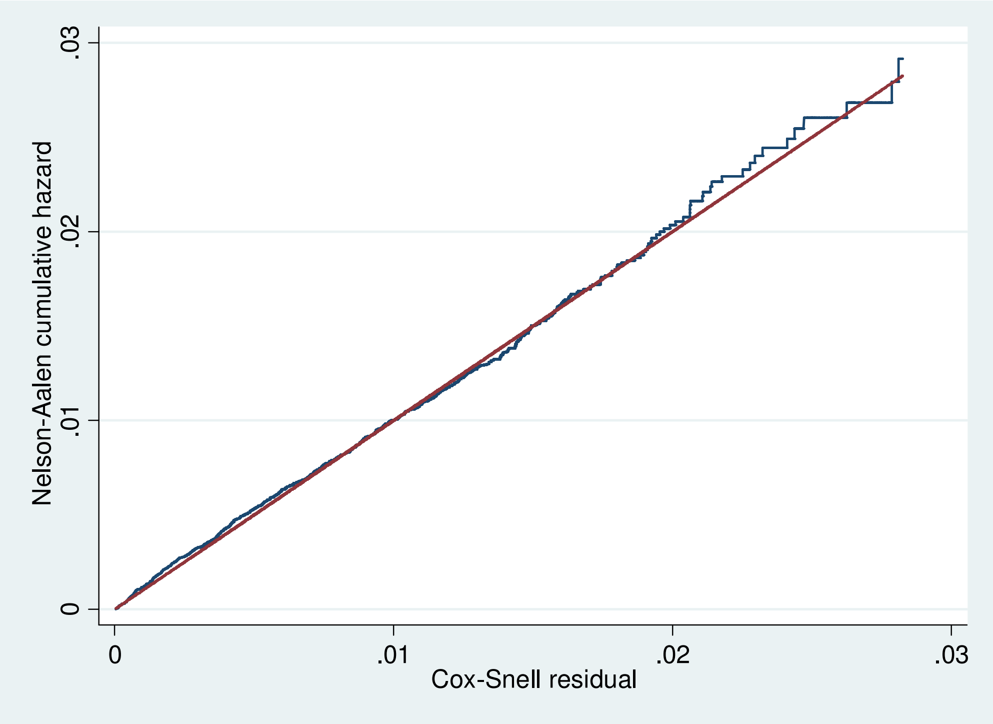

STROBE Statement—checklist of items that should be included in reports of observational studies

	Item No	Recommendation
**Title and abstract**	1	(*a*) Indicate the study's design with a commonly used term in the title or the abstract
		(*b*) Provide in the abstract an informative and balanced summary of what was done and what was found
**Introduction**		
Background/rationale	2	Explain the scientific background and rationale for the investigation being reported
Objectives	3	State specific objectives, including any prespecified hypotheses
**Methods**		
Study design	4	Present key elements of study design early in the paper
Setting	5	Describe the setting, locations, and relevant dates, including periods of recruitment, exposure, follow-up, and data collection
Participants	6	(*a*) *Cohort study*—Give the eligibility criteria, and the sources and methods of selection of participants. Describe methods of follow-up<br>*Case-control study*—Give the eligibility criteria, and the sources and methods of case ascertainment and control selection. Give the rationale for the choice of cases and controls<br>*Cross-sectional study*—Give the eligibility criteria, and the sources and methods of selection of participants
		(*b*) *Cohort study*—For matched studies, give matching criteria and number of exposed and unexposed<br>*Case-control study*—For matched studies, give matching criteria and the number of controls per case
Variables	7	Clearly define all outcomes, exposures, predictors, potential confounders, and effect modifiers. Give diagnostic criteria, if applicable
Data sources/ measurement	8*	For each variable of interest, give sources of data and details of methods of assessment (measurement). Describe comparability of assessment methods if there is more than one group
Bias	9	Describe any efforts to address potential sources of bias
Study size	10	Explain how the study size was arrived at
Quantitative variables	11	Explain how quantitative variables were handled in the analyses. If applicable, describe which groupings were chosen and why
Statistical methods	12	(*a*) Describe all statistical methods, including those used to control for confounding
		(*b*) Describe any methods used to examine subgroups and interactions
		(*c*) Explain how missing data were addressed
		(*d*) *Cohort study*—If applicable, explain how loss to follow-up was addressed<br>*Case-control study*—If applicable, explain how matching of cases and controls was addressed<br>*Cross-sectional study*—If applicable, describe analytical methods taking account of sampling strategy
		(*e*) Describe any sensitivity analyses

Continued on next page

For peer review only - http://bmjopen.bmj.com/site/about/guidelines.xhtml

**Results**		
Participants	13*	(a) Report numbers of individuals at each stage of study—eg numbers potentially eligible, examined for eligibility, confirmed eligible, included in the study, completing follow-up, and analysed
		(b) Give reasons for non-participation at each stage
		(c) Consider use of a flow diagram
Descriptive data	14*	(a) Give characteristics of study participants (eg demographic, clinical, social) and information on exposures and potential confounders
		(b) Indicate number of participants with missing data for each variable of interest
		(c) *Cohort study*—Summarise follow-up time (eg, average and total amount)
Outcome data	15*	*Cohort study*—Report numbers of outcome events or summary measures over time
		Case-control study—Report numbers in each exposure category, or summary measures of exposure
		Cross-sectional study—Report numbers of outcome events or summary measures
Main results	16	(*a*) Give unadjusted estimates and, if applicable, confounder-adjusted estimates and their precision (eg, 95% confidence interval). Make clear which confounders were adjusted for and why they were included
		(*b*) Report category boundaries when continuous variables were categorized
		(*c*) If relevant, consider translating estimates of relative risk into absolute risk for a meaningful time period
Other analyses	17	Report other analyses done—eg analyses of subgroups and interactions, and sensitivity analyses
**Discussion**		
Key results	18	Summarise key results with reference to study objectives
Limitations	19	Discuss limitations of the study, taking into account sources of potential bias or imprecision. Discuss both direction and magnitude of any potential bias
Interpretation	20	Give a cautious overall interpretation of results considering objectives, limitations, multiplicity of analyses, results from similar studies, and other relevant evidence
Generalisability	21	Discuss the generalisability (external validity) of the study results
**Other information**		
Funding	22	Give the source of funding and the role of the funders for the present study and, if applicable, for the original study on which the present article is based

*Give information separately for cases and controls in case-control studies and, if applicable, for exposed and unexposed groups in cohort and cross-sectional studies.

Note: An Explanation and Elaboration article discusses each checklist item and gives methodological background and published examples of transparent reporting. The STROBE checklist is best used in conjunction with this article (freely available on the Web sites of PLoS Medicine at http://www.plosmedicine.org/, Annals of Internal Medicine at http://www.annals.org/, and Epidemiology at http://www.epidem.com/). Information on the STROBE Initiative is available at www.strobe-statement.org.

For peer review only

For peer review only - http://bmjopen.bmj.com/site/about/guidelines.xhtml

Association of skirt size and postmenopausal breast cancer risk in older women: a cohort study within the UK Collaborative Trial of Ovarian Cancer Screening (UKCTOCS)

Journal:	*BMJ Open*
Manuscript ID:	bmjopen-2014-005400.R1
Article Type:	Research
Date Submitted by the Author:	24-Jun-2014

Complete List of Authors:	Fourkala, Evangelia-Ourania; University College London, Women's Cancer<br>Burnell, Matthew; University College London, Women's Cancer<br>Cox, Catharine; University College London, Women's Cancer<br>Ryan, Andy; University College London, Women's Cancer<br>Salter, Laura; University College London Hospitals NHS Foundation Trust,<br>Gentry-Maharaj, Aleksandra; University College London, Women's Cancer<br>Parmar , Mahesh ; University College London, Hub for Trials Methodology<br>Jacobs, Ian; University of Manchester,<br>Menon, Usha; University College London, Women's Cancer

**Primary Subject Heading**:	Oncology
Secondary Subject Heading:	Epidemiology
Keywords:	ONCOLOGY, Breast tumours < ONCOLOGY, Epidemiology < ONCOLOGY

SCHOLARONE™ Manuscripts

Title: Association of skirt size and postmenopausal breast cancer risk in older women: a cohort study within the UK Collaborative Trial of Ovarian Cancer Screening (UKCTOCS)

Authors: Evangelia-Ourania Fourkala[1]*, Matthew Burnell[1]*, Catherine Cox[1], Andy Ryan[1], Laura Currin Salter[2], Aleksandra Gentry-Maharaj[1], Mahesh Parmar[3], Ian Jacobs[1, 4], Usha Menon[1]**

*joint

**corresponding author

Affiliations:

[1]Women's Cancer, Institute for Women's Health, University College London, London, UK

[2]University College London Hospitals NHS Foundation Trust, London, UK

[3]MRC Clinical Trials Unit, London, UK

[4]Academic Health Science Centre, University of Manchester, Manchester, UK

Corresponding author: Usha Menon, Gynaecological Cancer Research Centre, Women's Cancer, Institute for Women's Health, University College London, 149 Tottenham Road, London W1T 7DN, UK. u.menon@ucl.ac.uk

For peer review only

For peer review only - http://bmjopen.bmj.com/site/about/guidelines.xhtml

Abstract:

Objectives: Several studies suggest that overall and central-obesity are associated with increased breast cancer (BC) risk in postmenopausal-women. However, there are no studies investigating changes of central obesity and BC. We report on the association of BC risk with self-reported skirt size (SS) (waist-circumference proxy) changes between twenties and postmenopausal-age.

Design: Prospective cohort-study.

Setting: UK Collaborative Trial of Ovarian Cancer Screening (UKCTOCS) involving the nine trial centres in England.

Participants: Postmenopausal-women aged>50 with no known history of BC prior to or on the day of completion of the study-entry questionnaire.

Interventions: At recruitment and at study-entry, women were asked to complete a questionnaire. Women were followed-up via 'flagging' at the NHS Information Centre in England and the Hospital Episode Statistics.

Main outcome-measure: Time to initial BC diagnosis.

Results: Between 2nd January '05 and 1st July '10, 92834 UKCTOCS participants (median age 64.0) completed the study-entry questionnaire. During median follow-up of 3.19 years (25th-75thcentile: 2.46-3.78), 1090 women developed BC. Univariable Cox models showed body mass index (BMI) at recruitment to UKCTOCS (Hazard Ratio (HR) for a 5 unit change=1.076, 95% CI: 1.012-1.136), current SS at study entry (HR=1.051; 95% CI: 1.014-1.089) and change in SS per 10-years (CSS) (HR=1.330; 95% CI: 1.121-1.579) were associated with increased BC risk but not SS at 25 (HR=1.006; 95% CI: 0.958-1.056). CSS was the most predictive singe adiposity measure and further analysis including both CSS and BMI in the model revealed CSS remained significant (HR=1.266; 95% CI: 1.041-1.538) but not BMI (HR=1.037; 95% CI: 0.970-1.109).

Conclusions: CSS is associated with BC risk independent of BMI. A unit increase in UK SS (e.g. 12 to

For peer review only - http://bmjopen.bmj.com/site/about/guidelines.xhtml

14) every 10-years between 25 and postmenopausal-age increases postmenopausal BC risk by 33%. Validation of these results could provide women with a simple and easy to understand message.

Strengths and limitations of this study:

- To the best of our knowledge, this is the first study investigating the association between central obesity using skirt size (SS) as a proxy and breast cancer (BC) risk. Between 25 and postmenopausal age, an increase in SS by one unit every decade increased the risk of postmenopausal BC by 33% while decrease in SS was associated with lowering of risk.
- Our prospective cohort-study includes 94,000 women with comprehensive follow-up through data linkage to multiple national databases.
- There is a possibility of underestimation of self-reported SS. However, if current SS at study-entry is uniformly underestimated then there is merely rescaling of CSS so that the strength of the association is unaffected. Furthermore, recall bias of the SS at 25 maybe a limitation but unless this inability in reporting is systematically related to future BC, measurement error can only result in underestimating the strength of the true association between CSS and BC risk.
- Given that obesity is now emerging as a global epidemic, from a public health prospective these findings are significant as they provide women with a simple and easy to understand message.

Keywords: breast cancer, risk, skirt size, waist circumference, central obesity, BMI, postmenopausal

(Manuscript word count: 3 824; No of tables: 3; Number of figures: 2)

For peer review only

For peer review only - http://bmjopen.bmj.com/site/about/guidelines.xhtml

Introduction

Breast cancer (BC) is a major global health concern with 1 384 000 women being diagnosed every year [1]. Significant advances in BC prevention have been accompanied by growing controversies regarding BC screening. Both highlight the need for better risk stratification. During the last few years a growing number of studies have reported on anthropometric factors, particularly on the relationship between BC risk and overall and/or central obesity [2-4].

A number of adiposity measures exist. The oldest and most common measure is body mass index (BMI) which is an empirical proxy for human body fat based on an individual's weight and height. Central fat is another measure of obesity. It is usually estimated by waist circumference (WC), waist-hip ratio (WHR), waist-height ratio (WHtR) or the conicity index which evaluates WC in relation to height and weight. An adequate and easy to use proxy measure for WC is clothing (trouser/skirt) size, which has been shown to provide a reliable and feasible estimate of WC at the population level [5]. It has been suggested that WC independent of BMI, maybe predictive of increased health risk [6].

There is a large body of evidence that high BMI is associated with increased BC risk in postmenopausal women and reduced risk in premenopausal women [3 4 7]. Another suggested BC risk predictor is adult weight gain, which typically reflects an increase in body fat [8-10]. Results concerning weight loss are more equivocal; there are studies that show no significant effect upon risk, while some suggest postmenopausal weight loss may be protective [11-15]. Furthermore, the association of central obesity with BC risk is unclear with studies reporting conflicting results [2 16]. Among postmenopausal women, several studies [17-24] but not all [14 25-29] have shown WC or WHR to be positively associated with increased BC risk [30]. There are, however, no studies examining changes in WC or WHR and BC risk.

For peer review only - http://bmjopen.bmj.com/site/about/guidelines.xhtml

The UK Collaborative Trial of Ovarian Cancer Screening (UKCTOCS) [31], with comprehensive follow-up, provides the opportunity to examine the association of body size changes with BC risk in a large prospective cohort. Furthermore, the availability of socioeconomic, reproductive, lifestyle and cancer family history data makes it possible to adjust for many potential confounders of breast risk.

Methods

Setting

UKCTOCS is a multicentre randomised controlled trial of ovarian cancer screening in the general population. The trial invited 1.2 million women of whom 202 638 were recruited between 17thApril 2001 and 27thSeptember 2005 through 13 trial centres in England, Wales and Northern Ireland. The detailed trial design and eligibility criteria have been described elsewhere [31]. All subjects completed a questionnaire at recruitment and a further postal follow-up questionnaire 3-4 years post-recruitment. The latter was the study-entry questionnaire for the current analysis. Follow-up was through a ‘flagging study’ with the Health and Social Care National Health Service (NHS) Information Centre who provided regular notifications of cancer registrations or deaths in the cohort. In addition, data linkage to the Hospital Episode Statistics (HES) an administrative database in England provided information on all inpatient and outpatient NHS hospital visits.

Women provided written consent for use of their data in secondary studies. Ethical approval was obtained from the Joint University College London (UCL)/UCL Hospital Committees on the Ethics of Human Research (REC reference:06/Q0505/102, June 2008).

For peer review only - http://bmjopen.bmj.com/site/about/guidelines.xhtml

Subjects

Participants recruited from England who returned the study-entry questionnaire prior to 2nd July 2010 (censorship date – see 'BC notification'). Women recruited from Northern Ireland and Wales were excluded as HES was only available for women residing in England.

Data collection

Questionnaires

<u>Recruitment questionnaire:</u> This was completed at recruitment into UKCTOCS and included information on the variables listed in Table 1A and 1B. This included height and weight used to calculate BMI, the postcode which was used to derive the Index of Multiple Deprivation (IMD) (with lower scores indicating less deprived areas), age at first and last period, number of pregnancies, hysterectomy, sterilisation, infertility and breast and ovarian cancer family history, past oral contraceptive pill use, current hormone replacement therapy (HRT) use.

<u>Study-entry questionnaire:</u> This was completed at follow-up (3-4 years after recruitment) and provided information on education, skirt size, continuing use of HRT, smoking, alcohol use, health status and cancer diagnosis post recruitment (Table 1A and 1B). The date of completion of this questionnaire was the entry date for the current study. This was available in 89.8% of the women. Where this date was missing, the date of questionnaire data entry into the trial management system was used.

BC notification

The ICD-9 and ICD-10 codes used to identify women with BC were 174* and C50*, respectively. Up-to-date data records from the NHS Information Centre and HES were searched to identify women with a BC diagnosis. Women also self-reported BC. Both at recruitment and at current study-entry,

For peer review only

For peer review only - http://bmjopen.bmj.com/site/about/guidelines.xhtml

women were asked specifically whether they had ever been diagnosed with BC and if so, details of diagnosis date and details of treating physician [32]. Overall there was a good concordance between the different sources used to identify the BC cases [32 33].

As there can be delays in cancer notification by the National Health and Social Care NHS Information Centre [32], the censorship date was set as 1stJuly 2010, two years prior to the date (10th July 2012) when data was provided by the NHS Information Centre agencies for this analysis. This ensured that as far as possible, all BCs in the stated period of observation were registered and therefore included. HES data was available for all the participants for the period between 6th of August 1998 when HES started and 31st March 2010.

Exposure variables

Self-reported height and weight were taken from the recruitment questionnaire and used to calculate BMI (with one unit equal here to 5kg/m^2). In the study-entry questionnaire two specific questions related to skirt size (SS) were asked: 1) 'What was your SS when you were in your twenties?' 2) 'What is your SS now?' Women could choose from 13 SS categories ranging from 6-30. The two SS questions were used to create a variable reflecting change in SS over time. The change in SS variable (CSS) was calculated as an increase in SS per 10 years. Note that a 'one unit' increase in SS means an increase by two nominal values, for example from size 10 to size 12 as odd sizes do not exist in the UK.

Statistical Analysis

Standard survival analysis methods were used to analyse the data. Women with BC previous to questionnaire completion date were excluded from the analysis. The date of study-entry questionnaire was the time-point used for entry into study. However, there was delayed entry (left

For peer review only - http://bmjopen.bmj.com/site/about/guidelines.xhtml

truncation) in that they were only able to participate in the current study because they 'survived' (had not died, withdrawn from trial or got BC) the period from their mid-twenties (the first skirt size question) to study-entry questionnaire completion date (the second current skirt size question). To account for a woman's contribution to the analysis being conditional on having 'survived' during the unobserved period, the length of which depends on the age at study entry, the timescale used was age rather than date. Therefore, the time origin was fixed at age=25 for all subjects and study entry was age at completion of study-entry questionnaire. Women were censored either at age at first BC, death from any cause or 1st July 2010 whichever occurred first.

Summaries of all identified potential confounding risk factors were performed. These variables were also included individually in a Cox regression model to obtain univariable estimates of their hazard ratio relating to BC risk. In addition kernel density plots of all four adiposity measures were created and Pearson correlation coefficients were calculated for all adiposity measure-pairings. To obtain an appropriate confounder-adjusted estimate of the relative hazard of BC of increasing SS, a Cox regression model including CSS in continuous form with all potential risk factors, regardless of statistical significance, was fitted (the 'full model'). The functional form for CSS was determined using fractional polynomials, by considering powers from the standard predefined set {-2, -1, -0.5, 0, 0.5, 1, 2, 3}, and modelling the most appropriate relationship between CSS and the (log) relative hazard. The full model was refitted with each of the adiposity measures forming the exposure variable, in turn. In addition, a variant formulation of the full model included both CSS and BMI and both skirt size at current study-entry and BMI.

Cox-Snell residuals were used to test the model fit and the proportional hazards assumption used in Cox regression was tested using the scaled Schoenfeld residuals for each variable in the final model. The (undue) influence of any outliers was assessed by calculating the change in the parameter

For peer review only - http://bmjopen.bmj.com/site/about/guidelines.xhtml

estimate when an observation is deleted. To visualise the 'dose-response' relationship model, predicted relative hazards with 95% confidence bands for a range of CSS values were plotted. To obtain estimates of absolute risk for certain scenarios (when CSS=0, CSS=1, CSS=median value), the event density function was assumed to be exponentially distributed, and the full model with CSS was fitted again using exponential regression. Survival curves with the same covariate pattern following both the Cox and exponential model fitting were plotted to ensure that the constant hazard was a reasonable assumption when averaged over the timeframe. An estimate of absolute risk was calculated by using the covariate-specific hazard from the exponential model for 1 year and for 5 years.

Sensitivity analyses

To guard against the possibility of reverse causality whereby preclinical BC may result in weight gain, the full model with CSS was refitted but with a time-lag of one year so that the start of the study period occurs one year after completion of the study-entry questionnaire.

In the primary analysis, missing data in CSS or any other confounder was handled by case-wise deletion. To assess the robustness of estimation with such an approach to potential biases, multiple imputation (MI) was used to calculate the corresponding model estimates with missing data imputed appropriately. Specifically, a multivariate normal distribution formed by all potential predictors and the outcome variables (event and time) was used for the imputed random draws, and 20 fully imputed datasets were created with which to obtain valid estimates and standard errors.

Results

157 997 women residing in England were recruited to the trial between 17thApril 2001 and 29thSeptember 2005. 11 659 (9 440 withdrew, 533 moved away, 1 686 died) women were not sent

For peer review only - http://bmjopen.bmj.com/site/about/guidelines.xhtml

the study-entry questionnaire. The remaining 146 338 women were sent the study-entry questionnaire between 28th June 2005 and 17th March 2009 and 112 945 (77.2%) responded by 31st December 2010. 10 689 of these women completed the current study-entry questionnaire after the censorship date of 1st July 2010 and were therefore excluded.

Eight women did not give consent for flagging. The remaining 102 249 were successfully flagged with the NHS Information Centre. Of these, 62 were lost to follow-up by the NHS Information Centre prior to questionnaire completion, 9 342 were diagnosed with BC before or on the day of completion of the study-entry questionnaire and in 10, study-entry questionnaire completion date could not be calculated. The remaining 92 834 women were included in the study and were followed-up for a median of 3.19 years (25th-75th centile: 2.46-3.78 years). During the study period, 1 090 women developed BC, resulting in an absolute risk of BC of 1.2% (1 090 / 92 834) in this cohort of postmenopausal women, 75% of whom were aged over 60 years with no prior history of BC.

The reproductive and lifestyle characteristics of the 92 834 women included in the analysis are summarised in Table 1A and B. Briefly, the women were mainly White, with a median year of birth at 1943 (Interquartile range (IQR): 1937-1947), 19.6% had a University degree and their median standardised - IMD (using 2008 data) was -0.327 (IQR: -0.685-0.458) indicating that most women are from less deprived areas. Most women were overweight (median BMI 25.598, (IQR: 23.203-28.821) at trial recruitment (median recruitment age 60.2; IQR: 55.8-65.8), had a median SS at 25 of 12 (IQR: 12-14) and at study-entry of 14 (IQR: 12-16) (median age 64.0; IQR: 59.7-69.7). The median CSS was 0.287 (IQR: 0.191-0.533) per 10 years, which is equivalent to an increase of 1 unit in SS (e.g. 10 to 12) in almost 35 years. Figure 1 shows the distribution for the four adiposity measures where CSS is largely symmetrical whilst the other three measures display some positive skewness.

For peer review only - http://bmjopen.bmj.com/site/about/guidelines.xhtml

The Pearson's correlation coefficients for the different adiposity measures are presented in Table 2. All of them were relatively high correlated apart from SS at 25 with CSS. Amongst the univariable Cox regressions, all measures of adiposity had significant hazard ratios (HRs) at the 5% level, except for SS at 25 (Table 1A and Table 1B). Other significant predictors of BC risk were age at last period, number pregnancies greater than 6 months, infertility treatment, family history of BC and HRT use at current study-entry (Table 1A and Table 1B).

5500 women were not included in the model due to missing values for either CSS or a confounder. The remaining 87 334 women, 1013 of whom developed BC during follow-up were included in the full Cox model with CSS. Use of fractional polynomials of the CSS variable showed the most appropriate functional form was linear, with tests of better fit for other powers of degrees 1 (p=0.719) and 2 (p=0.981) showing no improvement. Confounder-adjustment revealed a stronger positive association between CSS and BC risk (HR=1.330 per 10 years; 95%CI: 1.121-1.579; *p*=0.001) than without adjustment (Table 3). For each unit increase in SS per 10 years the hazard rate of BC increased by 33%. Replacement of CSS with the other adiposity measures in a full model, again showed a significant but lesser association between BMI (HR=1.076 per 5 units; 95%CI: 1.018-1.136; *p*=0.009) and SS at study-entry (HR=1.051; 95%CI: 1.014-1.089; *p*=0.006) with BC risk, but not for SS at 25 (HR=1.006; 95%CI: 0.958-1.056; *p*=0.809). Including recruitment BMI into the full model with CSS showed some sharing of predictive power but CSS had an association with BC risk independent of BMI (HR=1.27; 95%CI: 1.041-1.538; *p*=0.018 for CSS; HR=1.04; 95%CI: 0.970-1.109; *p=0.287* for recruitment BMI) (Table 3). A full model with SS at study-entry (HR=1.037; 95% CI: 0.992-1.085; *p=0.109*) and BMI (HR=1.037; 95% CI: 0.964-1.116; *p=0.329*) suggested less independence between the 2 measures than between CSS and BMI and neither variable was statistically significant at α=0.05 (Table 3).

For peer review only - http://bmjopen.bmj.com/site/about/guidelines.xhtml

Tests and plots based on the Schoenfeld residuals showed no evidence of any violation of the proportional hazards assumption. Supplementary Figure S1 plots these residuals for CSS with a superimposed smoother and shows that the effect of CSS on BC risk is essentially stable over the entire age range considered in this study. The Cox-Snell residuals plotted against their cumulative hazard showed a virtual line of unity indicating good model fit (figure not shown). Similar diagnostic results were found using the other adiposity measures.

Figure 2 shows how the model relates BC risk to varying changes in SS. For example, for those with an increase of 2 SS every 10 years, the estimated relative hazard increases by 77% (HR=1.769; 95%CI: 1.164-2.375). Supplementary Figure S2 shows the estimated survival functions when CSS=0, CSS=1 and CSS=0.287 (the median CSS value), with other continuous covariates set to their mean value and categorical variables set to their base value, and starting at the earliest observed age entering the study. These functions are plotted for both the Cox and exponential model and show that the exponential model is a reasonable approximation over the timeframe considered here. The derived estimates of absolute risk for 1 year were 0.33% (95%CI: 0.30-0.36%) when CSS=0 and 0.43% (95%CI: 0.37-0.48%) when CSS=1 (exponential model HR=1.31). These estimates compare with 0.35% (95%CI: 0.33-0.38%) when CSS=0.287 (median CSS) and the raw incidence rate of 0.38% (1090 cancers in 3.07 mean study years). The 5 year absolute risks are 1.63% (95%CI: 1.48-1.77) when CSS=0 and 2.14% (95%CI: 1.87%-2.40%) when CSS=1 and a raw incidence rate (number of cancers per 5 years of study time across sample) of 1.91%.

A sensitivity analysis added a time-lag of one year to the primary analysis that used the full model with CSS. For this regression model only 86 284 women were included as 1 121 women were left-censored before one year, including 71 who had missing covariate data. The HR of 1.422 (95% CI:

For peer review only - http://bmjopen.bmj.com/site/about/guidelines.xhtml

1.149-1.758; p=0.001) is slightly larger compared to the primary analysis but not substantively different (Table 3).

A multiple imputation analysis was carried out to investigate the effect of missing data on the model estimation. Data were missing for one or more variables in 5.9% and for CSS in 1.2% of the sample. Twenty completed datasets were created using multiple imputation and the subsequent HR=1.300 (95% CI: 1.102-1.534; p=0.002) (Table 3) and standard error estimate for CSS was essentially unchanged compared to the case-wise model result.

Discussion

This is to date the only study that we are aware of investigating the association of change in SS and BC risk. Our data suggests that a unit increase in UK SS (e.g. 12 to 14) every 10 years between the twenties and postmenopausal age increases the risk of postmenopausal BC by 33%. We estimated an increase in 5-year absolute risk of postmenopausal BC from 1 in 61 to 1 in 51 with each unit increase in SS per 10 years. Reductions in SS decreased the risk of BC (Figure 2) though it should be noted that for 76% of the women in this study, SS increased over their adult lifetime. These findings may provide women with a simple and easy to understand message given that SS has been found to be a reliable measure for changes in WC and one that women may relate and understand better in comparison to other adiposity measures such as BMI [5],

The major strengths of this study are its prospective cohort design, sample size, comprehensive follow-up through national registries and administrative databases and self-reporting resulting in high quality data. Evaluation of numerous possible confounders including life style choices and socioeconomic status minimised bias. Due to missing data in the covariates, case-wise deletion meant 5508 women were dropped from the Cox regression model. However, a sensitivity analysis using 20 completed sets

For peer review only - http://bmjopen.bmj.com/site/about/guidelines.xhtml

of multiply imputed data showed that the missing data had little effect on the overall outcome of the study. Furthermore, the study conclusions were maintained after introducing a time-lag of one year to the study entry to rule out reverse causality. The absolute risk of BC in this cohort over a median follow-up of 3.2 years was 1.2% as only postmenopausal women with no prior history of BC, 75% of whom were aged over 60 years, were included in this analysis. In keeping with other studies, family history of BC, infertility, age at last period, HRT use were associated with increased BC risk while pregnancies lasting over 6 months were protective [34]. Limitations of the study are the relatively short follow-up period, the possibility of underestimation of self-reported SS and recall bias of SS at 25. However, if current SS is uniformly underestimated across the sample then there is merely a rescaling of the CSS variable so that the strength of the relationship is unaffected, although the HR relating to a unit increase would be smaller. The same scenario applies to possible modern downsizing in SS to reflect increasing BMI (for example. a SS of 12 in their mid-twenties is now equivalent to, say, a SS of 10) assuming that this downsizing trend does not noticeably occur over the period of 'current study-entry SS'. All dates of FU questionnaire were between 2005 and 2010 with 97% between 2006 and 2009. The true relationship would only be weaker than reported if underestimation was not random but more prevalent amongst those who got BC compared to those that did. Furthermore, unless the inability to accurately recall SS in the twenties is systematically related to future BC or a confounder, measurement error can only result in underestimating the strength of the true relationship between CSS and risk of BC.

The findings that CSS is associated with BC risk (CSS per 10 years HR=1.33, 95%CI: 1.12-1.58; *p*=0.0010) and that addition of BMI to the model does not significantly improve risk prediction, are consistent with previous studies addressing the relationship between body weight and BC risk showing that adult weight gain is a better predictor of BC risk than absolute weight or BMI [8-10 30]. Increases in WC have also been reported to increase risk of other cancers such as pancreatic [35], endometrial

For peer review only - http://bmjopen.bmj.com/site/about/guidelines.xhtml

[36] and ovarian [37] cancer. A more equivocal issue in the literature is whether weight loss has a protective effect. Our findings that a decrease in SS is associated with decreased risk are in line with the studies showing reduction of weight can reduce risk of postmenopausal BC [11 12].

Current SS (at postmenopausal age) (HR=1.05, 95%CI: 1.01-1.09), but not SS in the twenties, was associated with a statistically significant increase in BC risk and appears to be a better predictor compared to BMI. Our findings come in agreement with other studies [30] and in keeping with the findings of a comparison of adiposity measures in postmenopausal women where WC was found to be a stronger risk factor than BMI for a variety of conditions [38]. Absolute WC has also been associated with increased risk of other cancers such as lung [39] and colon [40].

In our study changes in SS was a better predictor of BC risk compared to absolute SS in terms of strength of association. Although the exact mechanism of these relationships need to be better understood, there is a suggestion that body fat around the waist is more metabolically active than adipose tissue elsewhere [41]. Obesity is known to increase oestrogen levels as a result of aromatisation of androstenedione in adipose tissue as well as affect insulin resistance and chronic inflammation, well known factors shown to increase BC risk [42].

Conclusion

Convincing evidence is available that high body weight, BMI and adult weight gain, which typically reflect an increase in body fat, are associated with increased BC risk. To the best of our knowledge, this is the first study investigating the association of waist changes using skirt size as a proxy and BC risk. Between twenties and postmenopausal age, an increase in skirt size by one unit every decade increases the risk of postmenopausal BC by 33%. Validation of these results could provide women with a simple and easy to understand message.

For peer review only

Acknowledgements

The authors are particularly grateful to the women throughout the UK who are participating in the trial and to the entire medical, nursing and administrative staff who work on UKCTOCS.

Footnotes

Author's contributions

UM, EOF and MB designed the study. EOF, CC, LCS and UM undertook the literature research. EOF, MB and UM drafted the manuscript. MB and EOF prepared the figures and tables and MB undertook the statistical analysis. All authors were involved in data collection. EOF, CC and LCS were involved in data cleaning. All authors contributed to interpretation of the data and critically revised the manuscript and approved the final version.

Funding

UKCTOCS was core funded by the Medical Research Council, Cancer Research UK and National Institute of Health Research (NIHR) with additional support from the Eve Appeal and the NIHR Biomedical Research Centre at UCLH/UCL. The views expressed in the publication are those of the authors and not necessarily those of the funders. The funding source or the sponsor had no role in data collection, data analysis, data interpretation, or writing of the report. The researchers are independent from the funders.

Competing interests

UM and IJ have a financial interest through Abcodia Ltd in the third party exploitation of clinical trials biobanks, which have been developed through the research at UCL. During part of the trial IJ had a consultancy arrangement with Becton Dickinson in the field of tumour markers.

For peer review only

Ethical approval

The study was approved by the Joint University College London/University College London Hospital Committees on the Ethics of Human Research (REC reference: 06/Q0505/102, June 2008). Approvals for the use of hospital episode statistics data were obtained as part of the standard hospitals episode statistics approval process.

Patient Consent

Obtained

Provenance and peer review

Not commissioned; externally peer reviewed.

Data sharing statement

No additional data are available.

For peer review only

For peer review only - http://bmjopen.bmj.com/site/about/guidelines.xhtml

References:

1 http://globocan.iarc.fr/factsheet.asp.

2 Harvie M, Hooper L, Howell AH. Central obesity and breast cancer risk: a systematic review. Obes Rev 2003;4(3):157-73.

3 Friedenreich CM. Review of anthropometric factors and breast cancer risk. Eur J Cancer Prev 2001;10(1):15-32.

4 Wiseman M. The second World Cancer Research Fund/American Institute for Cancer Research expert report. Food, nutrition, physical activity, and the prevention of cancer: a global perspective. Proc Nutr Soc 2008;67(3):253-6.

5 Hughes LAE, Schouten LJ, Goldbohm RA, et al. Self-reported Clothing Size as a Proxy Measure for Body Size. Epidemiology 2009;20(5):673-76.

6 Battram DS, Beynon C, He M. The reliability and validity of using clothing size as a proxy for waist circumference measurement in adults. Applied physiology, nutrition, and metabolism = Physiologie appliquee, nutrition et metabolisme 2011;36(2):183-90.

7 Cheraghi Z, Poorolajal J, Hashem T, et al. Effect of body mass index on breast cancer during premenopausal and postmenopausal periods: a meta-analysis. PLoS One 2012;7(12):e51446.

8 Eliassen AH, Colditz GA, Rosner B, et al. Adult weight change and risk of postmenopausal breast cancer. Jama 2006;296(2):193-201.

9 Lahmann PH, Schulz M, Hoffmann K, et al. Long-term weight change and breast cancer risk: the European prospective investigation into cancer and nutrition (EPIC). Br J Cancer 2005;93(5):582-9.

10 Magnusson C, Baron J, Persson I, et al. Body size in different periods of life and breast cancer risk in post-menopausal women. Int J Cancer 1998;76(1):29-34.

For peer review only - http://bmjopen.bmj.com/site/about/guidelines.xhtml

11 Eliassen AH, Colditz GA, Rosner B, et al. Adult weight change and risk of postmenopausal breast cancer. JAMA 2006;296(2):193-201.

12 Parker ED, Folsom AR. Intentional weight loss and incidence of obesity-related cancers: the Iowa Women's Health Study. Int J Obes Relat Metab Disord 2003;27(12):1447-52.

13 Radimer KL, Ballard-Barbash R, Miller JS, et al. Weight change and the risk of late-onset breast cancer in the original Framingham cohort. Nutrition and cancer 2004;49(1):7-13.

14 Morimoto LM, White E, Chen Z, et al. Obesity, body size, and risk of postmenopausal breast cancer: the Women's Health Initiative (United States). Cancer Causes Control 2002;13(8):741-51.

15 Barnes-Josiah D, Potter JD, Sellers TA, et al. Early body size and subsequent weight gain as predictors of breast cancer incidence (Iowa, United States). Cancer Causes Control 1995;6(2):112-8.

16 Connolly BS, Barnett C, Vogt KN, et al. A meta-analysis of published literature on waist-to-hip ratio and risk of breast cancer. Nutr Cancer 2002;44(2):127-38.

17 Huang Z, Willett WC, Colditz GA, et al. Waist circumference, waist : hip ratio, and risk of breast cancer in the Nurses' Health Study. Am J Epidemiol 1999;150(12):1316-24.

18 Mannisto S, Pietinen P, Pyy M, et al. Body-size indicators and risk of breast cancer according to menopause and estrogen-receptor status. Int J Cancer 1996;68(1):8-13.

19 Ogundiran TO, Huo D, Adenipekun A, et al. Case-control study of body size and breast cancer risk in Nigerian women. Am J Epidemiol 2010;172(6):682-90.

20 Wu AH, Yu MC, Tseng CC, et al. Body size, hormone therapy and risk of breast cancer in Asian-American women. Int J Cancer 2007;120(4):844-52.

21 Kaaks R, Van Noord PA, Den Tonkelaar I, et al. Breast-cancer incidence in relation to height, weight and body-fat distribution in the Dutch "DOM" cohort. Int J Cancer 1998;76(5):647-51.

For peer review only - http://bmjopen.bmj.com/site/about/guidelines.xhtml

For peer review only

22 Nemesure B, Wu SY, Hennis A, et al. Body size and breast cancer in a black population--the Barbados National Cancer Study. Cancer Causes Control 2009;20(3):387-94.

23 Folsom AR, Kushi LH, Anderson KE, et al. Associations of general and abdominal obesity with multiple health outcomes in older women: the Iowa Women's Health Study. Arch Intern Med 2000;160(14):2117-28.

24 Fagherazzi G, Chabbert-Buffet N, Fabre A, et al. Hip circumference is associated with the risk of premenopausal ER-/PR- breast cancer. Int J Obes (Lond) 2012;36(3):431-9.

25 Muti P, Stanulla M, Micheli A, et al. Markers of insulin resistance and sex steroid hormone activity in relation to breast cancer risk: a prospective analysis of abdominal adiposity, sebum production, and hirsutism (Italy). Cancer Causes Control 2000;11(8):721-30.

26 Sonnenschein E, Toniolo P, Terry MB, et al. Body fat distribution and obesity in pre- and postmenopausal breast cancer. Int J Epidemiol 1999;28(6):1026-31.

27 Lahmann PH, Hoffmann K, Allen N, et al. Body size and breast cancer risk: findings from the European Prospective Investigation into Cancer And Nutrition (EPIC). Int J Cancer 2004;111(5):762-71.

28 den Tonkelaar I, Seidell JC, Collette HJ, et al. Obesity and subcutaneous fat patterning in relation to breast cancer in postmenopausal women participating in the Diagnostic Investigation of Mammary Cancer Project. Cancer 1992;69(11):2663-7.

29 Petrek JA, Peters M, Cirrincione C, et al. Is body fat topography a risk factor for breast cancer? Ann Intern Med 1993;118(5):356-62.

30 Pischon T, Nothlings U, Boeing H. Obesity and cancer. Proc Nutr Soc 2008;67(2):128-45.

For peer review only

For peer review only - http://bmjopen.bmj.com/site/about/guidelines.xhtml

31 Menon U, Gentry-Maharaj A, Ryan A, et al. Recruitment to multicentre trials--lessons from UKCTOCS: descriptive study. Bmj 2008;337:a2079.

32 Fourkala EO, Gentry-Maharaj A, Burnell M, et al. Histological confirmation of breast cancer registration and self-reporting in England and Wales: a cohort study within the UK Collaborative Trial of Ovarian Cancer Screening. Br J Cancer 2012;106(12):1910-6.

33 Gentry-Maharaj A, Fourkala EO, Burnell M, et al. Concordance of National Cancer Registration with self-reported breast, bowel and lung cancer in England and Wales: a prospective cohort study within the UK Collaborative Trial of Ovarian Cancer Screening. Br J Cancer 2013;109(11):2875-9.

34 Veronesi U, Boyle P, Goldhirsch A, et al. Breast cancer. Lancet 2005;365(9472):1727-41.

35 Aune D, Greenwood DC, Chan DS, et al. Body mass index, abdominal fatness and pancreatic cancer risk: a systematic review and non-linear dose-response meta-analysis of prospective studies. Ann Oncol 2012;23(4):843-52.

36 Yang TY, Cairns BJ, Allen N, et al. Postmenopausal endometrial cancer risk and body size in early life and middle age: prospective cohort study. Br J Cancer 2012;107(1):169-75.

37 Canchola AJ, Chang ET, Bernstein L, et al. Body size and the risk of ovarian cancer by hormone therapy use in the California Teachers Study cohort. Cancer Causes Control 2010;21(12):2241-8.

38 Hartz A, He T, Rimm A. Comparison of adiposity measures as risk factors in postmenopausal women. J Clin Endocrinol Metab 2012;97(1):227-33.

39 Kabat GC, Kim M, Hunt JR, et al. Body mass index and waist circumference in relation to lung cancer risk in the Women's Health Initiative. Am J Epidemiol 2008;168(2):158-69.

40 Moore LL, Bradlee ML, Singer MR, et al. BMI and waist circumference as predictors of lifetime colon cancer risk in Framingham Study adults. Int J Obes (Lond) 2004;28(4):559-67.

For peer review only

41 Bjorntorp P. "Portal" adipose tissue as a generator of risk factors for cardiovascular disease and diabetes. Arteriosclerosis 1990;10(4):493-6.

42 Calle EE, Kaaks R. Overweight, obesity and cancer: epidemiological evidence and proposed mechanisms. Nat Rev Cancer 2004;4(8):579-91.

For peer review only

For peer review only - http://bmjopen.bmj.com/site/about/guidelines.xhtml

Tables

Table 1A. Baseline characteristics and hazard ratios for breast cancer for adiposity measures and potential confounders (continuous variables).

**Factor**	**Mean**	**Standard deviation (SD)**	**25th percentile**	**50th percentile**	**75th percentile**	**Hazard Ratio (HR)**	**Lower 95%CI**	**Upper 95%CI**	**p-value**
Skirt size at age 25 years	12.509	2.533	12	12	14	1.008	0.962	1.056	0.736
Skirt size at study-entry	15.133	3.452	12	14	16	1.045	**1.011**	**1.081**	**0.01**
CSS	0.334	0.359	0.191	0.287	0.533	1.283	**1.09**	**1.51**	**0.003**
BMI	26.449	4.783	23.203	25.598	28.821	1.061	**1.006**	**1.119**	**0.029**
IMD	0.011	1.01	-0.685	-0.327	0.458	1.008	0.952	1.068	0.778
Age at Last Period	48.975	6.009	46.264	49.977	52.703	1.024	**1.014**	**1.035**	**<0.001**
Age at First Period	12.959	1.607	12	13	14	1.02	0.983	1.06	0.293

CSS=Change of skirt size per 10 years
BMI=Body mass index at recruitment (5kg/m2)
CI=Confidence interval

For peer review only - http://bmjopen.bmj.com/site/about/guidelines.xhtml

Table 1B. Baseline characteristics and hazard ratios for breast cancer for potential confounders (categorical variables).

Categorical variable		All Women N (%)	Hazard ratio	Lower 95%CI	Upper 95%CI	p-value
Ethnicity	White	90,011 (97.35)	ref			
	Black	832 (0.90)	1.307	0.622	2.75	0.48
	Asian	490 (0.53)	0.827	0.443	1.541	0.549
	Chinese	166 (0.18)	0.554	0.078	3.936	0.555
	Other	669 (0.72)	0.693	0.288	1.668	0.413
Education	No O-Level	56,856 (61.24)	ref			
	O-Level	35,986 (38.76)	1.208	1.07	1.363	0.002
	No A-level	78,436 (84.48)	ref			
	A-Level	14,406 (15.52)	1.204	1.03	1.407	0.02
	No Clerical or commercial qualification	67,075 (72.25)	ref			
	Clerical or commercial qualification	25,767 (27.75)	1.085	0.953	1.236	0.219
	No Nursing or teaching	79,668 (85.81)	ref			
	Nursing or teaching	13,174 (14.19)	1.051	0.888	1.243	0.565
	No Degree college/University	74,640 (80.39)	ref			
	Degree college/University	18,202 (19.61)	1	0.859	1.163	0.996
	No other than above qualification	64,090 (69.03)	ref			
	Other than above qualification	28,752 (30.97)	0.828	0.724	0.947	0.006

For peer review only

For peer review only - http://bmjopen.bmj.com/site/about/guidelines.xhtml

Hysterectomy with ovarian conservation at recruitment	No	76,318 (82.20)	ref			
	Yes	16,524 (17.80)	0.961	0.821	1.125	0.621
Oral contraceptive pill use	No	37,083 (39.94)	ref			
	Yes	55,759 (60.06)	0.97	0.852	1.106	0.654
Hormone replacement therapy use at recruitment	No	73, 544 (79.21)	Ref			
	Yes	19,298 (20.79)	1.12	0.971	1.289	0.118
Hormone replacement therapy use at study-entry	No	85,866 (92.50)	ref			
	Yes	6,963 (7.50)	1.232	**1**	**1.517**	**0.049**
Sterilisation	No	74,550 (80.30)	ref			
	Yes	18,292 (19.70)	0.951	0.817	1.108	0.523
Infertility treatment	No	72,731 (78.34)	ref			
	Yes	20,111 (21.66)	1.459	**1.103**	**1.93**	**0.008**
Pregnancies less than 6 months	0	64,126 (69,90)	ref			
	1	18,896 (20.60)	1.124	0.971	1.302	0.116
	2	5,596 (6.10)	0.966	0.742	1.256	0.795
	3	1,790 (1.95)	1.521	**1.055**	**2.192**	**0.024**
	4	649 (0.71)	1.690	0.955	2.990	0.071
	5	678 (0.74)	0.957	0.454	2.014	0.907
Pregnancies more than 6 months	0	10,986 (11.87)	ref			
	1	11,297 (12.18)	1.023	0.821	1.276	0.837
	2	40,890 (44.17)	0.768	**0.640**	**0.923**	**0.005**
	3	20,007 (21.61)	0.767	**0.624**	**0.944**	**0.012**
	4	6,612 (7.14)	0.824	0.626	1.084	0.625
	5	2,804 (3.03)	0.594	0.386	0.915	0.385
Alcohol (units per week) at study entry	None	21,086 (22.97)				
	Less than 1	16,084 (17.52)	1.011	0.835	1.226	0.905

For peer review only - http://bmjopen.bmj.com/site/about/guidelines.xhtml

	1 to 3	18,717 (20.39)	1.062	0.886	1.275	0.511
	4 to 6	13,813 (15.05)	1.039	0.851	1.267	0.71
	7 to 10	11,056 (12.04)	0.949	0.762	1.181	0.64
	11 to 15	6,549 (7.13)	0.985	0.758	1.279	0.91
	16 to 30	2,990 (3.26)	1.271	0.92	1.745	0.146
	21+	1,501 (1.64)	1.331	0.866	2.044	0.192
Smoking	0	51,470 (56.27)	ref			
	250	21,661 (23.68)	0.958	0.825	1.113	0.574
	500	9,632 (10.53)	1.032	0.847	1.259	0.752
	750	8,706 (9.52)	1.153	0.947	1.403	0.156
Relatives Breast Cancer History	No	72, 731 (78.34)	ref			
	Yes	20,111 (21.66)	1.48	**1.298**	**1.687**	**<0.0005**
Relatives Ovarian Cancer History	No	88,878 (95.73)	ref			
	Yes	3,964 (4.27)	0.946	0.699	1.279	0.718

For peer review only - http://bmjopen.bmj.com/site/about/guidelines.xhtml

Table 2. Pearson correlation coefficients between skirt size at 25, and at study entry (follow-up questionnaire completion), change in skirt size (CSS) per 10 years and body mass index (BMI) at recruitment.

	Skirt size at 25	Skirt size at study-entry	BMI at recruitment	CSS
Skirt size at 25	1.000			
Skirt size at study-entry	0.606 (p<0.0005)	1.000		
BMI	0.330 (p<0.0005)	0.643 (p<0.0005)	1.000	
CSS	-0.153 (p<0.0005)	0.693 (P<0.0005)	0.497 (p<0.0005)	1.000

For peer review only

Table 3. Confounder-adjusted Cox model estimates for various adiposity measures and their association with breast cancer risk.

Measures of adiposity	Effective sample size	Number of events	Hazard ratio	Lower 95%CI	Upper 95%CI	p-value
full model with individual adiposity measure						
CSS	87334	1013	1.33	1.121	1.579	0.001
Skirt size at 25	87783	1017	1.006	0.958	1.056	0.829
Skirt size at study-entry	87661	1018	1.051	**1.014**	**1.089**	0.006
BMI at recruitment	87615	1021	1.076	**1.012**	**1.136**	0.009
full model with specific adiposity measure combinations						
CSS	86678	1008	1.266	1.041	1.538	0.018
BMI			1.037	0.97	1.109	0.287
Skirt size at study-entry	87003	1013	1.037	0.992	1.085	0.109
BMI			1.037	0.964	1.116	0.329
full model with one year time lag						
CSS	86284	650	1.422	**1.149**	**1.758**	0.001
full model with multiple imputation model						
CSS	92834	1090	1.3	1.102	1.534	0.002

CSS=Change of skirt size per 10 years
BMI=Body mass index at recruitment per 5kg/m2
CI=Confidence interval
Full model means adjusted for all potential confounders: Index of Multiple Deprivation (IMD), age at first and last period, number of pregnancies, hysterectomy, sterilisation, infertility, breast and ovarian cancer family history, oral contraceptive use (pill) use, hormone replacement therapy (HRT) use (from recruitment questionnaire); education, skirt size, continuing use of HRT, smoking, alcohol use, health status and cancer diagnosis (from study-entry questionnaire)

For peer review only - http://bmjopen.bmj.com/site/about/guidelines.xhtml

Table legends

Table 1A. Baseline characteristics and hazard ratios for BC of the identified factors (continuous variables).

Table 1B. Baseline characteristics and hazard ratios for BC of the identified factors (categorical variables).

Table 2. Correlation between skirt size (SS) at twenties, at follow-up (study-entry), change of SS (CSS) per 10 year and body mass index (BMI).

Table 3. Confounder-adjusted Cox model estimates for various adiposity measures and their association with breast cancer risk.

Figure legends

Figure 1. Relative hazards for a range of skirt size (SS) changes per 10 years (CSS) with 95% confidence bands.

Figure 2. Distribution for skirt size (SS) at twenties, skirt size at current entry-study, BMI at recruitment and change of skirt size (CSS) every 10 years.

Supplementary Figure Legends

Supplementary Figure S1. Plot of the scaled Schoenfeld residuals for change of skirt size (CSS) with smoother.

For peer review only - http://bmjopen.bmj.com/site/about/guidelines.xhtml

Supplementary Figure S2. Estimated survival functions for 3 values of change of skirt SS (CSS) (CSS=0, CSS=1, CSS=0.287 (median CSS)) with other covariate values set to their mean value. Solid lines represent estimated curves based on the Cox model, dashed lines represent estimated curves based on the exponential model.

For peer review only

For peer review only - http://bmjopen.bmj.com/site/about/guidelines.xhtml

Title: Association of skirt size and postmenopausal breast cancer risk in older women: a cohort study within the UK Collaborative Trial of Ovarian Cancer Screening (UKCTOCS)

Formatted: Font: +Body (Calibri), 11 pt

Formatted: Justified

Authors: Evangelia-Ourania Fourkala[1]*, Matthew Burnell[1]*, Catherine Cox[1], Andy Ryan[1], Laura Currin Salter[2], Aleksandra Gentry-Maharaj[1], Mahesh Parmar[3], Ian Jacobs[1, 4], Usha Menon[1**]

*joint; **

**corresponding author

Formatted: Font: +Body (Calibri), 11 pt

Affiliations:

[1]Women's Cancer, Institute for Women's Health, University College London, London, UK

[2]University College London Hospitals NHS Foundation Trust, London, UK

[3]MRC Clinical Trials Unit, London, UK

[4]Academic Health Science Centre, University of Manchester, Manchester, UK

Corresponding author: Usha Menon, Gynaecological Cancer Research Centre, Women's Cancer, Institute for Women's Health, University College London, 149 Tottenham Road, London W1T 7DN, UK. u.menon@ucl.ac.uk

Formatted: Left, None, Space After: 0 pt, Line spacing: single

Formatted: Font: +Body (Calibri), 11 pt

For peer review only - http://bmjopen.bmj.com/site/about/guidelines.xhtml

Abstract:

Objectives: Several studies suggest that overall and central-obesity are associated with increased breast cancer (BC) risk in postmenopausal-women. However, there are no studies investigating changes of central obesity and BC. We report on the association of BC risk with self-reported skirt size (SS) (waist-circumference proxy) changes between twenties and ~~sixties~~postmenopausal-age.

Design: Prospective cohort-study.

Setting: UK Collaborative Trial of Ovarian Cancer Screening (UKCTOCS) involving the nine trial centres in England.

Participants: Postmenopausal-women aged>50 with no known history of ~~breast cancer~~BC prior to or on the day of completion of the study-entry questionnaire.

Interventions: At recruitment and at ~~follow-up (current~~ study-entry~~),~~, women were asked to complete a questionnaire. Women were followed-up via 'flagging' at the NHS Information Centre in England and the Hospital Episode Statistics.

Main outcome-measure: Time to initial ~~breast cancer~~BC diagnosis.

Results: Between 2nd January '05 and 1st July '10, 92834 ~~women~~UKCTOCS participants (median age 64.0) completed the ~~baseline~~study-entry questionnaire. During median follow-up of 3.19 years (25th-75th centile: 2.46-3.78), 1090 women developed BC. ~~Univariate analysis~~ Univariable Cox models showed body mass index (BMI) at recruitment to UKCTOCS (Hazard Ratio (HR~~)=~~) for a 5 unit change=1.~~01~~076, 95% CI: 1.~~00~~012-1.~~02, p=0.03),~~136), current SS at ~~baseline (sixties)~~study entry (HR=1.~~05~~051; 95% CI: 1.~~01~~014-1.~~08, p=0.01~~089) and change in SS per 10-years (CSS) (HR=1.~~38~~330; 95% CI: 1.~~13~~121-1.~~69, p=0.001) to be~~579) were associated with increased BC risk but not SS at ~~twenties~~25 (HR=1.~~01~~006; 95% CI: 0.~~96-1.06, p=0.74). Cox regression~~ 958-1.056). CSS was the most

2

Formatted: Font: +Body (Calibri), 11 pt

Formatted: Font: +Body (Calibri), 11 pt
Formatted: Font: +Body (Calibri), 11 pt
Formatted: Font: +Body (Calibri), 11 pt
Formatted: Font: +Body (Calibri), 11 pt
Formatted: Font: +Body (Calibri), 11 pt
Formatted: Font: +Body (Calibri), 11 pt
Formatted: Font: +Body (Calibri), 11 pt
Formatted: Font: +Body (Calibri), 11 pt
Formatted: Font: +Body (Calibri), 11 pt
Formatted: Font: +Body (Calibri), 11 pt
Formatted: Font: +Body (Calibri), 11 pt
Formatted: Font: +Body (Calibri), 11 pt
Formatted: Font: +Body (Calibri), 11 pt
Formatted: Font: +Body (Calibri), 11 pt
Formatted: Font: +Body (Calibri), 11 pt
Formatted: Font: +Body (Calibri), 11 pt
Formatted: Font: +Body (Calibri), 11 pt
Formatted: Font: +Body (Calibri), 11 pt
Formatted: Font: +Body (Calibri), 11 pt
Formatted: Font: +Body (Calibri), 11 pt
Formatted: Font: +Body (Calibri), 11 pt
Formatted: Font: +Body (Calibri), 11 pt
Formatted: Font: +Body (Calibri), 11 pt

For peer review only

predictive single adiposity measure and further analysis including both CSS and BMI in the model showed the covariate adjusted HR for revealed CSS to be the onlyremain significant obesity measure (HR=1.45 (266; 95% CI: 1.22041-1.71, p=0.001). 538) but not BMI (HR=1.037; 95% CI: 0.970-1.109).

Formatted: Font: +Body (Calibri), 11 pt
Formatted: Font: +Body (Calibri), 11 pt
Formatted: Font: +Body (Calibri), 11 pt
Formatted: Font: +Body (Calibri), 11 pt
Formatted: Font: +Body (Calibri), 11 pt
Formatted: Font: +Body (Calibri), 11 pt
Formatted: Font: +Body (Calibri), 11 pt

Conclusions: CSS is associated with BC risk independent of BMI. A unit increase in UK SS (e.g. 12 to 14) every 10-years between twenties25 and sixtiespostmenopausal-age increases postmenopausal BC risk by 4533%. Validation of these results could provide women with a simple and easy to understand message.

Formatted: Font: +Body (Calibri), 11 pt
Formatted: Font: +Body (Calibri), 11 pt
Formatted: Font: +Body (Calibri), 11 pt
Formatted: Font: +Body (Calibri), 11 pt
Formatted: Font: +Body (Calibri), 11 pt

Trial registration: International Standard Randomised Controlled Trial Number (ISRCTN)-22488978

Formatted: Justified

Strengths and limitations of this study:

- To the best of our knowledge, this is the first study investigating the association of waist changesbetween central obesity using skirt size (SS) as a proxy and breast cancer (BC) risk. Between 25 and postmenopausal age, an increase in SS by one unit every decade increased the risk of postmenopausal BC by 33% while decrease in SS was associated with lowering of risk.
- We report on aOur prospective cohort of study includes 94,000 women with comprehensive follow-up through data linkage to multiple national databases showing that between twenties and sixties, an increase in skirt size by one unit every decade increases the risk of postmenopausal breast cancer by 45% while decrease in skirt size is associated with lowering of risk..

Formatted: Font: +Body (Calibri), 11 pt
Formatted: Font: +Body (Calibri), 11 pt
Formatted: Font: +Body (Calibri), 11 pt
Formatted: Font: +Body (Calibri), 11 pt
Formatted: Space After: 10 pt, Adjust space between Latin and Asian text, Adjust space between Asian text and numbers
Formatted: Font: +Body (Calibri), 11 pt
Formatted: Font: +Body (Calibri), 11 pt
Formatted: Font: +Body (Calibri), 11 pt

For peer review only - http://bmjopen.bmj.com/site/about/guidelines.xhtml

- There is a possibility of underestimation of self-reported skirt size, howeverSS. However, if current SS at study-entry is uniformly underestimated then there is merely rescaling of CSS so that the strength of the association is unaffected. Furthermore, recall bias of the skirt size in the twentiesSS at 25 maybe a limitation but unless this inability in reporting is systematically related to future BC, measurement error can only result in underestimating the strength of the true association between CSS and BC risk.

- Given that obesity is now emerging as a global epidemic, from a public health prospective these findings are significant as they provide women with a simple and easy to understand message.

Formatted: Font: +Body (Calibri), 11 pt

Formatted: Font: +Body (Calibri), 11 pt

Formatted: Font: +Body (Calibri), 11 pt

Formatted: Font: +Body (Calibri), 11 pt

Formatted: Font: +Body (Calibri), 11 pt

Formatted: Font: +Body (Calibri), 11 pt, Font color: Auto, Condensed by 0.1 pt

Formatted: Justified, Space After: 10 pt, Adjust space between Latin and Asian text, Adjust space between Asian text and numbers, Tab stops: Not at 0.15" + 0.5"

Formatted: Font: +Body (Calibri), 11 pt

Formatted: Font: +Body (Calibri), 11 pt

Formatted: Font: +Body (Calibri), 11 pt

Formatted: Font: +Body (Calibri), 11 pt

Keywords: breast cancer, risk, skirt size, waist circumference, central obesity, BMI, postmenopausal

(Manuscript word count: 3 500824; No of tables: 53; Number of figures: 2)

For peer review only

Introduction

Breast cancer (BC) is a major global health concern with 1 384 000 women being diagnosed every year~~.[1]~~ [1]. Significant advances in BC prevention have been accompanied by growing controversies ~~surrounding~~regarding BC screening. Both highlight the need for better risk stratification. During the last few years a growing number of studies have reported on anthropometric factors, particularly on the relationship between BC risk and overall and/or central obesity~~.[2-4]~~ [2-4].

A number of adiposity measures exist. The oldest and most common measure is body mass index (BMI) which is an empirical proxy for human body fat based on an individual's weight and height. Central fat is another measure of obesity. It is usually estimated by waist circumference (WC), waist-hip ratio (WHR), waist-height ratio (WHtR) or the conicity index which evaluates WC in relation to height and weight. An adequate and easy to use proxy measure for WC is clothing (trouser/skirt) size, which has been ~~suggested to be independent of BMI.[5] It has also been~~ shown to ~~be~~provide a reliable and feasible ~~measure to obtain an~~ estimate of WC at the population level ~~and to predict the percentage~~[5]. It has been suggested that WC independent of BMI, maybe predictive of ~~the population at~~ increased health risk~~.[6]~~ [6].

There is a large body of evidence that high BMI is associated with increased BC risk in postmenopausal women and reduced risk in premenopausal women~~.[3, 4, 7]~~ [3 4 7]. Another suggested BC risk predictor is adult weight gain, which typically reflects an increase in body fat~~.[8-10]~~ [8-10]. Results concerning weight loss are more equivocal; there are studies that show no significant effect upon risk, while some suggest postmenopausal weight loss may be protective~~.[11-15]~~ [11-15]. Furthermore, the association of central obesity with BC risk is unclear with studies reporting conflicting results~~.[2, 16]~~ ~~Among postmenopausal~~

Formatted: Font: +Body (Calibri), 11 pt

Formatted: Font: +Body (Calibri), 11 pt

Formatted: Font: +Body (Calibri), 11 pt

Formatted: Font: +Body (Calibri), 11 pt

Formatted: Font: +Body (Calibri), 11 pt

Formatted: Font: +Body (Calibri), 11 pt

Formatted: Font: +Body (Calibri), 11 pt

Formatted: Font: +Body (Calibri), 11 pt

Formatted: Font: +Body (Calibri), 11 pt

Formatted: Font: +Body (Calibri), 11 pt

Formatted: Font: +Body (Calibri), 11 pt

Formatted: Font: +Body (Calibri), 11 pt

Formatted: Font: +Body (Calibri), 11 pt

Formatted: Font: +Body (Calibri), 11 pt

Formatted: Font: +Body (Calibri), 11 pt

Formatted: Font: +Body (Calibri), 11 pt

Formatted: Font: +Body (Calibri), 11 pt

Formatted: Font: +Body (Calibri), 11 pt

For peer review only

For peer review only - http://bmjopen.bmj.com/site/about/guidelines.xhtml

women, several studies [17-24] but not all [14, 25-29] have shown WC or WHR to be positively associated with increased BC risk.[30] [2 16]. Among postmenopausal women, several studies [17-24] but not all [14 25-29] have shown WC or WHR to be positively associated with increased BC risk [30]. There are, however, no studies examining changes in WC or WHR and BC risk.

Formatted: Font: +Body (Calibri), 11 pt

The UK Collaborative Trial of Ovarian Cancer Screening (UKCTOCS) [31][31], with comprehensive follow-up, provides the opportunity to examine the association of body size changes with BC risk in a large prospective cohort. Furthermore, the availability of socioeconomic, reproductive, life stylelifestyle and cancer family history data makes it possible to determine whether these factors moderate or potentially affect the associationadjust for many potential confounders of body changes with BCbreast risk.

Formatted: Font: +Body (Calibri), 11 pt

Formatted: Font: +Body (Calibri), 11 pt

Formatted: Font: +Body (Calibri), 11 pt

Formatted: Font: +Body (Calibri), 11 pt

Formatted: Font: +Body (Calibri), 11 pt

Formatted: Font: +Body (Calibri), 11 pt

Formatted: Font: +Body (Calibri), 11 pt

Formatted: Font: +Body (Calibri), 11 pt, Not Bold

Formatted: Font: +Body (Calibri), 11 pt

For peer review only - http://bmjopen.bmj.com/site/about/guidelines.xhtml

Methods

Setting

UKCTOCS is a multicentre randomised controlled trial of ovarian cancer screening in the general population. The trial invited 1.2 million women of whom 202 638 were recruited between 17th April 2001 and 27th September 2005 through 13 trial centres in England, Wales and Northern Ireland. The detailed trial design and eligibility criteria have been described elsewhere~~.~~[31] [31]. All subjects ~~were followed up~~completed a questionnaire at recruitment and a further postal follow-up questionnaire 3-4 years post-recruitment. The latter was the study-entry questionnaire for the current analysis. Follow-up was through a 'flagging study' with the Health and Social Care National Health Service (NHS) Information Centre ~~providing~~who provided regular ~~notification~~notifications of ~~any~~ cancer registrations or deaths in the cohort. In addition, data linkage to the Hospital Episode Statistics (HES) an administrative database in England provided information on all ~~women were sent a follow-up questionnaire (current study-entry questionnaire) 3-4 years after recruitment.~~ inpatient and outpatient NHS hospital visits.

Women provided written consent for use of their data in secondary studies. Ethical approval was obtained from the Joint University College London (UCL)/UCL Hospital Committees on the Ethics of Human Research (REC reference:06/Q0505/102, June 2008).

Subjects

Participants recruited from England who returned the ~~follow-up (current~~ study-entry~~)~~ questionnaire prior to 2nd July 2010 (censorship date~~).~~ – see 'BC notification'). Women recruited from Northern

Formatted: Font: +Body (Calibri), 11 pt

Formatted: Font: +Body (Calibri), 11 pt

Formatted: Font: +Body (Calibri), 11 pt

Formatted: Font: +Body (Calibri), 11 pt

Formatted: Font: +Body (Calibri), 11 pt

Formatted: Font: +Body (Calibri), 11 pt

Formatted: Font: +Body (Calibri), 11 pt

Formatted: Font: +Body (Calibri), 11 pt, Condensed by 0.1 pt

Formatted: Font: +Body (Calibri), 11 pt

Formatted: Font: +Body (Calibri), 11 pt

Formatted: Font: +Body (Calibri), 11 pt, Bold, Italic

Formatted: Font: +Body (Calibri), 11 pt

Formatted: Font: +Body (Calibri), 11 pt

Formatted: Font: +Body (Calibri), 11 pt

Formatted: Font: +Body (Calibri), 11 pt

For peer review only - http://bmjopen.bmj.com/site/about/guidelines.xhtml

Ireland and Wales were excluded as Hospital Episode Statistics(HES) was only available for women residing in England.

Data collection

Questionnaires

Recruitment questionnaire: This was completed at recruitment to the trialinto UKCTOCS and included information on variablethe variables listed in Table 1A and 1B. This included height and weight which was used to calculate BMI and, the postcode which was used to derive the Index of Multiple Deprivation (IMD). The latter is the official measure of deprivation in England and includes multiple domains such as income, employment, health and disability, education, skills and training, barriers to housing and services, living environment and crime) (with lower scores indicating less deprived areas), age at first and last period, number of pregnancies, hysterectomy, sterilisation, infertility and breast and ovarian cancer family history, past oral contraceptive pill use, current hormone replacement therapy (HRT) use.

Follow-up (current study

Study-entry) questionnaire: AtThis was completed at follow-up two specific questions related to skirt size(SS) were asked: (1) What was your SS when you were in your twenties? (2) What is your SS now? Women could choose from 13 SS categories ranging from 6-30. Information(3-4 years after recruitment) and provided information on education, lifestyle factorsskirt size, continuing use of HRT, smoking, alcohol use, health status and cancer diagnosis post recruitment as listed in (Table 1A and 1B were also collected.

BC notification

For the purpose). The date of completion of this analysis, up-to-date data was provided by the NHS Information Centre on 10th July 2012. HES data was also obtainedquestionnaire was the

Formatted: Font: +Body (Calibri), 11 pt
Formatted: Font: +Body (Calibri), 11 pt
Formatted: Font: +Body (Calibri), 11 pt, English (U.K.)
Formatted: Font: +Body (Calibri), 11 pt, Underline, English (U.K.)
Formatted: Level 1, Space After: 0 pt
Formatted: Font: +Body (Calibri), 11 pt, English (U.K.)
Formatted: Font: +Body (Calibri), 11 pt
Formatted: Font: +Body (Calibri), 11 pt
Formatted: Font: +Body (Calibri), 11 pt
Formatted: Font: +Body (Calibri), 11 pt
Formatted: Font: +Body (Calibri), 11 pt
Formatted: Font: +Body (Calibri), 11 pt
Formatted: Font: +Body (Calibri), 11 pt, Underline
Formatted: Font: +Body (Calibri), 11 pt, Underline
Formatted: Font: +Body (Calibri), 11 pt
Formatted: Font: +Body (Calibri), 11 pt
Formatted: Font: +Body (Calibri), 11 pt
Formatted: Font: +Body (Calibri), 11 pt
Formatted: Font: +Body (Calibri), 11 pt
Formatted: Font: +Body (Calibri), 11 pt
Formatted: Font: +Body (Calibri), 11 pt

For peer review only

entry date for allthe current study. This was available in 89.8% of the women. Where this date was missing, the participants in England for the period between 6th of August 1998 when HES started and 31st of March 2010 when the last uploaddate of questionnaire data entry into the trial management system was obtained. used.

Formatted: Font: +Body (Calibri), 11 pt

Formatted: Font: +Body (Calibri), 11 pt

Formatted: Font: +Body (Calibri), 11 pt

BC notification

The ICD-9 and ICD-10 codes used to identify women with BC were 174* and C50*, respectively. Up-to-date data records from the NHS Information Centre and HES were searched to identify women with a BC diagnosis. Women also self-reported BC. Both at recruitment and at current study-entry, women were asked specifically whether they had ever been diagnosed with BC and if so, details of diagnosis date and details of treating physician.[32] [32]. Overall there was a good concordance between the different sources used to identify the BC cases [32 33].

Formatted: Font: +Body (Calibri), 11 pt

Formatted: Font: +Body (Calibri), 11 pt

Formatted: Font: Calibri, 11 pt

As there can be delays in cancer notification by the National Health and Social Care NHS Information Centre [32], the censorship date was set as 1st July 2010, two years prior to the date (10th July 2012) when data was provided by the NHS Information Centre agencies for this analysis. This ensured that as far as possible, all BCs in the stated period of observation were registered and therefore included. HES data was available for all the participants for the period between 6th of August 1998 when HES started and 31st March 2010.

Formatted: Font: +Body (Calibri), 11 pt

Exposure variables

Self-reported height and weight were taken from the recruitment questionnaire and used to calculate BMI (with one unit equal here to 5kg/m^2). In the study-entry questionnaire two specific questions related to skirt size (SS) were asked: 1) 'What was your SS when you were in your twenties?' 2) 'What is your SS now?' Women could choose from 13 SS categories ranging from 6-30.

Formatted: Font: +Body (Calibri), 11 pt

For peer review only - http://bmjopen.bmj.com/site/about/guidelines.xhtml

Statistical Analysis

The two SS questions were used to create a variable reflecting change in SS over time. The change in SS variable (CSS) was calculated as an increase in SS per 10 years. Note that a 'one unit' increase in SS means an increase by two nominal values, for example from size 10 to size 12. The date of completion of the study entry questionnaire was the date used for time of current SS. This was available in 89.8% of the women and when this date was unknown (missing data) then the date of questionnaire entry into the trial management system was used. Women were asked to record SS in their twenties, so this time point was approximated by the date of their 25th birthday. Therefore, women were considered to be 'at risk' from WC changes from the age of 25. Thereafter, until date of study entry questionnaire, there is 'delayed entry' or 'left-truncation'. This takes into account the fact that a woman's contribution to the analysis is conditional on having survived (from developing BC for the first time) until date of questionnaire. Women with BC previous to study entry questionnaire completion date were excluded from the analysis. Equating the date for a given age (here, when age=25) with onset of risk means that the analysis is already age-adjusted so that there is no need for age as an additional covariate as odd sizes do not exist in the UK.

Formatted: Font: +Body (Calibri), 11 pt

Formatted: Font: +Body (Calibri), 11 pt

Formatted: Font: Calibri, 11 pt

As there can be delays in cancer notification by the National Health and Social Care NHS Information Centre [32], the censorship date was set as 1st July 2010, two years prior to the date (10th of July 2012) when data was provided by the NHS Information Centre agencies. This ensured that as far as possible, all BCs in the stated period of observation were identified. Those who died or were lost to follow-up during the study period were censored at that time point.

Formatted: Font: +Body (Calibri), 11 pt

Formatted: Font: +Body (Calibri), 11 pt

Statistical Analysis

For peer review only - http://bmjopen.bmj.com/site/about/guidelines.xhtml

Standard survival analysis methods were used to analyse the data. Women with BC previous to questionnaire completion date were excluded from the analysis. The date of study-entry questionnaire was the time-point used for entry into study. However, there was delayed entry (left truncation) in that they were only able to participate in the current study because they 'survived' (had not died, withdrawn from trial or got BC) the period from their mid-twenties (the first skirt size question) to study-entry questionnaire completion date (the second current skirt size question). To account for a woman's contribution to the analysis being conditional on having 'survived' during the unobserved period, the length of which depends on the age at study entry, the timescale used was age rather than date. Therefore, the time origin was fixed at age=25 for all subjects and study entry was age at completion of study-entry questionnaire. Women were censored either at age at first BC, death from any cause or 1st July 2010 whichever occurred first.

Formatted: Font: +Body (Calibri), 11 pt

Summaries of all identified potential confounding risk factors were performed. These variables were also included individually in a Cox regression model to obtain univariable estimates of their hazard ratio relating to BC risk. The CSS was categorised into 4 groups (group 1=decrease in SS per 10 years; group 2=increase of 0 to 0.5 SS per 10 years; group 3=increase of 0.5 to 1 SS per 10 years; group 4=increase of 1+ SS per 10 years) and further summaries of the potential confounders created for each of the 4 groups (supplementary material). Pearson correlation coefficients were calculated for all pairings of the four waist size proxies: SS in their twenties, current SS, CSSIn addition kernel density plots of all four adiposity measures were created and Pearson correlation coefficients were calculated for all adiposity measure-pairings. To obtain an appropriate confounder-adjusted estimate of the relative hazard of BC of increasing SS, a Cox regression model including CSS in continuous form with all potential risk factors, regardless of statistical significance, was fitted (the 'full model'). The functional form for CSS was determined using fractional polynomials, by considering powers from the standard predefined set {-2, -1, -0.5, 0,

For peer review only - http://bmjopen.bmj.com/site/about/guidelines.xhtml

0.5, 1, 2, 3}, and modelling the most appropriate relationship between CSS and the (log) relative hazard. The full model was refitted with each of the adiposity measures forming the exposure variable, in turn. In addition, a variant formulation of the full model included both CSS and BMI and both skirt size at current study-entry and BMI.

Formatted: Font: +Body (Calibri), 11 pt

To obtain an appropriate estimate of the relative hazard of BC of increasing SS, a Cox regression model including CSS (uncategorised) and additional risk factors that remained significant at the 5% level following a backward stepwise elimination was fitted. Significance tests were based on robust standard errors that allowed for any residual correlational structure within regional centres. The functional form for CSS was determined by the use of fractional polynomials, taking powers from the restricted set (-2, -1, -0.5, 0, 0.5, 1, 2, 3), to determine the appropriate relationship between CSS and the (log) relative hazard. Cox-Snell residuals were used to test the model fit,

Cox-Snell residuals were used to test the model fit and the proportional hazards assumption used in Cox regression was tested using the scaled Schoenfeld residuals for each variable in the final model. The (undue) influence of any outliers was assessed by calculating the change in the parameter estimate when an observation is deleted, known as dfbetas. Predicted. To visualise the 'dose-response' relationship model, predicted relative hazards with 95% confidence bands for a range of CSS values were calculated given the estimated multivariable model, and plotted. To obtain estimates of absolute risk for certain scenarios, survival time (when CSS=0, CSS=1, CSS=median value), the event density function was assumed to be exponentially distributed, and the same termsfull model with CSS was fitted again in anusing exponential regression model. Survival curves with the same covariate pattern following both the Cox and exponential model fitting were plotted to ensure that the constant hazard was a reasonable assumption when averaged over the timeframe. An estimate of absolute risk was calculated by using the covariate-specific hazard from the exponential model for 1 year and for 5 years.

Formatted: Font: +Body (Calibri), 11 pt

Formatted: Font: +Body (Calibri), 11 pt

Formatted: Font: +Body (Calibri), 11 pt

Formatted: Font: +Body (Calibri), 11 pt

Formatted: Font: +Body (Calibri), 11 pt

Formatted: Font: +Body (Calibri), 11 pt

Formatted: Font: +Body (Calibri), 11 pt

Formatted: Font: +Body (Calibri), 11 pt

Formatted: Font: +Body (Calibri), 11 pt

For peer review only - http://bmjopen.bmj.com/site/about/guidelines.xhtml

Sensitivity analyses

To guard against the possibility of reverse causality whereby preclinical BC may result in weight gain, the full model with CSS was refitted but with a time-lag of one year so that the start of the study period occurs one year after completion of the study-entry questionnaire.

In the primary analysis, missing data in CSS or any other confounder was handled by case-wise deletion. To assess the robustness of estimation with such an approach to potential biases, multiple imputation (MI) was used to calculate the corresponding model estimates with missing data imputed appropriately. Specifically, a multivariate normal distribution formed by all potential predictors and the outcome variables (event and time) was used for the imputed random draws, and 20 fully imputed datasets were created with which to obtain valid estimates and standard errors.

Formatted: Font: +Body (Calibri), 11 pt

Formatted: Font: +Body (Calibri), 11 pt

Formatted: Font: +Body (Calibri), 11 pt

For peer review only - http://bmjopen.bmj.com/site/about/guidelines.xhtml

Results

157 997 women residing in England were recruited to the trial between 17th April 2001 and 29th September 2005. 11 659 (9 440 withdrew, 533 moved away, 1 686 died) women were not sent the study-entry ~~(UKCTOCS follow-up)~~ questionnaire. The remaining 146 338 women were sent the study-entry questionnaire between ~~28th June~~28th June 2005 and 17th ~~of~~ March 2009 and 112 945 (77.2%) responded by 31st December 2010. 10 689 of these women completed the current study-entry questionnaire after the censorship date of 1st July 2010 and were therefore excluded.

Eight women did not give consent for flagging. The remaining 102 249 were successfully flagged with the NHS Information Centre. Of these, ~~9 342 (9.1%) were excluded as they~~62 were lost to follow-up by the NHS Information Centre prior to questionnaire completion, 9 342 were diagnosed with BC before or on the day of completion of the study-entry questionnaire~~. In addition,~~ and in 10, study-entry questionnaire completion date could not be calculated~~ in 10 women and 62 were lost to follow-up by the NHS Information Centre prior to questionnaire completion.~~. The remaining 92 834 women ~~entered~~were included in the study and were followed-up for a median of 3.19 years (25th-75th centile: 2.46-3.78 years). During the study period, 1 090 ~~(1.2 %)~~ women developed BC, resulting in an absolute risk of BC of 1.2% (1 090 / 92 834) in this cohort of postmenopausal women, 75% of whom were aged over 60 years with no prior history of BC.

The reproductive and lifestyle characteristics of the 92 834 women included ~~into~~in the analysis are summarised in Table 1-A and B. Briefly, the women were mainly White, with a median year of birth at 1943 (Interquartile range (IQR): 1937-1947), 19.6% had a University degree and their median standardised - IMD (using 2008 data) was ~~14~~ 0.327 (IQR: ~~9-25).~~ 0.685-0.458) indicating that most women are from less deprived areas. Most women were overweight (median ~~recruitment~~ BMI 25.~~6,~~

Formatted: Font: +Body (Calibri), 11 pt
Formatted: Left, None
Formatted: Font: +Body (Calibri), 11 pt, Bold
Formatted: Font: +Body (Calibri), 11 pt
Formatted: Font: +Body (Calibri), 11 pt
Formatted: Font: +Body (Calibri), 11 pt
Formatted: Font: +Body (Calibri), 11 pt
Formatted: Font: +Body (Calibri), 11 pt, Not Superscript/ Subscript
Formatted: Font: +Body (Calibri), 11 pt
Formatted: Font: +Body (Calibri), 11 pt
Formatted: Font: +Body (Calibri), 11 pt
Formatted: Font: +Body (Calibri), 11 pt
Formatted: Font: +Body (Calibri), 11 pt
Formatted: Font: +Body (Calibri), 11 pt
Formatted: Font: +Body (Calibri), 11 pt
Formatted: Font: +Body (Calibri), 11 pt
Formatted: Font: +Body (Calibri), 11 pt, Condensed by 0.1 pt
Formatted: Font: +Body (Calibri), 11 pt
Formatted: Font: +Body (Calibri), 11 pt
Formatted: Font: Calibri, 11 pt
Formatted: Font: +Body (Calibri), 11 pt
Formatted: Font: +Body (Calibri), 11 pt
Formatted: Font: +Body (Calibri), 11 pt
Formatted: Font: +Body (Calibri), 11 pt
Formatted: Font: +Body (Calibri), 11 pt

For peer review only

For peer review only - http://bmjopen.bmj.com/site/about/guidelines.xhtml

598, (IQR: 23.~~2~~203-28.~~8~~821) at trial recruitment (median recruitment age 60.2 ~~(~~; IQR: 55.8-65.8~~)).~~), had a median SS ~~in the twenties~~at 25 of 12 (IQR: 12-14) and at study-entry of 14 (IQR: 12-16) (median age 64.0 ~~(~~; IQR: 59.7-69.7~~)).~~). The median CSS was 0.~~21~~287 (IQR: 0.~~20~~191-0.~~43~~533) per 10 years, which is equivalent to an increase of 1 unit in SS (e.g. 10 to 12) in ~~50~~almost 35 years. ~~At entry 91 717 number of women (Supplementary~~ Figure 1 shows the distribution for~~Tables S1A and S1B) provided information on~~ the four ~~groups of~~adiposity measures where CSS~~.~~ is largely symmetrical whilst the other three measures display some positive skewness.

~~The Pearson's correlation coefficient was *r*=0.61 between SS at twenties and at current study-entry questionnaire completion, *r*=0.33 between the SS at twenties and recruitment BMI, *r*=0.64 between SS at current study-entry questionnaire completion and recruitment BMI and *r*=0.50 between CSS and recruitment BMI (all *p*-values<0.0005) (Table 2). As SS at twenties was relatively uncorrelated with CSS (*r*= 0.15), it was also used as a potential predictor in the Cox model.~~

The Pearson's correlation coefficients for the different adiposity measures are presented in Table 2. All of them were relatively high correlated apart from SS at 25 with CSS. Amongst the univariable Cox regressions, all measures of adiposity had significant hazard ratios (HRs) at the 5% level, except for SS at ~~twenties, with CSS having the smallest p-value (*p*=0.001), compared to SS at current study-entry (*p*=0.01) and recruitment BMI (*p*=0.03)~~25 (Table 1A and Table 1B). Other significant predictors of BC risk ~~(*p*<0.05)~~ were age at last period, number pregnancies greater than 6 months, infertility treatment, family history of BC ~~in both mother and sister, number of pregnancies less than 6 months~~ and HRT use ~~(~~at current~~) at~~ study-entry~~. Only increases in pregnancies greater than 6 months had a protective effect~~ (Table 1A and Table 1B).

For peer review only - http://bmjopen.bmj.com/site/about/guidelines.xhtml

The final Cox model for CSS 5500 women were not included 91 368 women, with 1 069 BCs as 1 474 women had in the model due to missing values for either CSS or one of the included confounders. Therea confounder. The remaining 87 334 women, 1013 of whom developed BC during follow-up were included in the full Cox model with CSS. Use of fractional polynomials of the CSS variable showed the most appropriate functional form was a stronglinear, with tests of better fit for other powers of degrees 1 (p=0.719) and 2 (p=0.981) showing no improvement. Confounder-adjustment revealed a stronger positive association between CSS and BC risk (HR=1.45330 per 10 years,; 95%CI: 1.22121-1.71579; p<=0.0005001) than without adjustment (Table 3). For each unit increase in SS per 10 years the hazard rate of BC increased by 45%. Other predictors33%. Replacement of CSS with the other adiposity measures in a full model, again showed a significant at the 5% level were age at last period but lesser association between BMI (HR=1.02,076 per 5 units; 95%CI: 1.018-1.01-1.04136; p<=0.0005), infertility009) and SS at study-entry (HR=1.39,051; 95%CI: 1.01014-1.90089; p=0.04), maternal006) with BC historyrisk, but not for SS at 25 (HR=1.71,006; 95%CI:1.57-1.87; p<0.0005), number of pregnancies greater than six months (HR=0.91, 95%CI: 0.88-0.95; p<0.0005) (Table 3). SS at twenties was not retained. There were no interactions with CSS that improved the model. Adding958-1.056; p=0.809). Including recruitment BMI into the existing model did not improve prediction (p=0.137full model with CSS showed some sharing of predictive power but CSS had an association with BC risk independent of BMI (HR=1.27; 95%CI: 1.041-1.538; p=0.018 for CSS; HR=1.04; 95%CI: 0.970-1.109; p=0.287 for recruitment BMI, whilst CSS remained strongly) (Table 3). A full model with SS at study-entry (HR=1.037; 95% CI: 0.992-1.085; p=0.109) and BMI (HR=1.037; 95% CI: 0.964-1.116; p=0.329) suggested less independence between the 2 measures than between CSS and BMI and neither variable was statistically significant, p at α=0.001) (05 (Table 3).

Formatted: Font: +Body (Calibri), 11 pt
Formatted: Font: +Body (Calibri), 11 pt
Formatted: Font: +Body (Calibri), 11 pt
Formatted: Font: +Body (Calibri), 11 pt
Formatted: Font: +Body (Calibri), 11 pt
Formatted: Font: +Body (Calibri), 11 pt
Formatted: Font: +Body (Calibri), 11 pt
Formatted: Font: +Body (Calibri), 11 pt
Formatted: Font: +Body (Calibri), 11 pt
Formatted: Font: +Body (Calibri), 11 pt
Formatted: Font: +Body (Calibri), 11 pt
Formatted: Font: +Body (Calibri), 11 pt
Formatted: Font: +Body (Calibri), 11 pt
Formatted: Font: +Body (Calibri), 11 pt
Formatted: Font: +Body (Calibri), 11 pt
Formatted: Font: +Body (Calibri), 11 pt
Formatted: Font: +Body (Calibri), 11 pt
Formatted: Font: +Body (Calibri), 11 pt
Formatted: Font: +Body (Calibri), 11 pt
Formatted: Font: +Body (Calibri), 11 pt
Formatted: Font: +Body (Calibri), 11 pt
Formatted: Font: +Body (Calibri), 11 pt
Formatted: Font: +Body (Calibri), 11 pt
Formatted: Font: +Body (Calibri), 11 pt
Formatted: Font: +Body (Calibri), 11 pt
Formatted: Font: +Body (Calibri), 11 pt
Formatted: Font: +Body (Calibri), 11 pt
Formatted: Font: +Body (Calibri), 11 pt
Formatted: Font: +Body (Calibri), 11 pt

For peer review only

For peer review only - http://bmjopen.bmj.com/site/about/guidelines.xhtml

Tests and plots based on the Schoenfeld residuals showed no evidence of any violation of the proportional hazards assumption. Supplementary Table S2).

Table 4 and Figure 1 showS1 plots these residuals for CSS with a superimposed smoother and shows that the effect of CSS on BC risk is essentially stable over the entire age range considered in this study. The Cox-Snell residuals plotted against their cumulative hazard showed a virtual line of unity indicating good model fit (figure not shown). Similar diagnostic results were found using the other adiposity measures.

Figure 2 shows how the model relates BC risk to CSS.varying changes in SS. For example, for those with an increase of 2 SS every 10 years, the estimated relative hazard more than doubles (adjusted increases by 77% (HR 2.21=1.769; 95%CI: 1.40164-2.86).375). Supplementary Figure 2 givesS2 shows the estimated survival functions when CSS=0, CSS=1 and CSS=0.21287 (the median CSS value), with all other covariatecontinuous covariates set atto their mean value and categorical variables set to their base value, and starting at the earliest observed age entering the study. These functions are plotted for both the Cox and exponential model and show that the exponential model is a reasonable approximation over the timeframe considered here. The derived estimates of absolute risk for 1 year were 0.33% (95%CI: 0.30-0.3736%) when CSS=0 and 0.4843% (95%CI: 0.4237-0.5448%) when CSS=1 (exponential model HR=1.4531). These estimates compare with 0.3635% (95%CI: 0.33-0.3938%) when CSS=0.21287 (median CSS) and the raw incidence rate of 0.38% (1090 cancers in 3.07 mean study years). The 5 year absolute risks are 1.66% (95%CI: 1.47-1.84) when CSS=0 and 2.41% (95%CI: 2.11%-2.71%) when CSS=1 and a raw incidence rate of 1.91%. Tests and plots based on the Schoenfeld residuals showed no evidence of any violation of the proportional hazards assumption (plot for CSS – Supplementary Figure S1). The p-values for the test of the PH assumption for each of the five variables ranged from 0.584 to 0.888

Formatted: Font: +Body (Calibri), 11 pt

Formatted: Font: +Body (Calibri), 11 pt

Formatted: Font: +Body (Calibri), 11 pt

Formatted: Font: +Body (Calibri), 11 pt

Formatted: Font: +Body (Calibri), 11 pt

Formatted: Font: +Body (Calibri), 11 pt

Formatted: Font: +Body (Calibri), 11 pt

Formatted: Font: +Body (Calibri), 11 pt

Formatted: Font: +Body (Calibri), 11 pt

Formatted: Font: +Body (Calibri), 11 pt

Formatted: Font: +Body (Calibri), 11 pt

Formatted: Font: +Body (Calibri), 11 pt

Formatted: Font: +Body (Calibri), 11 pt

Formatted: Font: +Body (Calibri), 11 pt

Formatted: Font: +Body (Calibri), 11 pt

Formatted: Font: +Body (Calibri), 11 pt

Formatted: Font: +Body (Calibri), 11 pt

Formatted: Font: +Body (Calibri), 11 pt

Formatted: Font: +Body (Calibri), 11 pt

Formatted: Font: +Body (Calibri), 11 pt

Formatted: Font: +Body (Calibri), 11 pt

Formatted: Font: +Body (Calibri), 11 pt

Formatted: Font: +Body (Calibri), 11 pt

For peer review only

For peer review only - http://bmjopen.bmj.com/site/about/guidelines.xhtml

~~indicating the validity of the model. The Cox-Snell residuals plotted against their cumulative hazard showed a virtual line of unity indicating good model fit (Supplementary Figure S2). Use of fractional polynomials of the CSS variable showed the most appropriate functional form was linear, with tests of powers of degrees 1 and 2 giving p-values of over 0.7. The dfbetas calculated for each observation showed that only for 12 of them did the deletion result in a change of the log-hazard estimate of over 0.01 (approximately equal to a change in the HR of 0.01) with a maximum value of 0.017.~~63% (95%CI: 1.48-1.77) when CSS=0 and 2.14% (95%CI: 1.87%-2.40%) when CSS=1 and a raw incidence rate (number of cancers per 5 years of study time across sample) of 1.91%.

Formatted: Font: +Body (Calibri), 11 pt

A sensitivity analysis added a time-lag of one year to the primary analysis that used the full model with CSS. For this regression model only 86 284 women were included as 1 121 women were left-censored before one year, including 71 who had missing covariate data. The HR of 1.422 (95% CI: 1.149-1.758; *p*=0.001) is slightly larger compared to the primary analysis but not substantively different (Table 3).

A multiple imputation analysis was carried out to investigate the effect of missing data ~~in the independent variables~~ on the model estimation. Data were missing for one or more variables in ~~1.6~~5.9% and for CSS in ~~0.7~~1.2% of the sample. Twenty completed datasets were created using multiple imputation and the subsequent HR=1.300 (95% CI: 1.102-1.534; *p*=0.002) (Table 3) and standard error ~~estimates did not change substantively~~estimate for CSS was essentially unchanged compared to the ~~original model with~~ case-wise ~~deletion for missing values (Supplementary Table S3).~~model result.

Formatted: Font: +Body (Calibri), 11 pt

Formatted: Font: +Body (Calibri), 11 pt

Formatted: Font: +Body (Calibri), 11 pt

Formatted: Font: +Body (Calibri), 11 pt

Formatted: Font: +Body (Calibri), 11 pt

Formatted: Font: +Body (Calibri), 11 pt

Formatted: Font: +Body (Calibri), 11 pt

Formatted: Font: +Body (Calibri), 11 pt

Formatted: Font: +Body (Calibri), 11 pt, Bold, Condensed by 0.1 pt

For peer review only - http://bmjopen.bmj.com/site/about/guidelines.xhtml

Discussion

This is to date the only study that we are aware of investigating the association of change in SS and BC risk. Our data suggests that a unit increase in UK SS (e.g. 12 to 14) every 10 years between the twenties and ~~sixties~~postmenopausal age increases the risk of postmenopausal BC by ~~45%. In this cohort this translated into~~33%. We estimated an increase in 5~~-~~year absolute risk of postmenopausal BC from 1 in ~~60~~61 to 1 in ~~41~~51 with each unit increase in SS~~.~~ per 10 years. Reductions in SS decreased the risk of BC (Figure 2) though it should be noted that for 76% of the women in this study, SS increased over their adult lifetime. These findings may provide women with a simple and easy to understand message given that SS has been found to be a reliable measure for changes in WC and ~~a measure~~one that women may relate and understand better in comparison to other adiposity measures such as BMI~~.~~[5] ~~Use of fractional polynomials of the CSS variable strongly suggest that changes in SS are linearly related to the log hazard rate. This model implies that reductions in SS decrease the risk of BC in the manner plotted in Figure 1, though it should be noted that in almost 75% of the women in this study, SS increased over their adult lifetime.~~ [5],

The major strengths of this study are its prospective cohort design, sample size, comprehensive follow-up through national registries~~, HES~~ and administrative databases and self-reporting resulting in high quality data~~ and evaluation~~. Evaluation of numerous possible confounders including life style choices and socioeconomic status~~.~~ minimised bias. Due to missing data in the covariates, case~~-~~wise deletion meant ~~1 474~~5508 women were dropped from the Cox regression model. However, a sensitivity analysis using 20 completed sets of multiply imputed data showed that the missing data had little effect on the overall outcome of the study. Furthermore, the study conclusions were maintained after introducing a time-lag of one year to the study entry to rule out reverse causality. The absolute risk of BC in this cohort over a median follow-up of 3.2 years was 1.2% as only postmenopausal women ~~in their sixties~~ with no prior history of BC~~ were eligible for~~, 75% of whom were aged over 60 years,

Formatted: Font: +Body (Calibri), 11 pt
Formatted: Font: +Body (Calibri), 11 pt
Formatted: Font: +Body (Calibri), 11 pt
Formatted: Font: +Body (Calibri), 11 pt
Formatted: Font: +Body (Calibri), 11 pt
Formatted: Font: +Body (Calibri), 11 pt
Formatted: Font: +Body (Calibri), 11 pt
Formatted: Font: +Body (Calibri), 11 pt
Formatted: Font: +Body (Calibri), 11 pt
Formatted: Font: +Body (Calibri), 11 pt
Formatted: Font: +Body (Calibri), 11 pt
Formatted: Font: +Body (Calibri), 11 pt
Formatted: Font: +Body (Calibri), 11 pt
Formatted: Font: +Body (Calibri), 11 pt
Formatted: Font: +Body (Calibri), 11 pt
Formatted: Font: +Body (Calibri), 11 pt
Formatted: Font: +Body (Calibri), 11 pt
Formatted: Font: +Body (Calibri), 11 pt

For peer review only

were included in this analysis. In keeping with other studies, family history of BC, infertility, age at last period, HRT ~~use were associated with increased BC risk while pregnancies lasting over 6 months were protective.~~[33] use were associated with increased BC risk while pregnancies lasting over 6 months were protective [34]. Limitations of the study are the relatively short follow-up period, the possibility of underestimation of self-reported SS and recall bias of ~~the~~ SS ~~in the twenties~~at 25. However, if current SS is uniformly underestimated across the sample then there is merely a rescaling of the CSS variable so that the strength of the relationship is unaffected, although the HR relating to a unit increase would be smaller. The same scenario applies to possible modern downsizing in SS to reflect increasing BMI (for example. a SS of 12 in their mid-twenties is now equivalent to, say, a SS of 10) assuming that this downsizing trend does not noticeably occur over the period of 'current study-entry SS'. All dates of FU questionnaire were between 2005 and 2010 with 97% between 2006 and 2009. ~~For the~~The true relationship ~~to~~would only be weaker than reported ~~here would require that~~if underestimation was not random but more prevalent amongst those ~~that did not get~~who got BC compared to those that did. Furthermore, unless the inability to accurately recall SS in the twenties is systematically related to future BC or a confounder, measurement error can only result in underestimating the strength of the true relationship between CSS and risk of BC.

The findings that CSS is associated with BC risk (CSS per 10 years HR=1.~~45~~33, 95%CI: 1.~~22~~12-1.~~71~~58; p=0.~~0002~~0010) and that addition of BMI to the model does not significantly improve risk prediction, are consistent with previous studies addressing the relationship between body weight and BC risk showing that adult weight gain is a better predictor of BC risk than absolute weight or BMI~~.~~ ~~[8-10, 30]~~ [8-10 30]. Increases in WC have also been reported to increase risk of other cancers such as pancreatic ~~[34]~~ ~~,~~[35], endometrial ~~[35]~~[36] and ovarian ~~[36]~~[37] cancer. A more equivocal issue in the literature is whether weight loss has a protective effect. Our findings that a decrease in SS is associated with

Formatted: Font: +Body (Calibri), 11 pt
Formatted: Font: +Body (Calibri), 11 pt
Formatted: Font: +Body (Calibri), 11 pt
Formatted: Font: +Body (Calibri), 11 pt
Formatted: Font: +Body (Calibri), 11 pt
Formatted: Font: +Body (Calibri), 11 pt
Formatted: Font: +Body (Calibri), 11 pt
Formatted: Font: +Body (Calibri), 11 pt
Formatted: Font: +Body (Calibri), 11 pt
Formatted: Font: +Body (Calibri), 11 pt
Formatted: Font: +Body (Calibri), 11 pt
Formatted: Font: +Body (Calibri), 11 pt
Formatted: Font: +Body (Calibri), 11 pt
Formatted: Font: +Body (Calibri), 11 pt
Formatted: Font: +Body (Calibri), 11 pt
Formatted: Font: +Body (Calibri), 11 pt
Formatted: Font: +Body (Calibri), 11 pt
Formatted: Font: +Body (Calibri), 11 pt
Formatted: Font: +Body (Calibri), 11 pt
Formatted: Font: +Body (Calibri), 11 pt

For peer review only

decreased risk are in line with the studies showing reduction of weight can reduce risk of postmenopausal BC.[11, 12] [11 12].

AbsoluteCurrent SS in the sixties(at postmenopausal age) (HR=1.04505, 95%CI: 1.01101-1.081, p=0.010)09), but not SS in the twenties, was associated with a small statistically significant increase in BC risk and was shownappears to be a better predictor compared to BMI. Our findings come in agreement with other studies [30][30] and in keeping with the findings of a comparison of adiposity measures in postmenopausal women where WC was found to be a stronger risk factor than BMI for a variety of conditions.[37] [38]. Absolute WC has also been associated with increased risk of other cancers such as lung [38][39] and colon.[39] [40].

In our study changes in SS per 10 years was a better predictor of BC risk compared to absolute SSs. Although the exact mechanism of these relationships need to be better understood, there is a suggestion that body fat around the waist is more metabolically active than adipose tissue elsewhere.[40] Obesity is known to increase oestrogen levels as a result of aromatisation of androstenedione in adipose tissue as well as affect insulin resistance and chronic inflammation, well known factors shown to increase BC risk.[41]

We report for the first time that increase in central adiposity, as indicated by increase in SS per 10 years, is a significant risk factor for BC. Given that obesity is now emerging as a global epidemic, from a public health prospective these findings provide women with a simple and easy to understand message – between twenties and sixties, increase by one SS every 10 years increases the risk of postmenopausal BC by 45% while decrease in SS is associated with lowering of risk. Further validation of these findings is needed.

Formatted: Font: +Body (Calibri), 11 pt

Formatted: Font: +Body (Calibri), 11 pt

Formatted: Font: +Body (Calibri), 11 pt

Formatted: Font: +Body (Calibri), 11 pt

Formatted: Font: +Body (Calibri), 11 pt

Formatted: Font: +Body (Calibri), 11 pt

Formatted: Font: +Body (Calibri), 11 pt

Formatted: Font: +Body (Calibri), 11 pt

Formatted: Font: +Body (Calibri), 11 pt

Formatted: Font: +Body (Calibri), 11 pt

Formatted: Font: +Body (Calibri), 11 pt

Formatted: Font: +Body (Calibri), 11 pt

Formatted: Font: +Body (Calibri), 11 pt

Formatted: Font: +Body (Calibri), 11 pt

For peer review only - http://bmjopen.bmj.com/site/about/guidelines.xhtml

In our study changes in SS was a better predictor of BC risk compared to absolute SS in terms of strength of association. Although the exact mechanism of these relationships need to be better understood, there is a suggestion that body fat around the waist is more metabolically active than adipose tissue elsewhere [41]. Obesity is known to increase oestrogen levels as a result of aromatisation of androstenedione in adipose tissue as well as affect insulin resistance and chronic inflammation, well known factors shown to increase BC risk [42].

Formatted: Font: +Body (Calibri), 11 pt

Formatted: Tab stops: Not at 0.39"

Conclusion

Convincing evidence is available that high body weight, BMI and adult weight gain, which typically reflect an increase in body fat, are associated with increased ~~breast cancer~~BC risk. To the best of our knowledge, this is the first study investigating the association of waist changes using skirt size as a proxy and ~~breast cancer~~BC risk. Between twenties and ~~sixties~~postmenopausal age, an increase in skirt size by one unit every decade increases the risk of postmenopausal ~~breast cancer~~BC by ~~45~~33%. Validation of these results could provide women with a simple and easy to understand message.

Formatted: Font: +Body (Calibri), 11 pt

Formatted: Font: +Body (Calibri), 11 pt

Formatted: Font: +Body (Calibri), 11 pt

Formatted: Font: +Body (Calibri), 11 pt

Formatted: Font: +Body (Calibri), 11 pt

Formatted: Font: +Body (Calibri), 11 pt

Acknowledgements

The authors are particularly grateful to the women throughout the UK who are participating in the trial and to the entire medical, nursing and administrative staff who work on UKCTOCS.

Formatted: None, Space After: 10 pt, Tab stops: Not at 0.39"

Formatted: Font: +Body (Calibri), 11 pt, Not Bold

Formatted: Font: +Body (Calibri), 11 pt

Formatted: Font: +Body (Calibri), 11 pt, Bold

Footnotes

Formatted: Font: +Body (Calibri), 11 pt

Author's contributions

UM, EOF and MB designed the study. EOF, CC, LCS and UM undertook the literature research. EOF, MB and UM drafted the manuscript. MB and EOF prepared the figures and tables and MB undertook the statistical analysis. ~~EOF, AR, AGM, and UM~~All authors were involved in data collection. EOF, CC

Formatted: Font: +Body (Calibri), 11 pt

For peer review only - http://bmjopen.bmj.com/site/about/guidelines.xhtml

and LCS were involved in data cleaning. All authors contributed to interpretation of the data and critically revised the manuscript and approved the final version.

Funding

UKCTOCS was core funded by the Medical Research Council, Cancer Research UK and National Institute of Health Research (NIHR) with additional support from the Eve Appeal and the NIHR Biomedical Research Centre at UCLH/UCL. The views expressed in the publication are those of the authors and not necessarily those of the funders. The funding source or the sponsor had no role in data collection, data analysis, data interpretation, or writing of the report. The researchers are independent from the funders.

Formatted: Font: +Body (Calibri)

Formatted: Font: +Body (Calibri), 11 pt

Formatted: Tab stops: Not at 0.39"

Competing interests

UM and IJ have a financial interest through Abcodia Ltd in the third party exploitation of clinical trials biobanks, which have been developed through the research at UCL. During part of the trial IJ had a consultancy arrangement with Becton Dickinson in the field of tumour markers.

~~Patient Consent~~

~~Obtained~~

Ethical approval

The study was approved by the Joint University College London/University College London Hospital Committees on the Ethics of Human Research (REC reference: 06/Q0505/102, June 2008). Approvals for the use of hospital episode statistics data were obtained as part of the standard hospitals episode statistics approval process.

Patient Consent

Formatted: Font: +Body (Calibri), 11 pt

For peer review only - http://bmjopen.bmj.com/site/about/guidelines.xhtml

Obtained

Formatted: Font: +Body (Calibri), 11 pt, Not Expanded by / Condensed by

Formatted: Level 1

Provenance and peer review

Formatted: Font: +Body (Calibri), 11 pt

Not commissioned; externally peer reviewed.

Formatted: Font: +Body (Calibri), 11 pt, Not Bold

Data sharing statement

Formatted: Font: +Body (Calibri), 11 pt

No additional data are available.

For peer review only

For peer review only - http://bmjopen.bmj.com/site/about/guidelines.xhtml

References:

Formatted: Font: +Body (Calibri), 11 pt, Not Bold, English (U.K.)

~~1. http://globocan.iarc.fr/factsheet.asp.~~

1 http://globocan.iarc.fr/factsheet.asp.

2~~.~~ Harvie~~,~~ M~~.~~, Hooper~~,~~ L~~. &~~, Howell~~, A.H~~ AH. Central obesity and breast cancer risk: a systematic review. Obes Rev 2003;4~~:~~(3):157-~~173~~73.

3~~.~~ Friedenreich~~, C.M~~ CM. Review of anthropometric factors and breast cancer risk. Eur J Cancer Prev 2001;10~~:~~(1):15-32.

4~~.~~ Wiseman~~,~~ M. The second World Cancer Research Fund/American Institute for Cancer Research expert report. Food, nutrition, physical activity, and the prevention of cancer: a global perspective. Proc Nutr Soc 2008;67~~:~~(3):253-~~25~~66.

5~~.~~ Hughes~~, L.A.E.,~~ LAE, Schouten~~, L.J.,~~ LJ, Goldbohm~~, R.A.~~ RA, et al. Self-reported Clothing Size as a Proxy Measure for Body Size. Epidemiology 2009;20~~:~~(5):673-~~676.~~76.

6~~.~~ Battram~~, D.S.,~~ DS, Beynon~~,~~ C~~. &~~, He~~,~~ M. The reliability and validity of using clothing size as a proxy for waist circumference measurement in adults. Applied physiology, nutrition, and metabolism = Physiologie appliquee, nutrition et metabolisme 2011;36~~:~~(2):183-~~190~~90.

7~~.~~ Cheraghi~~,~~ Z~~.~~, Poorolajal~~,~~ J~~.~~, Hashem~~,~~ T~~.~~, et al. Effect of body mass index on breast cancer during premenopausal and postmenopausal periods: a meta-analysis. PLoS One 2012;7~~:~~(12):e51446.

8~~.~~ Eliassen~~, A.H.,~~ AH, Colditz~~, G.A.,~~ GA, Rosner~~,~~ B~~.~~, et al. Adult weight change and risk of postmenopausal breast cancer. Jama 2006;296~~:~~(2):193-201.

9~~.~~ Lahmann~~, P.H.,~~ PH, Schulz~~,~~ M~~.~~, Hoffmann~~,~~ K~~.~~, et al. Long-term weight change and breast cancer risk: the European prospective investigation into cancer and nutrition (EPIC). Br J Cancer 2005;93~~:~~(5):582-~~58~~99.

Formatted: Normal, Justified, Level 1, Indent: Left: 0", First line: 0"

For peer review only

For peer review only - http://bmjopen.bmj.com/site/about/guidelines.xhtml

10~~.~~ Magnusson~~,~~ C~~.,~~, Baron~~,~~ J~~.,~~, Persson~~,~~ I~~.~~, et al. Body size in different periods of life and breast cancer risk in post-menopausal women. Int J Cancer 1998;76~~:~~(1):29-34.

11~~.~~ Eliassen~~, A.H.,~~ AH, Colditz~~, G.A.,~~ GA, Rosner~~,~~ B~~.~~, et al. Adult weight change and risk of postmenopausal breast cancer. JAMA 2006;296~~:~~(2):193-201.

12~~.~~ Parker~~, E.D. &~~ ED, Folsom~~, A.R~~ AR. Intentional weight loss and incidence of obesity-related cancers: the Iowa Women's Health Study. Int J Obes Relat Metab Disord 2003;27~~:~~(12):1447-~~1452~~52.

13~~.~~ Radimer~~, K.L.,~~ KL, Ballard-Barbash~~,~~ R~~.,~~, Miller~~, J.S.~~ JS, et al. Weight change and the risk of late-onset breast cancer in the original Framingham cohort. Nutrition and cancer 2004;49~~:~~(1):7-13.

14~~.~~ Morimoto~~, L.M.,~~ LM, White~~,~~ E~~.,~~, Chen~~,~~ Z~~.~~, et al. Obesity, body size, and risk of postmenopausal breast cancer: the Women's Health Initiative (United States). Cancer Causes Control 2002;13~~:~~(8):741-~~751.~~51.

15~~.~~ Barnes-Josiah~~,~~ D~~.,~~, Potter~~, J.D.,~~ JD, Sellers~~, T.A.~~ TA, et al. Early body size and subsequent weight gain as predictors of breast cancer incidence (Iowa, United States). Cancer Causes Control 1995;6~~:~~(2):112-~~118.~~8.

16~~.~~ Connolly~~, B.S.,~~ BS, Barnett~~,~~ C~~.,~~, Vogt~~, K.N.~~ KN, et al. A meta-analysis of published literature on waist-to-hip ratio and risk of breast cancer. Nutr Cancer 2002;44~~:~~(2):127-~~138.~~38.

17~~.~~ Huang~~,~~ Z~~.,~~, Willett~~, W.C.,~~ WC, Colditz~~, G.A.~~ GA, et al. Waist circumference, waist : hip ratio, and risk of breast cancer in the Nurses' Health Study. Am J Epidemiol 1999;150~~:~~(12):1316-~~1324.~~24.

18~~.~~ Mannisto~~,~~ S~~.,~~, Pietinen~~,~~ P~~.,~~, Pyy~~,~~ M~~.~~, et al. Body-size indicators and risk of breast cancer according to menopause and estrogen-receptor status. Int J Cancer 1996;68~~:~~(1):8-13.

19~~.~~ Ogundiran~~, T.O.,~~ TO, Huo~~,~~ D~~.,~~, Adenipekun~~,~~ A~~.~~, et al. Case-control study of body size and breast cancer risk in Nigerian women. Am J Epidemiol 2010;172~~:~~(6):682-~~690~~90.

For peer review only

For peer review only - http://bmjopen.bmj.com/site/about/guidelines.xhtml

20. Wu, A.H., AH, Yu, M.C., MC, Tseng, C.C. CC, et al. Body size, hormone therapy and risk of breast cancer in Asian-American women. Int J Cancer 2007;120:(4):844-85252.

21. Kaaks, R., Van Noord, P.A., PA, Den Tonkelaar, I., et al. Breast-cancer incidence in relation to height, weight and body-fat distribution in the Dutch "DOM" cohort. Int J Cancer 1998;76:(5):647-651.51.

22. Nemesure, B., Wu, S.Y., SY, Hennis, A., et al. Body size and breast cancer in a black population--the Barbados National Cancer Study. Cancer Causes Control 2009;20:(3):387-39494.

23. Folsom, A.R., AR, Kushi, L.H., LH, Anderson, K.E. KE, et al. Associations of general and abdominal obesity with multiple health outcomes in older women: the Iowa Women's Health Study. Arch Intern Med 2000;160:(14):2117-212828.

24. Fagherazzi, G., Chabbert-Buffet, N., Fabre, A., et al. Hip circumference is associated with the risk of premenopausal ER-/PR- breast cancer. Int J Obes (Lond) 2012;36:(3):431-4399.

25. Muti, P., Stanulla, M., Micheli, A., et al. Markers of insulin resistance and sex steroid hormone activity in relation to breast cancer risk: a prospective analysis of abdominal adiposity, sebum production, and hirsutism (Italy). Cancer Causes Control 2000;11:(8):721-730.30.

26. Sonnenschein, E., Toniolo, P., Terry, M.B. MB, et al. Body fat distribution and obesity in pre- and postmenopausal breast cancer. Int J Epidemiol 1999;28:(6):1026-1031.31.

27. Lahmann, P.H., PH, Hoffmann, K., Allen, N., et al. Body size and breast cancer risk: findings from the European Prospective Investigation into Cancer And Nutrition (EPIC). Int J Cancer 2004;111:(5):762-77171.

For peer review only

28~~.~~ den Tonkelaar~~,~~ I~~.,~~, Seidell~~, J.C.,~~ JC, Collette~~, H.J.~~ HJ, et al. Obesity and subcutaneous fat patterning in relation to breast cancer in postmenopausal women participating in the Diagnostic Investigation of Mammary Cancer Project. Cancer 1992;69~~:~~(11):2663-~~2667.~~7.

29~~.~~ Petrek~~, J.A.,~~ JA, Peters~~,~~ M~~.,~~, Cirrincione~~,~~ C~~.~~, et al. Is body fat topography a risk factor for breast cancer? Ann Intern Med 1993;118~~:~~(5):356-~~362.~~62.

30~~.~~ Pischon~~,~~ T~~.,~~, Nothlings~~,~~ U~~. &~~, Boeing~~,~~ H. Obesity and cancer. Proc Nutr Soc 2008;67~~:~~(2):128-~~145~~45.

31~~.~~ Menon~~,~~ U~~.,~~, Gentry-Maharaj~~,~~ A~~.,~~, Ryan~~,~~ A~~.~~, et al. Recruitment to multicentre trials--lessons from UKCTOCS: descriptive study. Bmj 2008;337:a2079.

32~~.~~ Fourkala~~, E.O.,~~ EO, Gentry-Maharaj~~,~~ A~~.,~~, Burnell~~,~~ M~~.~~, et al. Histological confirmation of breast cancer registration and self-reporting in England and Wales: a cohort study within the UK Collaborative Trial of Ovarian Cancer Screening. Br J Cancer 2012;106~~:~~(12):1910-~~1916.~~6.

33~~.~~ Gentry-Maharaj A, Fourkala EO, Burnell M, et al. Concordance of National Cancer Registration with self-reported breast, bowel and lung cancer in England and Wales: a prospective cohort study within the UK Collaborative Trial of Ovarian Cancer Screening. Br J Cancer 2013;109(11):2875-9.

34 Veronesi~~,~~ U~~.,~~, Boyle~~,~~ P~~.,~~, Goldhirsch~~,~~ A~~.~~, et al. Breast cancer. Lancet 2005;365~~:~~(9472):1727-~~1741~~41.

~~34.~~ 35 Aune~~,~~ D~~.,~~, Greenwood~~, D.C.,~~ DC, Chan~~, D.S.~~ DS, et al. Body mass index, abdominal fatness and pancreatic cancer risk: a systematic review and non-linear dose-response meta-analysis of prospective studies. Ann Oncol 2012;23~~:~~(4):843-~~852~~52.

Formatted: Normal, Justified, Level 1, Indent: Left: 0", First line: 0"

For peer review only

For peer review only - http://bmjopen.bmj.com/site/about/guidelines.xhtml

~~35.~~ 36 Yang~~, T.Y.,~~ TY, Cairns~~, B.J.,~~ BJ, Allen~~,~~ N~~.,~~, et al. Postmenopausal endometrial cancer risk and body size in early life and middle age: prospective cohort study. Br J Cancer 2012;107~~:~~(1):169-~~175~~75.

~~36.~~ 37 Canchola~~, A.J.,~~ AJ, Chang~~, E.T.,~~ ET, Bernstein~~,~~ L~~.,~~, et al. Body size and the risk of ovarian cancer by hormone therapy use in the California Teachers Study cohort. Cancer Causes Control 2010;21~~:~~(12):2241-~~2248~~8.

~~37.~~ 38 Hartz~~,~~ A~~.,~~, He~~,~~ T~~. &~~, Rimm~~,~~ A. Comparison of adiposity measures as risk factors in postmenopausal women. J Clin Endocrinol Metab 2012;97~~:~~(1):227-~~233~~33.

~~38.~~ 39 Kabat~~, G.C.,~~ GC, Kim~~,~~ M~~.,~~, Hunt~~, J.R.~~ JR, et al. Body mass index and waist circumference in relation to lung cancer risk in the Women's Health Initiative. Am J Epidemiol 2008;168~~:~~(2):158-~~169~~69.

~~39.~~ 40 Moore~~, L.L.,~~ LL, Bradlee~~, M.L.,~~ ML, Singer~~, M.R.~~ MR, et al. BMI and waist circumference as predictors of lifetime colon cancer risk in Framingham Study adults. Int J Obes (Lond) 2004;28~~:~~(4):559-~~567~~67.

~~40.~~ 41 Bjorntorp~~,~~ P. "Portal" adipose tissue as a generator of risk factors for cardiovascular disease and diabetes. Arteriosclerosis 1990;10~~:~~(4):493-~~496.~~6.

~~41.~~ 42 Calle~~, E.E. &~~ EE, Kaaks~~,~~ R. Overweight, obesity and cancer: epidemiological evidence and proposed mechanisms. Nat Rev Cancer 2004;4~~:~~(8):579-~~591.~~91.

Formatted: Level 1

Formatted: Font: +Body (Calibri), 11 pt

For peer review only - http://bmjopen.bmj.com/site/about/guidelines.xhtml

For peer review only

For peer review only - http://bmjopen.bmj.com/site/about/guidelines.xhtml

Table legends

Formatted: Font: +Body (Calibri), 11 pt

Table 1A. Baseline characteristics and hazard ratios for BC of the identified factors (continuous variables).

Table 1B. Baseline characteristics and hazard ratios for BC of the identified factors (categorical variables).

Table 2. Correlation between skirt size (SS) at twenties, at follow-up (study-entry), change of SS (CSS) per 10 year and body mass index (BMI).

Table 3. Adjusted hazard ratios of BC according to all significant factorsConfounder-adjusted Cox model estimates for various adiposity measures and their association with breast cancer risk.

Formatted: None

Formatted: Font: +Body (Calibri), 11 pt

Formatted: Font: +Body (Calibri), 10 pt

Table 4. Relative hazards for a range of skirt size (SS) changes per 10 years (CSS) according to estimated multivariable model.

Supplementary tables legends

Supplementary Table S1A. Baseline characteristics of the identified factors (continuous variables) for group 1=decease in skirt size (SS) per 10 years; group 2=increase of 0 to 0.5 SS per 10 years; group 3=increase of 0.5 to 1 SS per 10 years; group 4=increase of 1+ SS per 10 years.

Supplementary Table S1B. Baseline characteristics of the identified factors (categorical variables) for group 1=decease in skirt size (SS) per 10 years; group 2=increase of 0 to 0.5 SS per 10 years; group 3=increase of 0.5 to 1 SS per 10 years; group 4=increase of 1+ SS per 10 years.

For peer review only - http://bmjopen.bmj.com/site/about/guidelines.xhtml

~~Supplementary Table S2. Hazard ratios for breast cancer (BC) adding body mass index (BMI) into the model.~~

~~Supplementary Table S3. Subsequent calculated hazard ratios for breast cancer (BC) by creating twenty completed datasets with multiple imputation.~~

Formatted: Font: +Body (Calibri), 11 pt

Formatted: Level 1

Figure legends

Figure 1. Relative hazards for a range of skirt size (SS) changes per 10 years (CSS) with 95% confidence bands.

Formatted: Level 1

Formatted: Font: +Body (Calibri), 11 pt, Bold

Figure 2. Distribution for skirt size (SS) at twenties, skirt size at current entry-study, BMI at recruitment and change of skirt size (CSS) every 10 years.

Formatted: Font: +Body (Calibri), 11 pt

Supplementary Figure Legends

Supplementary Figure S1. Plot of the scaled Schoenfeld residuals for change of skirt size (CSS) with smoother.

Formatted: Font: +Body (Calibri), 11 pt

Supplementary Figure S2. Estimated survival functions for 3 values of change of skirt SS (CSS) (CSS=0, CSS=1, CSS=0.~~21~~287 (median CSS)) with other covariate values set to their mean value. Solid lines represent estimated curves based on the Cox model, dashed lines represent estimated curves based on the exponential model.

Formatted: Font: +Body (Calibri), 11 pt

Formatted: Font: +Body (Calibri), 11 pt

~~**Supplementary figure legends**~~

Formatted: Font: +Body (Calibri), 11 pt, Not Bold

Formatted: Left, None, Space After: 0 pt, Line spacing: single

~~Supplementary Figure S1. Plot of the scaled Schoenfeld residuals for change of skirt size (CSS) with smoother.~~

Formatted: Font: +Body (Calibri), 11 pt

For peer review only

For peer review only - http://bmjopen.bmj.com/site/about/guidelines.xhtml

Supplementary Figure S2.

For peer review only - http://bmjopen.bmj.com/site/about/guidelines.xhtml

Tables ~~Cox-Snell residuals plotted against the estimated cumulative~~

Formatted: Font: +Body (Calibri), 11 pt, Bold

Table 1A. Baseline characteristics and hazard ~~of the~~ratios for breast cancer for adiposity measures and potential confounders (continuous variables).

Formatted: Font: +Body (Calibri), 11 pt

Factor	Mean	Standard deviation (SD)	25th percentile	50th percentile	75th percentile	Hazard Ratio (HR)	Lower 95%CI	Upper 95%CI	p-value
Skirt size at age 25 years	12.509	2.533	12	12	14	1.008	0.962	1.056	0.736
Skirt size at study-entry	15.133	3.452	12	14	16	1.045	**1.011**	**1.081**	**0.01**
CSS	0.334	0.359	0.191	0.287	0.533	1.283	**1.09**	**1.51**	**0.003**
BMI	26.449	4.783	23.203	25.598	28.821	1.061	**1.006**	**1.119**	**0.029**
IMD	0.011	1.01	-0.685	-0.327	0.458	1.008	0.952	1.068	0.778
Age at Last Period	48.975	6.009	46.264	49.977	52.703	1.024	**1.014**	**1.035**	**<0.001**
Age at First Period	12.959	1.607	12	13	14	1.02	0.983	1.06	0.293

CSS=Change of skirt size per 10 years
BMI=Body mass index at recruitment (5kg/m2)
CI=Confidence interval

For peer review only - http://bmjopen.bmj.com/site/about/guidelines.xhtml

Table 1B. Baseline characteristics and hazard ratios for breast cancer for potential confounders (categorical variables).

Categorical variable		All Women N (%)	Hazard ratio	Lower 95%CI	Upper 95%CI	p-value
Ethnicity	White	90,011 (97.35)	ref			
	Black	832 (0.90)	1.307	0.622	2.75	0.48
	Asian	490 (0.53)	0.827	0.443	1.541	0.549
	Chinese	166 (0.18)	0.554	0.078	3.936	0.555
	Other	669 (0.72)	0.693	0.288	1.668	0.413
Education	No O-Level	56,856 (61.24)	ref			
	O-Level	35,986 (38.76)	1.208	1.07	1.363	0.002
	No A-level	78,436 (84.48)	ref			
	A-Level	14,406 (15.52)	1.204	1.03	1.407	0.02
	No Clerical or commercial qualification	67,075 (72.25)	ref			
	Clerical or commercial qualification	25,767 (27.75)	1.085	0.953	1.236	0.219
	No Nursing or teaching	79,668 (85.81)	ref			
	Nursing or teaching	13,174 (14.19)	1.051	0.888	1.243	0.565
	No Degree college/University	74,640 (80.39)	ref			
	Degree college/University	18,202 (19.61)	1	0.859	1.163	0.996
	No other than above qualification	64,090 (69.03)	ref			
	Other than above qualification	28,752 (30.97)	0.828	0.724	0.947	0.006
Hysterectomy with ovarian conservation at recruitment	No	76,318 (82.20)	ref			
	Yes	16,524 (17.80)	0.961	0.821	1.125	0.621
Oral contraceptive pill use	No	37,083 (39.94)	ref			
	Yes	55,759 (60.06)	0.97	0.852	1.106	0.654
Hormone replacement therapy use at recruitment	No	73, 544 (79.21)	Ref			
	Yes	19,298 (20.79)	1.12	0.971	1.289	0.118
Hormone replacement therapy use at study-entry	No	85,866 (92.50)	ref			
	Yes	6,963 (7.50)	1.232	**1**	**1.517**	**0.049**
Sterilisation	No	74,550 (80.30)	ref			
	Yes	18,292 (19.70)	0.951	0.817	1.108	0.523
Infertility treatment	No	72,731 (78.34)	ref			
	Yes	20,111 (21.66)	1.459	**1.103**	**1.93**	**0.008**
Pregnancies less than 6 months	0	64,126 (69,90)	ref			
	1	18,896 (20.60)	1.124	0.971	1.302	0.116
	2	5,596 (6.10)	0.966	0.742	1.256	0.795

For peer review only

For peer review only - http://bmjopen.bmj.com/site/about/guidelines.xhtml

	3	1,790 (1.95)	1.521	**1.055**	**2.192**	**0.024**
	4	649 (0.71)	1.690	0.955	2.990	0.071
-	5	678 (0.74)	0.957	0.454	2.014	0.907
Pregnancies more than 6 months	0	10,986 (11.87)	ref			
	1	11,297 (12.18)	1.023	0.821	1.276	0.837
	2	40,890 (44.17)	0.768	**0.640**	**0.923**	**0.005**
	3	20,007 (21.61)	0.767	**0.624**	**0.944**	**0.012**
	4	6,612 (7.14)	0.824	0.626	1.084	0.625
-	5	2,804 (3.03)	0.594	0.386	0.915	0.385
Alcohol (units per week) at study entry	None	21,086 (22.97)				
	Less than 1	16,084 (17.52)	1.011	0.835	1.226	0.905
	1 to 3	18,717 (20.39)	1.062	0.886	1.275	0.511
	4 to 6	13,813 (15.05)	1.039	0.851	1.267	0.71
	7 to 10	11,056 (12.04)	0.949	0.762	1.181	0.64
	11 to 15	6,549 (7.13)	0.985	0.758	1.279	0.91
	16 to 30	2,990 (3.26)	1.271	0.92	1.745	0.146
	21+	1,501 (1.64)	1.331	0.866	2.044	0.192
Smoking	0	51,470 (56.27)	ref	-	-	-
	250	21,661 (23.68)	0.958	0.825	1.113	0.574
	500	9,632 (10.53)	1.032	0.847	1.259	0.752
-	750	8,706 (9.52)	1.153	0.947	1.403	0.156
Relatives Breast Cancer History	No	72, 731 (78.34)	ref			
-	Yes	20,111 (21.66)	1.48	**1.298**	**1.687**	**<0.0005**
Relatives Ovarian Cancer History	No	88,878 (95.73)	ref			
-	Yes	3,964 (4.27)	0.946	0.699	1.279	0.718

For peer review only - http://bmjopen.bmj.com/site/about/guidelines.xhtml

Table 2. Pearson correlation coefficients between skirt size at 25, and at study entry (follow-up questionnaire completion), change in skirt size (CSS) per 10 years and body mass index (BMI) at recruitment.

-	Skirt size at 25	Skirt size at study-entry	BMI at recruitment	CSS
Skirt size at 25	1.000	-	-	-
Skirt size at study-entry	0.606 (p<0.0005)	1.000		
BMI	0.330 (p<0.0005)	0.643 (p<0.0005)	1.000	
CSS	-0.153 (p<0.0005)	0.693 (P<0.0005)	0.497 (p<0.0005)	1.000

For peer review only - http://bmjopen.bmj.com/site/about/guidelines.xhtml

Table 3. Confounder-adjusted Cox model estimates for various adiposity measures and their association with breast cancer risk.

Measures of adiposity	Effective sample size	Number of events	Hazard ratio	Lower 95%CI	Upper 95%CI	p-value
full model with individual adiposity measure						
CSS	87334	1013	1.33	1.121	1.579	0.001
Skirt size at 25	87783	1017	1.006	0.958	1.056	0.829
Skirt size at study-entry	87661	1018	1.051	**1.014**	**1.089**	0.006
BMI at recruitment	87615	1021	1.076	**1.012**	**1.136**	0.009
full model with specific adiposity measure combinations						
CSS	86678	1008	1.266	1.041	1.538	0.018
BMI			1.037	0.97	1.109	0.287
Skirt size at study-entry	87003	1013	1.037	0.992	1.085	0.109
BMI			1.037	0.964	1.116	0.329
full model with one year time lag						
CSS	86284	650	1.422	**1.149**	**1.758**	0.001
full model with multiple imputation model						
CSS	92834	1090	1.3	1.102	1.534	0.002

CSS=Change of skirt size per 10 years
BMI=Body mass index at recruitment per 5kg/m2
CI=Confidence interval
Full model means adjusted for all potential confounders: Index of Multiple Deprivation(IMD), age at first and last period, number of pregnancies, hysterectomy, sterilisation, infertility, breast and ovarian cancer family history, oral contraceptive use (pill) use, hormone replacement therapy (HRT) use (from recruitment questionnaire); education, skirt size, continuing use of HRT, smoking, alcohol use, health status and cancer diagnosis (from study-entry questionnaire)

Formatted: Font: +Body (Calibri), 11 pt

Formatted: Justified

For peer review only - http://bmjopen.bmj.com/site/about/guidelines.xhtml

STROBE 2007 (v4) Statement—Checklist of items that should be included in reports of *cohort studies*

Section/Topic	Item #	Recommendation	Reported on page #
**Title and abstract**	1	(*a*) Indicate the study's design with a commonly used term in the title or the abstract	1-3
		(*b*) Provide in the abstract an informative and balanced summary of what was done and what was found	2-3
**Introduction**			
Background/rationale	2	Explain the scientific background and rationale for the investigation being reported	5-6
Objectives	3	State specific objectives, including any prespecified hypotheses	6
**Methods**			
Study design	4	Present key elements of study design early in the paper	7-8
Setting	5	Describe the setting, locations, and relevant dates, including periods of recruitment, exposure, follow-up, and data collection	7-8
Participants	6	(*a*) Give the eligibility criteria, and the sources and methods of selection of participants. Describe methods of follow-up	7-8
		(*b*) For matched studies, give matching criteria and number of exposed and unexposed	N/A
Variables	7	Clearly define all outcomes, exposures, predictors, potential confounders, and effect modifiers. Give diagnostic criteria, if applicable	7-9
Data sources/ measurement	8*	For each variable of interest, give sources of data and details of methods of assessment (measurement). Describe comparability of assessment methods if there is more than one group	*7-9*
Bias	9	Describe any efforts to address potential sources of bias	11
Study size	10	Explain how the study size was arrived at	7-9
Quantitative variables	11	Explain how quantitative variables were handled in the analyses. If applicable, describe which groupings were chosen and why	9-10
Statistical methods	12	(*a*) Describe all statistical methods, including those used to control for confounding	9
		(*b*) Describe any methods used to examine subgroups and interactions	N/A
		(*c*) Explain how missing data were addressed	10-11
		(*d*) If applicable, explain how loss to follow-up was addressed	8 and 10-11
		(*e*) Describe any sensitivity analyses	10-11
**Results**			

For peer review only

Participants	13*	(a) Report numbers of individuals at each stage of study—eg numbers potentially eligible, examined for eligibility, confirmed eligible, included in the study, completing follow-up, and analysed	12
		(b) Give reasons for non-participation at each stage	12
		(c) Consider use of a flow diagram	Not provided but described in the manuscript as stated above
Descriptive data	14*	(a) Give characteristics of study participants (eg demographic, clinical, social) and information on exposures and potential confounders	12
		(b) Indicate number of participants with missing data for each variable of interest	12-13 and 15
		(c) Summarise follow-up time (eg, average and total amount)	12
Outcome data	15*	Report numbers of outcome events or summary measures over time	12-13 and 15
Main results	16	(*a*) Give unadjusted estimates and, if applicable, confounder-adjusted estimates and their precision (eg, 95% confidence interval). Make clear which confounders were adjusted for and why they were included	13-14
		(*b*) Report category boundaries when continuous variables were categorized	N/A
		(*c*) If relevant, consider translating estimates of relative risk into absolute risk for a meaningful time period	15
Other analyses	17	Report other analyses done—eg analyses of subgroups and interactions, and sensitivity analyses	15
**Discussion**			
Key results	18	Summarise key results with reference to study objectives	19
**Limitations**			
Interpretation	20	Give a cautious overall interpretation of results considering objectives, limitations, multiplicity of analyses, results from similar studies, and other relevant evidence	16
Generalisability	21	Discuss the generalisability (external validity) of the study results	17-18
**Other information**			
Funding	22	Give the source of funding and the role of the funders for the present study and, if applicable, for the original study on which the present article is based	19

*Give information separately for cases and controls in case-control studies and, if applicable, for exposed and unexposed groups in cohort and cross-sectional studies.

For peer review only

Note: An Explanation and Elaboration article discusses each checklist item and gives methodological background and published examples of transparent reporting. The STROBE checklist is best used in conjunction with this article (freely available on the Web sites of PLoS Medicine at http://www.plosmedicine.org/, Annals of Internal Medicine at http://www.annals.org/, and Epidemiology at http://www.epidem.com/). Information on the STROBE Initiative is available at www.strobe-statement.org.

For peer review only

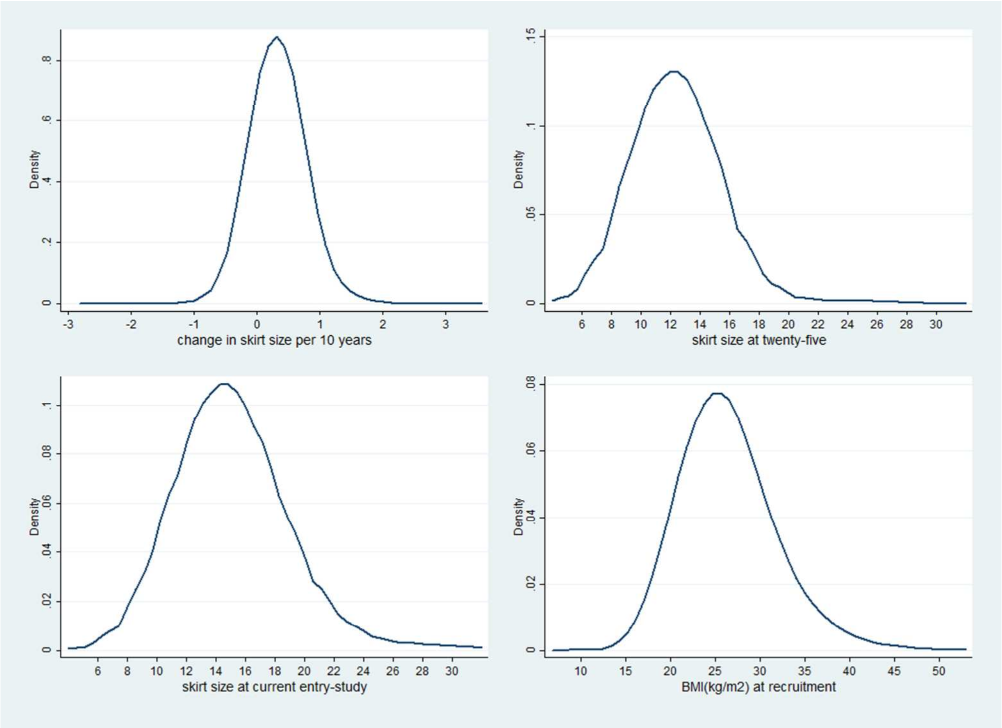

Relative hazards for a range of skirt size (SS) changes per 10 years (CSS) with 95% confidence bands.
370x269mm (72 x 72 DPI)

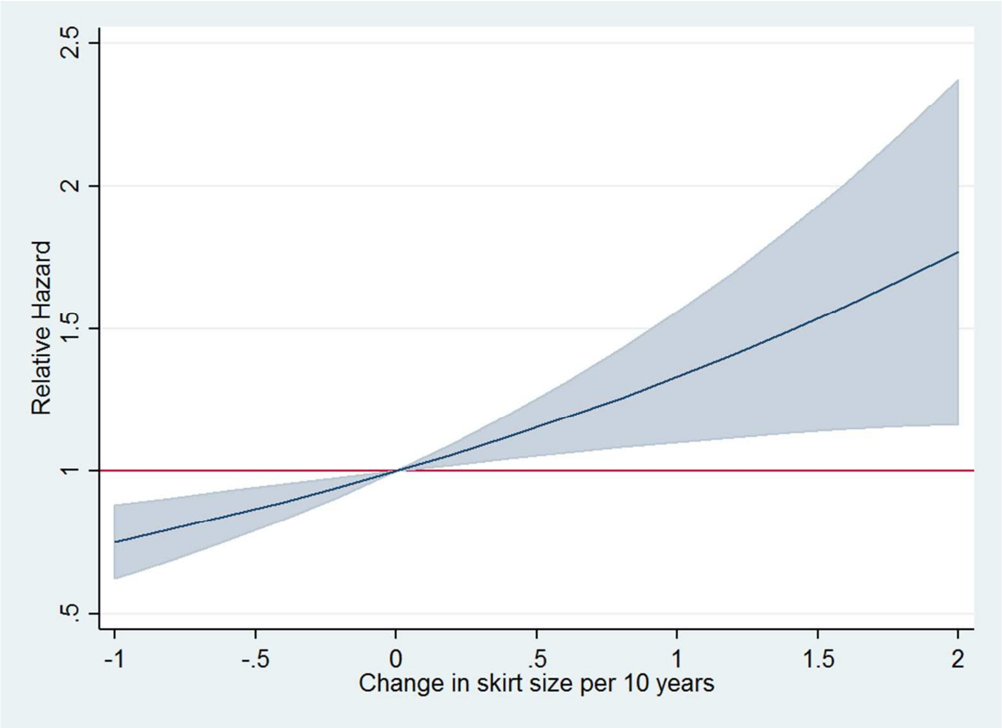

Distribution for skirt size (SS) at twenties, skirt size at current entry-study, BMI at recruitment and change of skirt size (CSS) every 10 years.
370x269mm (72 x 72 DPI)

For peer review only

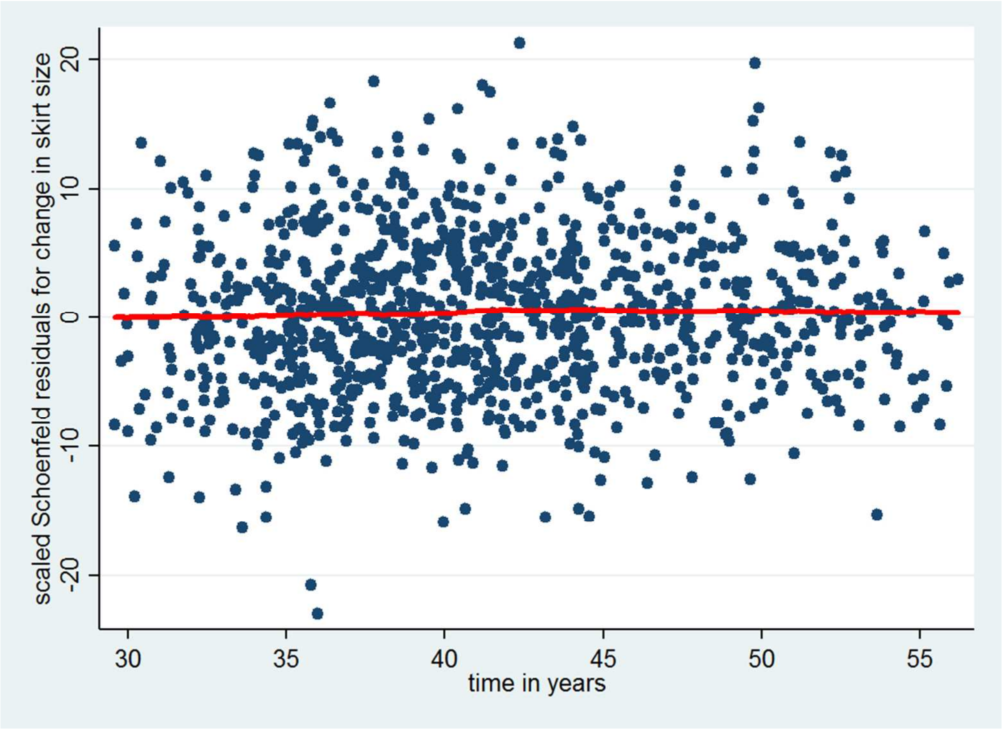

Plot of the scaled Schoenfeld residuals for change of skirt size (CSS) with smoother.
370x269mm (72 x 72 DPI)

For peer review only

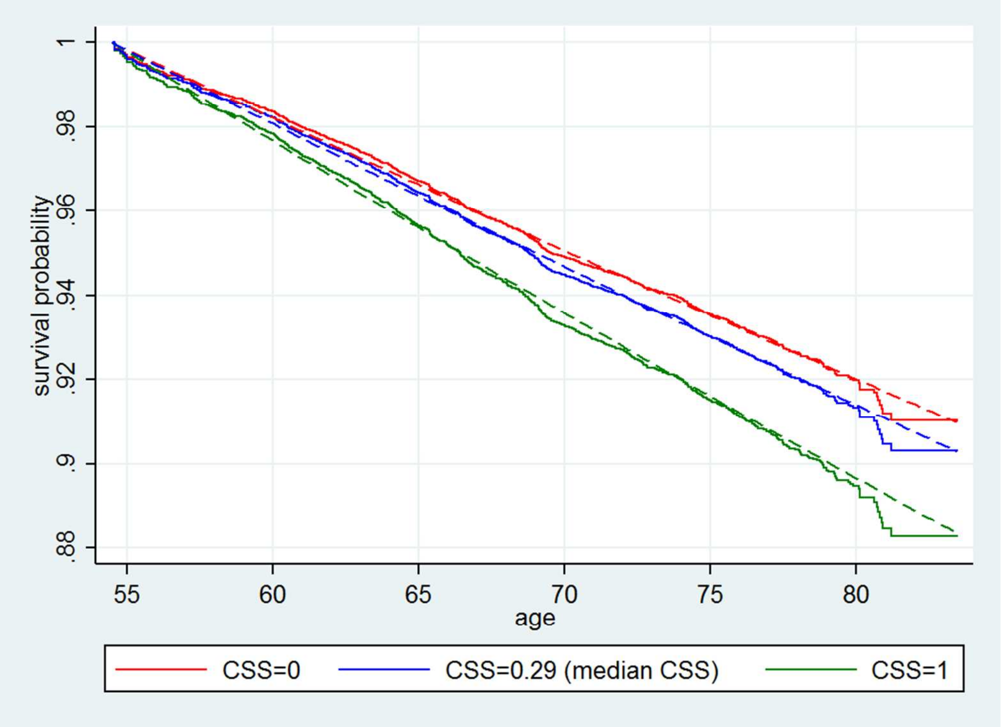

Estimated survival functions for 3 values of change of skirt SS (CSS) (CSS=0, CSS=1, CSS=0.287 (median CSS)) with other covariate values set to their mean value. Solid lines represent estimated curves based on the Cox model, dashed lines represent estimated curves based on the exponential model.
370x269mm (72 x 72 DPI)

BMJ Open

Association of skirt size and postmenopausal breast cancer risk in older women: a cohort study within the UK Collaborative Trial of Ovarian Cancer Screening (UKCTOCS)

Journal:	*BMJ Open*
Manuscript ID:	bmjopen-2014-005400.R2
Article Type:	Research
Date Submitted by the Author:	05-Aug-2014
Complete List of Authors:	Fourkala, Evangelia-Ourania; University College London, Women's Cancer<br>Burnell, Matthew; University College London, Women's Cancer<br>Cox, Catharine; University College London, Women's Cancer<br>Ryan, Andy; University College London, Women's Cancer<br>Salter, Laura; University College London Hospitals NHS Foundation Trust,<br>Gentry-Maharaj, Aleksandra; University College London, Women's Cancer<br>Parmar , Mahesh ; University College London, Hub for Trials Methodology<br>Jacobs, Ian; University of Manchester,<br>Menon, Usha; University College London, Women's Cancer
**Primary Subject Heading**:	Oncology
Secondary Subject Heading:	Epidemiology
Keywords:	ONCOLOGY, Breast tumours < ONCOLOGY, Epidemiology < ONCOLOGY

SCHOLARONE™ Manuscripts

For peer review only

Title: Association of skirt size and postmenopausal breast cancer risk in older women: a cohort study within the UK Collaborative Trial of Ovarian Cancer Screening (UKCTOCS)

Authors: Evangelia-Ourania Fourkala[1]*, Matthew Burnell[1]*, Catherine Cox[1], Andy Ryan[1], Laura Currin Salter[2], Aleksandra Gentry-Maharaj[1], Mahesh Parmar[3], Ian Jacobs[1, 4], Usha Menon[1**]

*joint

**corresponding author

Affiliations:

[1]Women's Cancer, Institute for Women's Health, University College London, London, UK

[2]University College London Hospitals NHS Foundation Trust, London, UK

[3]MRC Clinical Trials Unit, London, UK

[4]Academic Health Science Centre, University of Manchester, Manchester, UK

Corresponding author: Usha Menon, Gynaecological Cancer Research Centre, Women's Cancer, Institute for Women's Health, University College London, 149 Tottenham Road, London W1T 7DN, UK. u.menon@ucl.ac.uk

For peer review only - http://bmjopen.bmj.com/site/about/guidelines.xhtml

For peer review only

For peer review only - http://bmjopen.bmj.com/site/about/guidelines.xhtml

Abstract:

Objectives: Several studies suggest that overall and central-obesity are associated with increased breast cancer (BC) risk in postmenopausal-women. However, there are no studies investigating changes of central obesity and BC. We report on the association of BC risk with self-reported skirt size (SS) (waist-circumference proxy) changes between twenties and postmenopausal-age.

Design: Prospective cohort-study.

Setting: UK Collaborative Trial of Ovarian Cancer Screening (UKCTOCS) involving the nine trial centres in England.

Participants: Postmenopausal-women aged>50 with no known history of BC prior to or on the day of completion of the study-entry questionnaire.

Interventions: At recruitment and at study-entry, women were asked to complete a questionnaire. Women were followed-up via 'flagging' at the NHS Information Centre in England and the Hospital Episode Statistics.

Main outcome-measure: Time to initial BC diagnosis.

Results: Between 2nd January '05 and 1st July '10, 92834 UKCTOCS participants (median age 64.0) completed the study-entry questionnaire. During median follow-up of 3.19 years (25th-75thcentile: 2.46-3.78), 1090 women developed BC. Model adjusted analysis for potential confounders showed body mass index (BMI) at recruitment to UKCTOCS (Hazard Ratio (HR) for a 5 unit change=1.076, 95% CI: 1.012-1.136), current SS at study entry (HR=1.051; 95% CI: 1.014-1.089) and change in SS per 10-years (CSS) (HR=1.330; 95% CI: 1.121-1.579) were associated with increased BC risk but not SS at 25 (HR=1.006; 95% CI: 0.958-1.056). CSS was the most predictive singe adiposity measure and further analysis including both CSS and BMI in the model revealed CSS remained significant (HR=1.266; 95% CI: 1.041-1.538) but not BMI (HR=1.037; 95% CI: 0.970-1.109).

Conclusions: CSS is associated with BC risk independent of BMI. A unit increase in UK SS (e.g. 12 to

For peer review only - http://bmjopen.bmj.com/site/about/guidelines.xhtml

14) every 10-years between 25 and postmenopausal-age increases postmenopausal BC risk by 33%. Validation of these results could provide women with a simple and easy to understand message.

Strengths and limitations of this study:

- To the best of our knowledge, this is the first study investigating the association between central obesity using skirt size (SS) as a proxy and breast cancer risk. Between 25 and postmenopausal age, an increase in SS by one unit every decade increased the risk of postmenopausal breast cancer by 33% while decrease in SS was associated with lowering of risk.
- Our prospective cohort-study includes 94,000 women with comprehensive follow-up through data linkage to multiple national databases.
- There is a possibility of underestimation of self-reported SS. However, if current SS at study-entry is uniformly underestimated then there is merely rescaling of CSS so that the strength of the association is unaffected. Furthermore, recall bias of the SS at 25 maybe a limitation but unless this inability in reporting is systematically related to future breast cancer, measurement error can only result in underestimating the strength of the true association between CSS and breast cancer risk.
- Given that obesity is now emerging as a global epidemic, from a public health prospective these findings are significant as they provide women with a simple and easy to understand message.

Keywords: breast cancer, risk, skirt size, waist circumference, central obesity, BMI, postmenopausal

(Manuscript word count: 3 824; No of tables: 3; Number of figures: 2)

For peer review only - http://bmjopen.bmj.com/site/about/guidelines.xhtml

Introduction

Breast cancer is a major global health concern with 1 384 000 women being diagnosed every year [1]. Significant advances in breast cancer prevention have been accompanied by growing controversies regarding breast cancer screening. Both highlight the need for better risk stratification. During the last few years a growing number of studies have reported on anthropometric factors, particularly on the relationship between breast cancer risk and overall and/or central obesity [2-4].

A number of adiposity measures exist. The oldest and most common measure is body mass index (BMI) which is an empirical proxy for human body fat based on an individual's weight and height. Central fat is another measure of obesity. It is usually estimated by waist circumference, waist-hip ratio, waist-height ratio or the conicity index which evaluates waist circumference in relation to height and weight. An adequate and easy to use proxy measure for waist circumference is clothing (trouser/skirt) size, which has been shown to provide a reliable and feasible estimate of waist circumference at the population level [5]. It has been suggested that waist circumference independent of BMI, maybe predictive of increased health risk [6].

There is a large body of evidence that high BMI is associated with increased breast cancer risk in postmenopausal women and reduced risk in premenopausal women [3 4 7]. Another suggested breast cancer risk predictor is adult weight gain, which typically reflects an increase in body fat [8-10]. Results concerning weight loss are more equivocal; there are studies that show no significant effect upon risk, while some suggest postmenopausal weight loss may be protective [11-15]. Furthermore, the association of central obesity with breast cancer risk is unclear with studies reporting conflicting results [2 16]. Among postmenopausal women, several studies [17-24] but not all [14 25-29] have shown waist circumference or waist to hip ratio to be positively associated with increased breast cancer risk

For peer review only - http://bmjopen.bmj.com/site/about/guidelines.xhtml

[30]. There are, however, no studies examining changes in waist circumference or waist to hip ratio and breast cancer risk.

The UK Collaborative Trial of Ovarian Cancer Screening (UKCTOCS) [31], with comprehensive follow-up, provides the opportunity to examine the association of body size changes with breast cancer risk in a large prospective cohort. Furthermore, the availability of socioeconomic, reproductive, lifestyle and cancer family history data makes it possible to adjust for many potential confounders of breast risk.

Methods

Setting

UKCTOCS is a multicentre randomised controlled trial of ovarian cancer screening in the general population. The trial invited 1.2 million women of whom 202 638 were recruited between 17th April 2001 and 27th September 2005 through 13 trial centres in England, Wales and Northern Ireland. The detailed trial design and eligibility criteria have been described elsewhere [31]. All subjects completed a questionnaire at recruitment and a further postal follow-up questionnaire 3-4 years post-recruitment. The latter was the study-entry questionnaire for the current analysis. Follow-up was through a 'flagging study' with the Health and Social Care National Health Service (NHS) Information Centre who provided regular notifications of cancer registrations or deaths in the cohort. In addition, data linkage to the Hospital Episode Statistics (HES) an administrative database in England provided information on all inpatient and outpatient NHS hospital visits.

Women provided written consent for use of their data in secondary studies. Ethical approval was obtained from the Joint University College London (UCL)/UCL Hospital Committees on the Ethics of Human Research (REC reference:06/Q0505/102, June 2008).

For peer review only - http://bmjopen.bmj.com/site/about/guidelines.xhtml

Subjects

Participants recruited from England who returned the study-entry questionnaire prior to 2nd July 2010 (censorship date – see 'breast cancer notification'). Women recruited from Northern Ireland and Wales were excluded as HES was only available for women residing in England.

Data collection

Questionnaires

Recruitment questionnaire: This was completed at recruitment into UKCTOCS and included information on the variables listed in Table 1A and 1B. This included height and weight used to calculate BMI, the postcode which was used to derive the Index of Multiple Deprivation (IMD) (with lower scores indicating less deprived areas), age at first and last period, number of pregnancies, hysterectomy, sterilisation, infertility and breast and ovarian cancer family history, past oral contraceptive pill use, current hormone replacement therapy (HRT) use.

Study-entry questionnaire: This was completed at follow-up (3-4 years after recruitment) and provided information on education, skirt size, continuing use of HRT, smoking, alcohol use, health status and cancer diagnosis post recruitment (Table 1A and 1B). The date of completion of this questionnaire was the entry date for the current study. This was available in 89.8% of the women. Where this date was missing, the date of questionnaire data entry into the trial management system was used.

Breast cancer notification

The ICD-9 and ICD-10 codes used to identify women with breast cancer were 174* and C50*, respectively. Up-to-date data records from the NHS Information Centre and HES were searched to identify women with a breast cancer diagnosis. Women also self-reported breast cancer. Both at

For peer review only - http://bmjopen.bmj.com/site/about/guidelines.xhtml

For peer review only

recruitment and at current study-entry, women were asked specifically whether they had ever been diagnosed with breast cancer and if so, details of diagnosis date and details of treating physician [32]. Overall there was a good concordance between the different sources used to identify the breast cancer cases [32 33].

As there can be delays in cancer notification by the National Health and Social Care NHS Information Centre [32], the censorship date was set as 1stJuly 2010, two years prior to the date (10th July 2012) when data was provided by the NHS Information Centre agencies for this analysis. This ensured that as far as possible, all breast cancers in the stated period of observation were registered and therefore included. HES data was available for all the participants for the period between 6th of August 1998 when HES started and 31st March 2010.

Exposure variables

Self-reported height and weight were taken from the recruitment questionnaire and used to calculate BMI (with one unit equal here to 5kg/m^2). In the study-entry questionnaire two specific questions related to skirt size (SS) were asked: 1) 'What was your SS when you were in your twenties?' 2) 'What is your SS now?' Women could choose from 13 SS categories ranging from 6-30. The two SS questions were used to create a variable reflecting change in SS over time. The change in SS variable (CSS) was calculated as an increase in SS per 10 years. Note that a 'one unit' increase in SS means an increase by two nominal values, for example from size 10 to size 12 as odd sizes do not exist in the UK.

Statistical Analysis

Standard survival analysis methods were used to analyse the data. Cox models were preferred so that specification of an underlying hazard was not required. Women with breast cancer previous to

For peer review only - http://bmjopen.bmj.com/site/about/guidelines.xhtml

questionnaire completion date were excluded from the analysis. The date of study-entry questionnaire was the time-point used for entry into study. However, there was delayed entry (left truncation) in that they were only able to participate in the current study because they 'survived' (had not died, withdrawn from trial or got breast cancer) the period from their mid-twenties (the first skirt size question) to study-entry questionnaire completion date (the second current skirt size question). To account for a woman's contribution to the analysis being conditional on having 'survived' during the unobserved period, the length of which depends on the age at study entry, the timescale used was age rather than date. Therefore, the time origin was fixed at age=25 for all subjects and study entry was age at completion of study-entry questionnaire. Women were censored either at age at first breast cancer, death from any cause or 1st July 2010 whichever occurred first.

Summaries of all identified potential confounding risk factors were performed. These variables were also included individually in a Cox regression model to obtain univariable estimates of their hazard ratio relating to breast cancer risk. In addition kernel density plots of all four adiposity measures were created and Pearson correlation coefficients were calculated for all adiposity measure-pairings. To obtain an appropriate confounder-adjusted estimate of the relative hazard of breast cancer of increasing SS, a Cox regression model including CSS in continuous form with all potential risk factors, regardless of statistical significance, was fitted (the 'full model'). The functional form for CSS was determined using fractional polynomials, by considering powers from the standard predefined set {-2, -1, -0.5, 0, 0.5, 1, 2, 3}, and modelling the most appropriate relationship between CSS and the (log) relative hazard. The full model was refitted with each of the adiposity measures forming the exposure variable, in turn. In addition, a variant formulation of the full model included both CSS and BMI and both skirt size at current study-entry and BMI.

For peer review only - http://bmjopen.bmj.com/site/about/guidelines.xhtml

Cox-Snell residuals were used to test the model fit and the proportional hazards assumption used in Cox regression was tested using the scaled Schoenfeld residuals for each variable in the final model. The (undue) influence of any outliers was assessed by calculating the change in the parameter estimate when an observation is deleted. To visualise the 'dose-response' relationship model, predicted relative hazards with 95% confidence bands for a range of CSS values were plotted. To obtain estimates of absolute risk for certain scenarios (when CSS=0, CSS=1, CSS=median value), the event density function was assumed to be exponentially distributed, and the full model with CSS was fitted again using exponential regression. Survival curves with the same covariate pattern following both the Cox and exponential model fitting were plotted to ensure that the constant hazard was a reasonable assumption when averaged over the timeframe. An estimate of absolute risk was calculated by using the covariate-specific hazard from the exponential model for 1 year and for 5 years.

Sensitivity analyses

To guard against the possibility of reverse causality whereby preclinical breast cancer may result in weight gain, the full model with CSS was refitted but with a time-lag of one year so that the start of the study period occurs one year after completion of the study-entry questionnaire.

In the primary analysis, missing data in CSS or any other confounder was handled by case-wise deletion. To assess the robustness of estimation with such an approach to potential biases, multiple imputation (MI) was used to calculate the corresponding model estimates with missing data imputed appropriately. Specifically, a multivariate normal distribution formed by all potential predictors and the outcome variables (event and time) was used for the imputed random draws, and 20 fully imputed datasets were created with which to obtain valid estimates and standard errors.

For peer review only - http://bmjopen.bmj.com/site/about/guidelines.xhtml

Results

157 997 women residing in England were recruited to the trial between 17th April 2001 and 29th September 2005. 11 659 (9 440 withdrew, 533 moved away, 1 686 died) women were not sent the study-entry questionnaire. The remaining 146 338 women were sent the study-entry questionnaire between 28th June 2005 and 17th March 2009 and 112 945 (77.2%) responded by 31st December 2010. 10 689 of these women completed the current study-entry questionnaire after the censorship date of 1st July 2010 and were therefore excluded.

Eight women did not give consent for flagging. The remaining 102 249 were successfully flagged with the NHS Information Centre. Of these, 62 were lost to follow-up by the NHS Information Centre prior to questionnaire completion, 9 342 were diagnosed with breast cancer before or on the day of completion of the study-entry questionnaire and in 10, study-entry questionnaire completion date could not be calculated. The remaining 92 834 women were included in the study and were followed-up for a median of 3.19 years (25th-75th centile: 2.46-3.78 years). During the study period, 1 090 women developed breast cancer, resulting in an absolute risk of breast cancer of 1.2% (1 090 / 92 834) in this cohort of postmenopausal women, 75% of whom were aged over 60 years with no prior history of breast cancer. More information regarding the age distribution of the cohort is provided in supplementary material (Supplementary Figure S1).

The reproductive and lifestyle characteristics of the 92 834 women included in the analysis are summarised in Table 1A and B. Briefly, the women were mainly White, with a median year of birth at 1943 (Interquartile range (IQR): 1937-1947), 19.6% had a University degree and their median standardised - IMD (using 2008 data) was -0.327 (IQR: -0.685-0.458) indicating that most women are from less deprived areas. Most women were overweight (median BMI 25.598, (IQR: 23.203-28.821) at trial recruitment (median recruitment age 60.2; IQR: 55.8-65.8), had a median SS at 25 of 12 (IQR:

For peer review only

12-14) and at study-entry of 14 (IQR: 12-16) (median age 64.0; IQR: 59.7-69.7). The median CSS was 0.287 (IQR: 0.191-0.533) per 10 years, which is equivalent to an increase of 1 unit in SS (e.g. 10 to 12) in almost 35 years. Figure 1 shows the distribution for the four adiposity measures where CSS is largely symmetrical whilst the other three measures display some positive skewness.

The Pearson's correlation coefficients for the different adiposity measures are presented in Table 2. All of them were relatively high correlated apart from SS at 25 with CSS. Amongst the univariable Cox regressions, all measures of adiposity had significant hazard ratios (HRs) at the 5% level, except for SS at 25 (Table 1A and Table 1B). Other significant predictors of breast cancer risk were age at last period, number pregnancies greater than 6 months, infertility treatment, family history of breast cancer and HRT use at current study-entry (Table 1A and Table 1B).

5500 women were not included in the model due to missing values for either CSS or a confounder. The remaining 87 334 women, 1013 of whom developed breast cancer during follow-up were included in the full Cox model with CSS. Use of fractional polynomials of the CSS variable showed the most appropriate functional form was linear, with tests of better fit for other powers of degrees 1 (p=0.719) and 2 (p=0.981) showing no improvement. Confounder-adjustment revealed a stronger positive association between CSS and breast cancer risk (HR=1.330 per 10 years; 95%CI: 1.121-1.579; *p*=0.001) than without adjustment (Table 3). For each unit increase in SS per 10 years the hazard rate of breast cancer increased by 33%. Replacement of CSS with the other adiposity measures in a full model, again showed a significant but lesser association between BMI (HR=1.076 per 5 units; 95%CI: 1.018-1.136; *p*=0.009) and SS at study-entry (HR=1.051; 95%CI: 1.014-1.089; *p*=0.006) with breast cancer risk, but not for SS at 25 (HR=1.006; 95%CI: 0.958-1.056; *p*=0.809). Including recruitment BMI into the full model with CSS showed some sharing of predictive power but CSS had an association with breast cancer risk independent of BMI (HR=1.27; 95%CI: 1.041-1.538; *p*=0.018 for CSS; HR=1.04;

For peer review only - http://bmjopen.bmj.com/site/about/guidelines.xhtml

95%CI: 0.970-1.109; *p=0.287* for recruitment BMI) (Table 3). A full model with SS at study-entry (HR=1.037; 95% CI: 0.992-1.085; *p=0.109*) and BMI (HR=1.037; 95% CI: 0.964-1.116; *p=0.329*) suggested less independence between the 2 measures than between CSS and BMI and neither variable was statistically significant at α=0.05 (Table 3).

Tests and plots based on the Schoenfeld residuals showed no evidence of any violation of the proportional hazards assumption. Supplementary Figure S2 plots these residuals for CSS with a superimposed smoother and shows that the effect of CSS on breast cancer risk is essentially stable over the entire age range considered in this study. The Cox-Snell residuals plotted against their cumulative hazard showed a virtual line of unity indicating good model fit (figure not shown). Similar diagnostic results were found using the other adiposity measures.

Figure 2 shows how the model relates breast cancer risk to varying changes in SS. For example, for those with an increase of 2 SS every 10 years, the estimated relative hazard increases by 77% (HR=1.769; 95%CI: 1.164-2.375). Supplementary Figure S3 shows the estimated survival functions when CSS=0, CSS=1 and CSS=0.287 (the median CSS value), with other continuous covariates set to their mean value and categorical variables set to their base value, and starting at the earliest observed age entering the study. These functions are plotted for both the Cox and exponential model and show that the exponential model is a reasonable approximation over the timeframe considered here. The derived estimates of absolute risk for 1 year were 0.33% (95%CI: 0.30-0.36%) when CSS=0 and 0.43% (95%CI: 0.37-0.48%) when CSS=1 (exponential model HR=1.31). These estimates compare with 0.35% (95%CI: 0.33-0.38%) when CSS=0.287 (median CSS) and the raw incidence rate of 0.38% (1090 cancers in 3.07 mean study years). The 5 year absolute risks are 1.63% (95%CI: 1.48-1.77) when CSS=0 and 2.14% (95%CI: 1.87%-2.40%) when CSS=1 and a raw incidence rate (number of cancers per 5 years of study time across sample) of 1.91%.

For peer review only - http://bmjopen.bmj.com/site/about/guidelines.xhtml

A sensitivity analysis added a time-lag of one year to the primary analysis that used the full model with CSS. For this regression model only 86 284 women were included as 1 121 women were left-censored before one year, including 71 who had missing covariate data. The HR of 1.422 (95% CI: 1.149-1.758; p=0.001) is slightly larger compared to the primary analysis but not substantively different (Table 3).

A multiple imputation analysis was carried out to investigate the effect of missing data on the model estimation. Data were missing for one or more variables in 5.9% and for CSS in 1.2% of the sample. Twenty completed datasets were created using multiple imputation and the subsequent HR=1.300 (95% CI: 1.102-1.534; p=0.002) (Table 3) and standard error estimate for CSS was essentially unchanged compared to the case-wise model result.

Discussion

This is to date the only study that we are aware of investigating the association of change in SS and breast cancer risk. Our data suggests that a unit increase in UK SS (e.g. 12 to 14) every 10 years between the twenties and postmenopausal age increases the risk of postmenopausal breast cancer by 33%. We estimated an increase in 5-year absolute risk of postmenopausal breast cancer from 1 in 61 to 1 in 51 with each unit increase in SS per 10 years. Reductions in SS decreased the risk of breast cancer (Figure 2) though it should be noted that for 76% of the women in this study, SS increased over their adult lifetime. These findings may provide women with a simple and easy to understand message given that SS has been found to be a reliable measure for changes in waist circumference and one that women may relate and understand better in comparison to other adiposity measures such as BMI [5],

The major strengths of this study are its prospective cohort design, sample size, comprehensive follow-up through national registries and administrative databases and self-reporting resulting in high quality

For peer review only - http://bmjopen.bmj.com/site/about/guidelines.xhtml

data. Evaluation of numerous possible confounders including life style choices and socioeconomic status minimised bias. Due to missing data in the covariates, case-wise deletion meant 5508 women were dropped from the Cox regression model. However, a sensitivity analysis using 20 completed sets of multiply imputed data showed that the missing data had little effect on the overall outcome of the study. Furthermore, the study conclusions were maintained after introducing a time-lag of one year to the study entry to rule out reverse causality. The absolute risk of breast cancer in this cohort over a median follow-up of 3.2 years was 1.2% as only postmenopausal women with no prior history of breast cancer, 75% of whom were aged over 60 years, were included in this analysis. In keeping with other studies, family history of breast cancer, infertility, age at last period, HRT use were associated with increased breast cancer risk while pregnancies lasting over 6 months were protective [34]. Limitations of the study are the relatively short follow-up period, the possibility of underestimation of self-reported SS and recall bias of SS at 25. However, if current SS is uniformly underestimated across the sample then there is merely a rescaling of the CSS variable so that the strength of the relationship is unaffected, although the HR relating to a unit increase would be smaller. The same scenario applies to possible modern downsizing in SS to reflect increasing BMI (for example. a SS of 12 in their mid-twenties is now equivalent to, say, a SS of 10) assuming that this downsizing trend does not noticeably occur over the period of 'current study-entry SS'. All dates of FU questionnaire were between 2005 and 2010 with 97% between 2006 and 2009. The true relationship would only be weaker than reported if underestimation was not random but more prevalent amongst those who got breast cancer compared to those that did. Furthermore, unless the inability to accurately recall SS in the twenties is systematically related to future breast cancer or a confounder, measurement error can only result in underestimating the strength of the true relationship between CSS and risk of breast cancer.

The findings that CSS is associated with breast cancer risk (CSS per 10 years HR=1.33, 95%CI: 1.12-1.58; p=0.0010) and that addition of BMI to the model does not significantly improve risk prediction,

For peer review only - http://bmjopen.bmj.com/site/about/guidelines.xhtml

are consistent with previous studies addressing the relationship between body weight and breast cancer risk showing that adult weight gain is a better predictor of breast cancer risk than absolute weight or BMI [8-10 30]. Increases in waist circumference have also been reported to increase risk of other cancers such as pancreatic [35], endometrial [36] and ovarian [37] cancer. A more equivocal issue in the literature is whether weight loss has a protective effect. Our findings that a decrease in SS is associated with decreased risk are in line with the studies showing reduction of weight can reduce risk of postmenopausal breast cancer [11 12].

Current SS (at postmenopausal age) (HR=1.05, 95%CI: 1.01-1.09), but not SS in the twenties, was associated with a statistically significant increase in breast cancer risk and appears to be a better predictor compared to BMI. Our findings come in agreement with other studies [30] and in keeping with the findings of a comparison of adiposity measures in postmenopausal women where waist circumference was found to be a stronger risk factor than BMI for a variety of conditions [38]. Absolute waist circumference has also been associated with increased risk of other cancers such as lung [39] and colon [40].

In our study changes in SS was a better predictor of breast cancer risk compared to absolute SS in terms of strength of association. Although the exact mechanism of these relationships need to be better understood, there is a suggestion that body fat around the waist is more metabolically active than adipose tissue elsewhere [41]. Obesity is known to increase oestrogen levels as a result of aromatisation of androstenedione in adipose tissue as well as affect insulin resistance and chronic inflammation, well known factors shown to increase breast cancer risk [42].

Conclusion

For peer review only - http://bmjopen.bmj.com/site/about/guidelines.xhtml

Convincing evidence is available that high body weight, BMI and adult weight gain, which typically reflect an increase in body fat, are associated with increased breast cancer risk. To the best of our knowledge, this is the first study investigating the association of waist changes using skirt size as a proxy and breast cancer risk. Between twenties and postmenopausal age, an increase in skirt size by one unit every decade increases the risk of postmenopausal breast cancer by 33%. Validation of these results could provide women with a simple and easy to understand message.

For peer review only

For peer review only - http://bmjopen.bmj.com/site/about/guidelines.xhtml

Footnotes

Acknowledgements

The authors are particularly grateful to the women throughout the UK who are participating in the trial and to the entire medical, nursing and administrative staff who work on UKCTOCS.

Author's contributions

UM, EOF and MB designed the study. EOF, CC, LCS and UM undertook the literature research. EOF, MB and UM drafted the manuscript. MB and EOF prepared the figures and tables and MB undertook the statistical analysis. All authors were involved in data collection. EOF, CC and LCS were involved in data cleaning. All authors contributed to interpretation of the data and critically revised the manuscript and approved the final version.

Funding

UKCTOCS was core funded by the Medical Research Council, Cancer Research UK and National Institute of Health Research (NIHR) with additional support from the Eve Appeal and the NIHR Biomedical Research Centre at UCLH/UCL. The views expressed in the publication are those of the authors and not necessarily those of the funders. The funding source or the sponsor had no role in data collection, data analysis, data interpretation, or writing of the report. The researchers are independent from the funders.

Competing interests

UM and IJ have a financial interest through Abcodia Ltd in the third party exploitation of clinical trials biobanks, which have been developed through the research at UCL. During part of the trial IJ had a consultancy arrangement with Becton Dickinson in the field of tumour markers.

Ethical approval

For peer review only

The study was approved by the Joint University College London/University College London Hospital Committees on the Ethics of Human Research (REC reference: 06/Q0505/102, June 2008). Approvals for the use of hospital episode statistics data were obtained as part of the standard hospitals episode statistics approval process.

Patient Consent

Obtained

Provenance and peer review

Not commissioned; externally peer reviewed.

Data sharing statement

No additional data are available.

For peer review only

For peer review only - http://bmjopen.bmj.com/site/about/guidelines.xhtml

References:

1 http://globocan.iarc.fr/factsheet.asp.

2 Harvie M, Hooper L, Howell AH. Central obesity and breast cancer risk: a systematic review. Obes Rev 2003;4(3):157-73.

3 Friedenreich CM. Review of anthropometric factors and breast cancer risk. Eur J Cancer Prev 2001;10(1):15-32.

4 Wiseman M. The second World Cancer Research Fund/American Institute for Cancer Research expert report. Food, nutrition, physical activity, and the prevention of cancer: a global perspective. Proc Nutr Soc 2008;67(3):253-6.

5 Hughes LAE, Schouten LJ, Goldbohm RA, et al. Self-reported Clothing Size as a Proxy Measure for Body Size. Epidemiology 2009;20(5):673-76.

6 Battram DS, Beynon C, He M. The reliability and validity of using clothing size as a proxy for waist circumference measurement in adults. Applied physiology, nutrition, and metabolism = Physiologie appliquee, nutrition et metabolisme 2011;36(2):183-90.

7 Cheraghi Z, Poorolajal J, Hashem T, et al. Effect of body mass index on breast cancer during premenopausal and postmenopausal periods: a meta-analysis. PLoS One 2012;7(12):e51446.

8 Eliassen AH, Colditz GA, Rosner B, et al. Adult weight change and risk of postmenopausal breast cancer. Jama 2006;296(2):193-201.

9 Lahmann PH, Schulz M, Hoffmann K, et al. Long-term weight change and breast cancer risk: the European prospective investigation into cancer and nutrition (EPIC). Br J Cancer 2005;93(5):582-9.

10 Magnusson C, Baron J, Persson I, et al. Body size in different periods of life and breast cancer risk in post-menopausal women. Int J Cancer 1998;76(1):29-34.

For peer review only - http://bmjopen.bmj.com/site/about/guidelines.xhtml

11 Eliassen AH, Colditz GA, Rosner B, et al. Adult weight change and risk of postmenopausal breast cancer. JAMA 2006;296(2):193-201.

12 Parker ED, Folsom AR. Intentional weight loss and incidence of obesity-related cancers: the Iowa Women's Health Study. Int J Obes Relat Metab Disord 2003;27(12):1447-52.

13 Radimer KL, Ballard-Barbash R, Miller JS, et al. Weight change and the risk of late-onset breast cancer in the original Framingham cohort. Nutrition and cancer 2004;49(1):7-13.

14 Morimoto LM, White E, Chen Z, et al. Obesity, body size, and risk of postmenopausal breast cancer: the Women's Health Initiative (United States). Cancer Causes Control 2002;13(8):741-51.

15 Barnes-Josiah D, Potter JD, Sellers TA, et al. Early body size and subsequent weight gain as predictors of breast cancer incidence (Iowa, United States). Cancer Causes Control 1995;6(2):112-8.

16 Connolly BS, Barnett C, Vogt KN, et al. A meta-analysis of published literature on waist-to-hip ratio and risk of breast cancer. Nutr Cancer 2002;44(2):127-38.

17 Huang Z, Willett WC, Colditz GA, et al. Waist circumference, waist : hip ratio, and risk of breast cancer in the Nurses' Health Study. Am J Epidemiol 1999;150(12):1316-24.

18 Mannisto S, Pietinen P, Pyy M, et al. Body-size indicators and risk of breast cancer according to menopause and estrogen-receptor status. Int J Cancer 1996;68(1):8-13.

19 Ogundiran TO, Huo D, Adenipekun A, et al. Case-control study of body size and breast cancer risk in Nigerian women. Am J Epidemiol 2010;172(6):682-90.

20 Wu AH, Yu MC, Tseng CC, et al. Body size, hormone therapy and risk of breast cancer in Asian-American women. Int J Cancer 2007;120(4):844-52.

21 Kaaks R, Van Noord PA, Den Tonkelaar I, et al. Breast-cancer incidence in relation to height, weight and body-fat distribution in the Dutch "DOM" cohort. Int J Cancer 1998;76(5):647-51.

For peer review only

22 Nemesure B, Wu SY, Hennis A, et al. Body size and breast cancer in a black population--the Barbados National Cancer Study. Cancer Causes Control 2009;20(3):387-94.

23 Folsom AR, Kushi LH, Anderson KE, et al. Associations of general and abdominal obesity with multiple health outcomes in older women: the Iowa Women's Health Study. Arch Intern Med 2000;160(14):2117-28.

24 Fagherazzi G, Chabbert-Buffet N, Fabre A, et al. Hip circumference is associated with the risk of premenopausal ER-/PR- breast cancer. Int J Obes (Lond) 2012;36(3):431-9.

25 Muti P, Stanulla M, Micheli A, et al. Markers of insulin resistance and sex steroid hormone activity in relation to breast cancer risk: a prospective analysis of abdominal adiposity, sebum production, and hirsutism (Italy). Cancer Causes Control 2000;11(8):721-30.

26 Sonnenschein E, Toniolo P, Terry MB, et al. Body fat distribution and obesity in pre- and postmenopausal breast cancer. Int J Epidemiol 1999;28(6):1026-31.

27 Lahmann PH, Hoffmann K, Allen N, et al. Body size and breast cancer risk: findings from the European Prospective Investigation into Cancer And Nutrition (EPIC). Int J Cancer 2004;111(5):762-71.

28 den Tonkelaar I, Seidell JC, Collette HJ, et al. Obesity and subcutaneous fat patterning in relation to breast cancer in postmenopausal women participating in the Diagnostic Investigation of Mammary Cancer Project. Cancer 1992;69(11):2663-7.

29 Petrek JA, Peters M, Cirrincione C, et al. Is body fat topography a risk factor for breast cancer? Ann Intern Med 1993;118(5):356-62.

30 Pischon T, Nothlings U, Boeing H. Obesity and cancer. Proc Nutr Soc 2008;67(2):128-45.

For peer review only - http://bmjopen.bmj.com/site/about/guidelines.xhtml

31 Menon U, Gentry-Maharaj A, Ryan A, et al. Recruitment to multicentre trials--lessons from UKCTOCS: descriptive study. Bmj 2008;337:a2079.

32 Fourkala EO, Gentry-Maharaj A, Burnell M, et al. Histological confirmation of breast cancer registration and self-reporting in England and Wales: a cohort study within the UK Collaborative Trial of Ovarian Cancer Screening. Br J Cancer 2012;106(12):1910-6.

33 Gentry-Maharaj A, Fourkala EO, Burnell M, et al. Concordance of National Cancer Registration with self-reported breast, bowel and lung cancer in England and Wales: a prospective cohort study within the UK Collaborative Trial of Ovarian Cancer Screening. Br J Cancer 2013;109(11):2875-9.

34 Veronesi U, Boyle P, Goldhirsch A, et al. Breast cancer. Lancet 2005;365(9472):1727-41.

35 Aune D, Greenwood DC, Chan DS, et al. Body mass index, abdominal fatness and pancreatic cancer risk: a systematic review and non-linear dose-response meta-analysis of prospective studies. Ann Oncol 2012;23(4):843-52.

36 Yang TY, Cairns BJ, Allen N, et al. Postmenopausal endometrial cancer risk and body size in early life and middle age: prospective cohort study. Br J Cancer 2012;107(1):169-75.

37 Canchola AJ, Chang ET, Bernstein L, et al. Body size and the risk of ovarian cancer by hormone therapy use in the California Teachers Study cohort. Cancer Causes Control 2010;21(12):2241-8.

38 Hartz A, He T, Rimm A. Comparison of adiposity measures as risk factors in postmenopausal women. J Clin Endocrinol Metab 2012;97(1):227-33.

39 Kabat GC, Kim M, Hunt JR, et al. Body mass index and waist circumference in relation to lung cancer risk in the Women's Health Initiative. Am J Epidemiol 2008;168(2):158-69.

40 Moore LL, Bradlee ML, Singer MR, et al. BMI and waist circumference as predictors of lifetime colon cancer risk in Framingham Study adults. Int J Obes (Lond) 2004;28(4):559-67.

For peer review only

41 Bjorntorp P. "Portal" adipose tissue as a generator of risk factors for cardiovascular disease and diabetes. Arteriosclerosis 1990;10(4):493-6.

42 Calle EE, Kaaks R. Overweight, obesity and cancer: epidemiological evidence and proposed mechanisms. Nat Rev Cancer 2004;4(8):579-91.

For peer review only

For peer review only - http://bmjopen.bmj.com/site/about/guidelines.xhtml

Tables

Table 1A. Baseline characteristics and hazard ratios for breast cancer for adiposity measures and potential confounders (continuous variables).

Factor	Mean	Standard deviation (SD)	25th percentile	50th percentile	75th percentile	Hazard Ratio (HR)	Lower 95%CI	Upper 95%CI	p-value
Skirt size at age 25 years	12.509	2.533	12	12	14	1.008	0.962	1.056	0.736
Skirt size at study-entry	15.133	3.452	12	14	16	1.045	**1.011**	**1.081**	**0.01**
CSS	0.334	0.359	0.191	0.287	0.533	1.283	**1.09**	**1.51**	**0.003**
BMI	26.449	4.783	23.203	25.598	28.821	1.061	**1.006**	**1.119**	**0.029**
IMD	0.011	1.01	-0.685	-0.327	0.458	1.008	0.952	1.068	0.778
Age at Last Period	48.975	6.009	46.264	49.977	52.703	1.024	**1.014**	**1.035**	**<0.001**
Age at First Period	12.959	1.607	12	13	14	1.02	0.983	1.06	0.293

CSS=Change of skirt size per 10 years
BMI=Body mass index at recruitment (5kg/m2)
CI=Confidence interval

For peer review only

Table 1B. Baseline characteristics and hazard ratios for breast cancer for potential confounders (categorical variables).

Categorical variable		All Women N (%)	Hazard ratio	Lower 95%CI	Upper 95%CI	p-value
Ethnicity	White	90,011 (97.35)	ref			
	Black	832 (0.90)	1.307	0.622	2.75	0.48
	Asian	490 (0.53)	0.827	0.443	1.541	0.549
	Chinese	166 (0.18)	0.554	0.078	3.936	0.555
	Other	669 (0.72)	0.693	0.288	1.668	0.413
Education	No O-Level	56,856 (61.24)	ref			
	O-Level	35,986 (38.76)	1.208	1.07	1.363	0.002
	No A-level	78,436 (84.48)	ref			
	A-Level	14,406 (15.52)	1.204	1.03	1.407	0.02
	No Clerical or commercial qualification	67,075 (72.25)	ref			
	Clerical or commercial qualification	25,767 (27.75)	1.085	0.953	1.236	0.219
	No Nursing or teaching	79,668 (85.81)	ref			
	Nursing or teaching	13,174 (14.19)	1.051	0.888	1.243	0.565
	No Degree college/University	74,640 (80.39)	ref			
	Degree college/University	18,202 (19.61)	1	0.859	1.163	0.996
	No other than above qualification	64,090 (69.03)	ref			
	Other than above qualification	28,752 (30.97)	0.828	0.724	0.947	0.006

For peer review only - http://bmjopen.bmj.com/site/about/guidelines.xhtml

Hysterectomy with ovarian conservation at recruitment	No	76,318 (82.20)	ref			
	Yes	16,524 (17.80)	0.961	0.821	1.125	0.621
Oral contraceptive pill use	No	37,083 (39.94)	ref			
	Yes	55,759 (60.06)	0.97	0.852	1.106	0.654
Hormone replacement therapy use at recruitment	No	73, 544 (79.21)	Ref			
	Yes	19,298 (20.79)	1.12	0.971	1.289	0.118
Hormone replacement therapy use at study-entry	No	85,866 (92.50)	ref			
	Yes	6,963 (7.50)	1.232	**1**	**1.517**	**0.049**
Sterilisation	No	74,550 (80.30)	ref			
	Yes	18,292 (19.70)	0.951	0.817	1.108	0.523
Infertility treatment	No	72,731 (78.34)	ref			
	Yes	20,111 (21.66)	1.459	**1.103**	**1.93**	**0.008**
Pregnancies less than 6 months	0	64,126 (69,90)	ref			
	1	18,896 (20.60)	1.124	0.971	1.302	0.116
	2	5,596 (6.10)	0.966	0.742	1.256	0.795
	3	1,790 (1.95)	1.521	**1.055**	**2.192**	**0.024**
	4	649 (0.71)	1.690	0.955	2.990	0.071
	5	678 (0.74)	0.957	0.454	2.014	0.907
Pregnancies more than 6 months	0	10,986 (11.87)	ref			
	1	11,297 (12.18)	1.023	0.821	1.276	0.837
	2	40,890 (44.17)	0.768	**0.640**	**0.923**	**0.005**
	3	20,007 (21.61)	0.767	**0.624**	**0.944**	**0.012**
	4	6,612 (7.14)	0.824	0.626	1.084	0.625
	5	2,804 (3.03)	0.594	0.386	0.915	0.385
Alcohol (units per week) at study entry	None	21,086 (22.97)				
	Less than 1	16,084 (17.52)	1.011	0.835	1.226	0.905

For peer review only - http://bmjopen.bmj.com/site/about/guidelines.xhtml

	1 to 3	18,717 (20.39)	1.062	0.886	1.275	0.511
	4 to 6	13,813 (15.05)	1.039	0.851	1.267	0.71
	7 to 10	11,056 (12.04)	0.949	0.762	1.181	0.64
	11 to 15	6,549 (7.13)	0.985	0.758	1.279	0.91
	16 to 30	2,990 (3.26)	1.271	0.92	1.745	0.146
	21+	1,501 (1.64)	1.331	0.866	2.044	0.192
Smoking	0	51,470 (56.27)	ref			
	250	21,661 (23.68)	0.958	0.825	1.113	0.574
	500	9,632 (10.53)	1.032	0.847	1.259	0.752
	750	8,706 (9.52)	1.153	0.947	1.403	0.156
Relatives Breast Cancer History	No	72, 731 (78.34)	ref			
	Yes	20,111 (21.66)	1.48	**1.298**	**1.687**	**<0.0005**
Relatives Ovarian Cancer History	No	88,878 (95.73)	ref			
	Yes	3,964 (4.27)	0.946	0.699	1.279	0.718

For peer review only

For peer review only - http://bmjopen.bmj.com/site/about/guidelines.xhtml

Table 2. Pearson correlation coefficients between skirt size at 25, and at study entry (follow-up questionnaire completion), change in skirt size (CSS) per 10 years and body mass index (BMI) at recruitment.

	Skirt size at 25	Skirt size at study-entry	BMI at recruitment	CSS
Skirt size at 25	1.000			
Skirt size at study-entry	0.606 ($p<0.0005$)	1.000		
BMI	0.330 ($p<0.0005$)	0.643 ($p<0.0005$)	1.000	
CSS	-0.153 ($p<0.0005$)	0.693 ($P<0.0005$)	0.497 ($p<0.0005$)	1.000

For peer review only

Table 3. Confounder-adjusted Cox model estimates for various adiposity measures and their association with breast cancer risk.

Measures of adiposity	Effective sample size	Number of events	Hazard ratio	Lower 95%CI	Upper 95%CI	p-value
full model with individual adiposity measure						
CSS	87334	1013	1.33	1.121	1.579	0.001
Skirt size at 25	87783	1017	1.006	0.958	1.056	0.829
Skirt size at study-entry	87661	1018	1.051	**1.014**	**1.089**	0.006
BMI at recruitment	87615	1021	1.076	**1.012**	**1.136**	0.009
full model with specific adiposity measure combinations						
CSS	86678	1008	1.266	1.041	1.538	0.018
BMI			1.037	0.97	1.109	0.287
Skirt size at study-entry	87003	1013	1.037	0.992	1.085	0.109
BMI			1.037	0.964	1.116	0.329
full model with one year time lag						
CSS	86284	650	1.422	**1.149**	**1.758**	0.001
full model with multiple imputation model						
CSS	92834	1090	1.3	1.102	1.534	0.002

CSS=Change of skirt size per 10 years
BMI=Body mass index at recruitment per 5kg/m2
CI=Confidence interval
Full model means adjusted for all potential confounders: Index of Multiple Deprivation (IMD), age at first and last period, number of pregnancies, hysterectomy, sterilisation, infertility, breast and ovarian cancer family history, oral contraceptive use (pill) use, hormone replacement therapy (HRT) use (from recruitment questionnaire); education, skirt size, continuing use of HRT, smoking, alcohol use, health status and cancer diagnosis (from study-entry questionnaire)

For peer review only - http://bmjopen.bmj.com/site/about/guidelines.xhtml

Table legends

Table 1A. Baseline characteristics and hazard ratios for breast cancer of the identified factors (continuous variables).

Table 1B. Baseline characteristics and hazard ratios for breast cancer of the identified factors (categorical variables).

Table 2. Correlation between skirt size (SS) at twenties, at follow-up (study-entry), change of SS (CSS) per 10 year and body mass index (BMI).

Table 3. Confounder-adjusted Cox model estimates for various adiposity measures and their association with breast cancer risk.

Figure legends

Figure 1. Relative hazards for a range of skirt size (SS) changes per 10 years (CSS) with 95% confidence bands.

Figure 2. Distribution for skirt size (SS) at twenties, skirt size at current entry-study, BMI at recruitment and change of skirt size (CSS) every 10 years.

Supplementary Figure Legends

Supplementary Figure S1. Kernel density plot of age at study entry questionnaire.

Supplementary Figure S2. Plot of the scaled Schoenfeld residuals for change of skirt size (CSS) with smoother.

For peer review only

Supplementary Figure S3. Estimated survival functions for 3 values of change of skirt SS (CSS) (CSS=0, CSS=1, CSS=0.287 (median CSS)) with other covariate values set to their mean value. Solid lines represent estimated curves based on the Cox model, dashed lines represent estimated curves based on the exponential model.

For peer review only - http://bmjopen.bmj.com/site/about/guidelines.xhtml

Title: Association of skirt size and postmenopausal breast cancer risk in older women: a cohort study within the UK Collaborative Trial of Ovarian Cancer Screening (UKCTOCS)

Authors: Evangelia-Ourania Fourkala[1]*, Matthew Burnell[1]*, Catherine Cox[1], Andy Ryan[1], Laura Currin Salter[2], Aleksandra Gentry-Maharaj[1], Mahesh Parmar[3], Ian Jacobs[1, 4], Usha Menon[1**]

*joint

**corresponding author

Affiliations:

[1]Women's Cancer, Institute for Women's Health, University College London, London, UK

[2]University College London Hospitals NHS Foundation Trust, London, UK

[3]MRC Clinical Trials Unit, London, UK

[4]Academic Health Science Centre, University of Manchester, Manchester, UK

Corresponding author: Usha Menon, Gynaecological Cancer Research Centre, Women's Cancer, Institute for Women's Health, University College London, 149 Tottenham Road, London W1T 7DN, UK. u.menon@ucl.ac.uk

For peer review only

For peer review only - http://bmjopen.bmj.com/site/about/guidelines.xhtml

Abstract:

Objectives: Several studies suggest that overall and central-obesity are associated with increased breast cancer (BC) risk in postmenopausal-women. However, there are no studies investigating changes of central obesity and BC. We report on the association of BC risk with self-reported skirt size (SS) (waist-circumference proxy) changes between twenties and postmenopausal-age.

Design: Prospective cohort-study.

Setting: UK Collaborative Trial of Ovarian Cancer Screening (UKCTOCS) involving the nine trial centres in England.

Participants: Postmenopausal-women aged>50 with no known history of BC prior to or on the day of completion of the study-entry questionnaire.

Interventions: At recruitment and at study-entry, women were asked to complete a questionnaire. Women were followed-up via 'flagging' at the NHS Information Centre in England and the Hospital Episode Statistics.

Main outcome-measure: Time to initial BC diagnosis.

Results: Between 2nd January '05 and 1st July '10, 92834 UKCTOCS participants (median age 64.0) completed the study-entry questionnaire. During median follow-up of 3.19 years (25th-75thcentile: 2.46-3.78), 1090 women developed BC. ~~Univariable Cox models~~ Model adjusted analysis for potential confounders showed body mass index (BMI) at recruitment to UKCTOCS (Hazard Ratio (HR) for a 5 unit change=1.076, 95% CI: 1.012-1.136), current SS at study entry (HR=1.051; 95% CI: 1.014-1.089) and change in SS per 10-years (CSS) (HR=1.330; 95% CI: 1.121-1.579) were associated with increased BC risk but not SS at 25 (HR=1.006; 95% CI: 0.958-1.056). CSS was the most predictive singe adiposity measure and further analysis including both CSS and BMI in the model revealed CSS remained significant (HR=1.266; 95% CI: 1.041-1.538) but not BMI (HR=1.037; 95% CI: 0.970-1.109).

Conclusions: CSS is associated with BC risk independent of BMI. A unit increase in UK SS (e.g. 12 to

For peer review only - http://bmjopen.bmj.com/site/about/guidelines.xhtml

14) every 10-years between 25 and postmenopausal-age increases postmenopausal BC risk by 33%. Validation of these results could provide women with a simple and easy to understand message.

Formatted: Level 1, Space After: 10 pt, Adjust space between Latin and Asian text, Adjust space between Asian text and numbers

Strengths and limitations of this study:

- To the best of our knowledge, this is the first study investigating the association between central obesity using skirt size (SS) as a proxy and breast cancer ~~(BC)~~ risk. Between 25 and postmenopausal age, an increase in SS by one unit every decade increased the risk of postmenopausal breast cancer ~~BC~~ by 33% while decrease in SS was associated with lowering of risk.
- Our prospective cohort-study includes 94,000 women with comprehensive follow-up through data linkage to multiple national databases.
- There is a possibility of underestimation of self-reported SS. However, if current SS at study-entry is uniformly underestimated then there is merely rescaling of CSS so that the strength of the association is unaffected. Furthermore, recall bias of the SS at 25 maybe a limitation but unless this inability in reporting is systematically related to future breast cancer~~BC~~, measurement error can only result in underestimating the strength of the true association between CSS and breast cancer~~BC~~ risk.
- Given that obesity is now emerging as a global epidemic, from a public health prospective these findings are significant as they provide women with a simple and easy to understand message.

Keywords: breast cancer, risk, skirt size, waist circumference, central obesity, BMI, postmenopausal

(Manuscript word count: 3 824; No of tables: 3; Number of figures: 2)

For peer review only

For peer review only - http://bmjopen.bmj.com/site/about/guidelines.xhtml

For peer review only

4

Introduction

Breast cancer (BC) is a major global health concern with 1 384 000 women being diagnosed every year [1]. Significant advances in BC breast cancer prevention have been accompanied by growing controversies regarding BC breast cancer screening. Both highlight the need for better risk stratification. During the last few years a growing number of studies have reported on anthropometric factors, particularly on the relationship between breast cancerBC risk and overall and/or central obesity [2-4].

A number of adiposity measures exist. The oldest and most common measure is body mass index (BMI) which is an empirical proxy for human body fat based on an individual's weight and height. Central fat is another measure of obesity. It is usually estimated by waist circumference (WC), waist-hip ratio (WHR), waist-height ratio (WHtR) or the conicity index which evaluates waist circumferenceWC in relation to height and weight. An adequate and easy to use proxy measure for waist circumferenceWC is clothing (trouser/skirt) size, which has been shown to provide a reliable and feasible estimate of waist circumferenceWC at the population level [5]. It has been suggested that waist circumferenceWC independent of BMI, maybe predictive of increased health risk [6].

There is a large body of evidence that high BMI is associated with increased breast cancerBC risk in postmenopausal women and reduced risk in premenopausal women [3 4 7]. Another suggested breast cancerBC risk predictor is adult weight gain, which typically reflects an increase in body fat [8-10]. Results concerning weight loss are more equivocal; there are studies that show no significant effect upon risk, while some suggest postmenopausal weight loss may be protective [11-15]. Furthermore, the association of central obesity with breast cancerBC risk is unclear with studies reporting conflicting results [2 16]. Among postmenopausal women, several studies [17-24] but not all [14 25-29] have

For peer review only

shown waist circumferenceWC or WHR waist to hip ratio to be positively associated with increased breast cancerBC risk [30]. There are, however, no studies examining changes in waist circumferenceWC or waist to hip ratioWHR and breast cancerBC risk.

The UK Collaborative Trial of Ovarian Cancer Screening (UKCTOCS) [31], with comprehensive follow-up, provides the opportunity to examine the association of body size changes with breast cancerBC risk in a large prospective cohort. Furthermore, the availability of socioeconomic, reproductive, lifestyle and cancer family history data makes it possible to adjust for many potential confounders of breast risk.

Methods

Setting

UKCTOCS is a multicentre randomised controlled trial of ovarian cancer screening in the general population. The trial invited 1.2 million women of whom 202 638 were recruited between 17th April 2001 and 27th September 2005 through 13 trial centres in England, Wales and Northern Ireland. The detailed trial design and eligibility criteria have been described elsewhere [31]. All subjects completed a questionnaire at recruitment and a further postal follow-up questionnaire 3-4 years post-recruitment. The latter was the study-entry questionnaire for the current analysis. Follow-up was through a 'flagging study' with the Health and Social Care National Health Service (NHS) Information Centre who provided regular notifications of cancer registrations or deaths in the cohort. In addition, data linkage to the Hospital Episode Statistics (HES) an administrative database in England provided information on all inpatient and outpatient NHS hospital visits.

For peer review only - http://bmjopen.bmj.com/site/about/guidelines.xhtml

Women provided written consent for use of their data in secondary studies. Ethical approval was obtained from the Joint University College London (UCL)/UCL Hospital Committees on the Ethics of Human Research (REC reference:06/Q0505/102, June 2008).

Subjects

Participants recruited from England who returned the study-entry questionnaire prior to 2nd July 2010 (censorship date – see 'breast cancerBC notification'). Women recruited from Northern Ireland and Wales were excluded as HES was only available for women residing in England.

Data collection

Questionnaires

Recruitment questionnaire: This was completed at recruitment into UKCTOCS and included information on the variables listed in Table 1A and 1B. This included height and weight used to calculate BMI, the postcode which was used to derive the Index of Multiple Deprivation (IMD) (with lower scores indicating less deprived areas), age at first and last period, number of pregnancies, hysterectomy, sterilisation, infertility and breast and ovarian cancer family history, past oral contraceptive pill use, current hormone replacement therapy (HRT) use.

Study-entry questionnaire: This was completed at follow-up (3-4 years after recruitment) and provided information on education, skirt size, continuing use of HRT, smoking, alcohol use, health status and cancer diagnosis post recruitment (Table 1A and 1B). The date of completion of this questionnaire was the entry date for the current study. This was available in 89.8% of the women. Where this date was missing, the date of questionnaire data entry into the trial management system was used.

Breast cancerBC notification

For peer review only

The ICD-9 and ICD-10 codes used to identify women with breast cancerBC were 174* and C50*, respectively. Up-to-date data records from the NHS Information Centre and HES were searched to identify women with a breast cancerBC diagnosis. Women also self-reported breast cancerBC. Both at recruitment and at current study-entry, women were asked specifically whether they had ever been diagnosed with breast cancerBC and if so, details of diagnosis date and details of treating physician [32]. Overall there was a good concordance between the different sources used to identify the breast cancerBC cases [32 33].

As there can be delays in cancer notification by the National Health and Social Care NHS Information Centre [32], the censorship date was set as 1st July 2010, two years prior to the date (10th July 2012) when data was provided by the NHS Information Centre agencies for this analysis. This ensured that as far as possible, all breast cancersBCs in the stated period of observation were registered and therefore included. HES data was available for all the participants for the period between 6th of August 1998 when HES started and 31st March 2010.

Exposure variables

Self-reported height and weight were taken from the recruitment questionnaire and used to calculate BMI (with one unit equal here to 5kg/m^2). In the study-entry questionnaire two specific questions related to skirt size (SS) were asked: 1) 'What was your SS when you were in your twenties?' 2) 'What is your SS now?' Women could choose from 13 SS categories ranging from 6-30. The two SS questions were used to create a variable reflecting change in SS over time. The change in SS variable (CSS) was calculated as an increase in SS per 10 years. Note that a 'one unit' increase in SS means an increase by two nominal values, for example from size 10 to size 12 as odd sizes do not exist in the UK.

For peer review only - http://bmjopen.bmj.com/site/about/guidelines.xhtml

For peer review only

Statistical Analysis

Standard survival analysis methods were used to analyse the data. Cox models were preferred so that specification of an underlying hazard was not required. Women with breast cancer previous to questionnaire completion date were excluded from the analysis. The date of study-entry questionnaire was the time-point used for entry into study. However, there was delayed entry (left truncation) in that they were only able to participate in the current study because they 'survived' (had not died, withdrawn from trial or got breast cancer~~BC~~) the period from their mid-twenties (the first skirt size question) to study-entry questionnaire completion date (the second current skirt size question). To account for a woman's contribution to the analysis being conditional on having 'survived' during the unobserved period, the length of which depends on the age at study entry, the timescale used was age rather than date. Therefore, the time origin was fixed at age=25 for all subjects and study entry was age at completion of study-entry questionnaire. Women were censored either at age at first breast cancer~~BC~~, death from any cause or 1st July 2010 whichever occurred first.

Summaries of all identified potential confounding risk factors were performed. These variables were also included individually in a Cox regression model to obtain univariable estimates of their hazard ratio relating to breast cancer~~BC~~ risk. In addition kernel density plots of all four adiposity measures were created and Pearson correlation coefficients were calculated for all adiposity measure-pairings. To obtain an appropriate confounder-adjusted estimate of the relative hazard of breast cancer~~BC~~ of increasing SS, a Cox regression model including CSS in continuous form with all potential risk factors, regardless of statistical significance, was fitted (the 'full model'). The functional form for CSS was determined using fractional polynomials, by considering powers from the standard predefined set {-2, -1, -0.5, 0, 0.5, 1, 2, 3}, and modelling the most appropriate relationship between CSS and the (log) relative hazard. The full model was refitted with each of the adiposity measures forming the

For peer review only

For peer review only - http://bmjopen.bmj.com/site/about/guidelines.xhtml

exposure variable, in turn. In addition, a variant formulation of the full model included both CSS and BMI and both skirt size at current study-entry and BMI.

Cox-Snell residuals were used to test the model fit and the proportional hazards assumption used in Cox regression was tested using the scaled Schoenfeld residuals for each variable in the final model. The (undue) influence of any outliers was assessed by calculating the change in the parameter estimate when an observation is deleted. To visualise the 'dose-response' relationship model, predicted relative hazards with 95% confidence bands for a range of CSS values were plotted. To obtain estimates of absolute risk for certain scenarios (when CSS=0, CSS=1, CSS=median value), the event density function was assumed to be exponentially distributed, and the full model with CSS was fitted again using exponential regression. Survival curves with the same covariate pattern following both the Cox and exponential model fitting were plotted to ensure that the constant hazard was a reasonable assumption when averaged over the timeframe. An estimate of absolute risk was calculated by using the covariate-specific hazard from the exponential model for 1 year and for 5 years.

Sensitivity analyses

To guard against the possibility of reverse causality whereby preclinical breast cancer~~BC~~ may result in weight gain, the full model with CSS was refitted but with a time-lag of one year so that the start of the study period occurs one year after completion of the study-entry questionnaire.

In the primary analysis, missing data in CSS or any other confounder was handled by case-wise deletion. To assess the robustness of estimation with such an approach to potential biases, multiple imputation (MI) was used to calculate the corresponding model estimates with missing data imputed appropriately. Specifically, a multivariate normal distribution formed by all potential predictors and

For peer review only - http://bmjopen.bmj.com/site/about/guidelines.xhtml

the outcome variables (event and time) was used for the imputed random draws, and 20 fully imputed datasets were created with which to obtain valid estimates and standard errors.

Results

157 997 women residing in England were recruited to the trial between 17th April 2001 and 29th September 2005. 11 659 (9 440 withdrew, 533 moved away, 1 686 died) women were not sent the study-entry questionnaire. The remaining 146 338 women were sent the study-entry questionnaire between 28th June 2005 and 17th March 2009 and 112 945 (77.2%) responded by 31st December 2010. 10 689 of these women completed the current study-entry questionnaire after the censorship date of 1st July 2010 and were therefore excluded.

Eight women did not give consent for flagging. The remaining 102 249 were successfully flagged with the NHS Information Centre. Of these, 62 were lost to follow-up by the NHS Information Centre prior to questionnaire completion, 9 342 were diagnosed with breast cancerBC before or on the day of completion of the study-entry questionnaire and in 10, study-entry questionnaire completion date could not be calculated. The remaining 92 834 women were included in the study and were followed-up for a median of 3.19 years (25th-75th centile: 2.46-3.78 years). During the study period, 1 090 women developed breast cancerBC, resulting in an absolute risk of breast cancerBC of 1.2% (1 090 / 92 834) in this cohort of postmenopausal women, 75% of whom were aged over 60 years with no prior history of breast cancerBC. More information regarding the age distribution of the cohort is provided in supplementary material (Supplementary Figure S1).

The reproductive and lifestyle characteristics of the 92 834 women included in the analysis are summarised in Table 1A and B. Briefly, the women were mainly White, with a median year of birth at 1943 (Interquartile range (IQR): 1937-1947), 19.6% had a University degree and their median

For peer review only - http://bmjopen.bmj.com/site/about/guidelines.xhtml

standardised - IMD (using 2008 data) was -0.327 (IQR: -0.685-0.458) indicating that most women are from less deprived areas. Most women were overweight (median BMI 25.598, (IQR: 23.203-28.821) at trial recruitment (median recruitment age 60.2; IQR: 55.8-65.8), had a median SS at 25 of 12 (IQR: 12-14) and at study-entry of 14 (IQR: 12-16) (median age 64.0; IQR: 59.7-69.7). The median CSS was 0.287 (IQR: 0.191-0.533) per 10 years, which is equivalent to an increase of 1 unit in SS (e.g. 10 to 12) in almost 35 years. Figure 1 shows the distribution for the four adiposity measures where CSS is largely symmetrical whilst the other three measures display some positive skewness.

The Pearson's correlation coefficients for the different adiposity measures are presented in Table 2. All of them were relatively high correlated apart from SS at 25 with CSS. Amongst the univariable Cox regressions, all measures of adiposity had significant hazard ratios (HRs) at the 5% level, except for SS at 25 (Table 1A and Table 1B). Other significant predictors of breast cancerBC risk were age at last period, number pregnancies greater than 6 months, infertility treatment, family history of breast cancerBC and HRT use at current study-entry (Table 1A and Table 1B).

5500 women were not included in the model due to missing values for either CSS or a confounder. The remaining 87 334 women, 1013 of whom developed breast cancerBC during follow-up were included in the full Cox model with CSS. Use of fractional polynomials of the CSS variable showed the most appropriate functional form was linear, with tests of better fit for other powers of degrees 1 (p=0.719) and 2 (p=0.981) showing no improvement. Confounder-adjustment revealed a stronger positive association between CSS and breast cancerBC risk (HR=1.330 per 10 years; 95%CI: 1.121-1.579; *p*=0.001) than without adjustment (Table 3). For each unit increase in SS per 10 years the hazard rate of breast cancerBC increased by 33%. Replacement of CSS with the other adiposity measures in a full model, again showed a significant but lesser association between BMI (HR=1.076 per 5 units; 95%CI: 1.018-1.136; *p*=0.009) and SS at study-entry (HR=1.051; 95%CI: 1.014-1.089;

For peer review only - http://bmjopen.bmj.com/site/about/guidelines.xhtml

p=0.006) with breast cancerBC risk, but not for SS at 25 (HR=1.006; 95%CI: 0.958-1.056; p=0.809). Including recruitment BMI into the full model with CSS showed some sharing of predictive power but CSS had an association with breast cancerBC risk independent of BMI (HR=1.27; 95%CI: 1.041-1.538; p=0.018 for CSS; HR=1.04; 95%CI: 0.970-1.109; *p=0.287* for recruitment BMI) (Table 3). A full model with SS at study-entry (HR=1.037; 95% CI: 0.992-1.085; *p=0.109*) and BMI (HR=1.037; 95% CI: 0.964-1.116; *p=0.329*) suggested less independence between the 2 measures than between CSS and BMI and neither variable was statistically significant at α=0.05 (Table 3).

Tests and plots based on the Schoenfeld residuals showed no evidence of any violation of the proportional hazards assumption. Supplementary Figure S21 plots these residuals for CSS with a superimposed smoother and shows that the effect of CSS on breast cancerBC risk is essentially stable over the entire age range considered in this study. The Cox-Snell residuals plotted against their cumulative hazard showed a virtual line of unity indicating good model fit (figure not shown). Similar diagnostic results were found using the other adiposity measures.

Figure 2 shows how the model relates breast cancerBC risk to varying changes in SS. For example, for those with an increase of 2 SS every 10 years, the estimated relative hazard increases by 77% (HR=1.769; 95%CI: 1.164-2.375). Supplementary Figure S32 shows the estimated survival functions when CSS=0, CSS=1 and CSS=0.287 (the median CSS value), with other continuous covariates set to their mean value and categorical variables set to their base value, and starting at the earliest observed age entering the study. These functions are plotted for both the Cox and exponential model and show that the exponential model is a reasonable approximation over the timeframe considered here. The derived estimates of absolute risk for 1 year were 0.33% (95%CI: 0.30-0.36%) when CSS=0 and 0.43% (95%CI: 0.37-0.48%) when CSS=1 (exponential model HR=1.31). These estimates compare with 0.35% (95%CI: 0.33-0.38%) when CSS=0.287 (median CSS) and the raw

For peer review only - http://bmjopen.bmj.com/site/about/guidelines.xhtml

incidence rate of 0.38% (1090 cancers in 3.07 mean study years). The 5 year absolute risks are 1.63% (95%CI: 1.48-1.77) when CSS=0 and 2.14% (95%CI: 1.87%-2.40%) when CSS=1 and a raw incidence rate (number of cancers per 5 years of study time across sample) of 1.91%.

A sensitivity analysis added a time-lag of one year to the primary analysis that used the full model with CSS. For this regression model only 86 284 women were included as 1 121 women were left-censored before one year, including 71 who had missing covariate data. The HR of 1.422 (95% CI: 1.149-1.758; *p*=0.001) is slightly larger compared to the primary analysis but not substantively different (Table 3).

A multiple imputation analysis was carried out to investigate the effect of missing data on the model estimation. Data were missing for one or more variables in 5.9% and for CSS in 1.2% of the sample. Twenty completed datasets were created using multiple imputation and the subsequent HR=1.300 (95% CI: 1.102-1.534; *p*=0.002) (Table 3) and standard error estimate for CSS was essentially unchanged compared to the case-wise model result.

Discussion

This is to date the only study that we are aware of investigating the association of change in SS and ~~BC~~ breast cancer risk. Our data suggests that a unit increase in UK SS (e.g. 12 to 14) every 10 years between the twenties and postmenopausal age increases the risk of postmenopausal breast cancer~~BC~~ by 33%. We estimated an increase in 5-year absolute risk of postmenopausal breast cancer~~BC~~ from 1 in 61 to 1 in 51 with each unit increase in SS per 10 years. Reductions in SS decreased the risk of breast cancer~~BC~~ (Figure 2) though it should be noted that for 76% of the women in this study, SS increased over their adult lifetime. These findings may provide women with a simple and easy to understand message given that SS has been found to be a reliable measure for changes in waist circumference~~WC~~

For peer review only

and one that women may relate and understand better in comparison to other adiposity measures such as BMI [5],

The major strengths of this study are its prospective cohort design, sample size, comprehensive follow-up through national registries and administrative databases and self-reporting resulting in high quality data. Evaluation of numerous possible confounders including life style choices and socioeconomic status minimised bias. Due to missing data in the covariates, case-wise deletion meant 5508 women were dropped from the Cox regression model. However, a sensitivity analysis using 20 completed sets of multiply imputed data showed that the missing data had little effect on the overall outcome of the study. Furthermore, the study conclusions were maintained after introducing a time-lag of one year to the study entry to rule out reverse causality. The absolute risk of breast cancer~~BC~~ in this cohort over a median follow-up of 3.2 years was 1.2% as only postmenopausal women with no prior history of breast cancer~~BC~~, 75% of whom were aged over 60 years, were included in this analysis. In keeping with other studies, family history of breast cancer~~BC~~, infertility, age at last period, HRT use were associated with increased breast cancer~~BC~~ risk while pregnancies lasting over 6 months were protective [34]. Limitations of the study are the relatively short follow-up period, the possibility of underestimation of self-reported SS and recall bias of SS at 25. However, if current SS is uniformly underestimated across the sample then there is merely a rescaling of the CSS variable so that the strength of the relationship is unaffected, although the HR relating to a unit increase would be smaller. The same scenario applies to possible modern downsizing in SS to reflect increasing BMI (for example. a SS of 12 in their mid-twenties is now equivalent to, say, a SS of 10) assuming that this downsizing trend does not noticeably occur over the period of 'current study-entry SS'. All dates of FU questionnaire were between 2005 and 2010 with 97% between 2006 and 2009. The true relationship would only be weaker than reported if underestimation was not random but more prevalent amongst those who got breast cancer~~BC~~ compared to those that did. Furthermore, unless the inability to accurately recall SS in the twenties is

For peer review only

systematically related to future breast cancer~~BC~~ or a confounder, measurement error can only result in underestimating the strength of the true relationship between CSS and risk of breast cancer~~BC~~.

The findings that CSS is associated with breast cancer~~BC~~ risk (CSS per 10 years HR=1.33, 95%CI: 1.12-1.58; *p*=0.0010) and that addition of BMI to the model does not significantly improve risk prediction, are consistent with previous studies addressing the relationship between body weight and ~~B~~breast cancer~~C~~ risk showing that adult weight gain is a better predictor of breast cancer~~BC~~ risk than absolute weight or BMI [8-10 30]. Increases in waist circumference~~WC~~ have also been reported to increase risk of other cancers such as pancreatic [35], endometrial [36] and ovarian [37] cancer. A more equivocal issue in the literature is whether weight loss has a protective effect. Our findings that a decrease in SS is associated with decreased risk are in line with the studies showing reduction of weight can reduce risk of postmenopausal breast cancer~~BC~~ [11 12].

Current SS (at postmenopausal age) (HR=1.05, 95%CI: 1.01-1.09), but not SS in the twenties, was associated with a statistically significant increase in breast cancer~~BC~~ risk and appears to be a better predictor compared to BMI. Our findings come in agreement with other studies [30] and in keeping with the findings of a comparison of adiposity measures in postmenopausal women where waist circumference~~WC~~ was found to be a stronger risk factor than BMI for a variety of conditions [38]. Absolute waist circumference~~WC~~ has also been associated with increased risk of other cancers such as lung [39] and colon [40].

In our study changes in SS was a better predictor of breast cancer~~BC~~ risk compared to absolute SS in terms of strength of association. Although the exact mechanism of these relationships need to be better understood, there is a suggestion that body fat around the waist is more metabolically active than adipose tissue elsewhere [41]. Obesity is known to increase oestrogen levels as a result of

For peer review only

For peer review only - http://bmjopen.bmj.com/site/about/guidelines.xhtml

aromatisation of androstenedione in adipose tissue as well as affect insulin resistance and chronic inflammation, well known factors shown to increase breast cancer~~BC~~ risk [42].

Conclusion

Convincing evidence is available that high body weight, BMI and adult weight gain, which typically reflect an increase in body fat, are associated with increased breast cancer~~BC~~ risk. To the best of our knowledge, this is the first study investigating the association of waist changes using skirt size as a proxy and breast cancer~~BC~~ risk. Between twenties and postmenopausal age, an increase in skirt size by one unit every decade increases the risk of postmenopausal breast cancer~~BC~~ by 33%. Validation of these results could provide women with a simple and easy to understand message.

Acknowledgements

The authors are particularly grateful to the women throughout the UK who are participating in the trial and to the entire medical, nursing and administrative staff who work on UKCTOCS.

Footnotes

Author's contributions

UM, EOF and MB designed the study. EOF, CC, LCS and UM undertook the literature research. EOF, MB and UM drafted the manuscript. MB and EOF prepared the figures and tables and MB undertook the statistical analysis. All authors were involved in data collection. EOF, CC and LCS were involved in data cleaning. All authors contributed to interpretation of the data and critically revised the manuscript and approved the final version.

Funding

For peer review only

UKCTOCS was core funded by the Medical Research Council, Cancer Research UK and National Institute of Health Research (NIHR) with additional support from the Eve Appeal and the NIHR Biomedical Research Centre at UCLH/UCL. The views expressed in the publication are those of the authors and not necessarily those of the funders. The funding source or the sponsor had no role in data collection, data analysis, data interpretation, or writing of the report. The researchers are independent from the funders.

Competing interests

UM and IJ have a financial interest through Abcodia Ltd in the third party exploitation of clinical trials biobanks, which have been developed through the research at UCL. During part of the trial IJ had a consultancy arrangement with Becton Dickinson in the field of tumour markers.

Ethical approval

The study was approved by the Joint University College London/University College London Hospital Committees on the Ethics of Human Research (REC reference: 06/Q0505/102, June 2008). Approvals for the use of hospital episode statistics data were obtained as part of the standard hospitals episode statistics approval process.

Patient Consent

Obtained

Provenance and peer review

Not commissioned; externally peer reviewed.

Data sharing statement

No additional data are available.

For peer review only

For peer review only - http://bmjopen.bmj.com/site/about/guidelines.xhtml

19

References:

1 http://globocan.iarc.fr/factsheet.asp.

2 Harvie M, Hooper L, Howell AH. Central obesity and breast cancer risk: a systematic review. Obes Rev 2003;4(3):157-73.

3 Friedenreich CM. Review of anthropometric factors and breast cancer risk. Eur J Cancer Prev 2001;10(1):15-32.

4 Wiseman M. The second World Cancer Research Fund/American Institute for Cancer Research expert report. Food, nutrition, physical activity, and the prevention of cancer: a global perspective. Proc Nutr Soc 2008;67(3):253-6.

5 Hughes LAE, Schouten LJ, Goldbohm RA, et al. Self-reported Clothing Size as a Proxy Measure for Body Size. Epidemiology 2009;20(5):673-76.

6 Battram DS, Beynon C, He M. The reliability and validity of using clothing size as a proxy for waist circumference measurement in adults. Applied physiology, nutrition, and metabolism = Physiologie appliquee, nutrition et metabolisme 2011;36(2):183-90.

7 Cheraghi Z, Poorolajal J, Hashem T, et al. Effect of body mass index on breast cancer during premenopausal and postmenopausal periods: a meta-analysis. PLoS One 2012;7(12):e51446.

8 Eliassen AH, Colditz GA, Rosner B, et al. Adult weight change and risk of postmenopausal breast cancer. Jama 2006;296(2):193-201.

9 Lahmann PH, Schulz M, Hoffmann K, et al. Long-term weight change and breast cancer risk: the European prospective investigation into cancer and nutrition (EPIC). Br J Cancer 2005;93(5):582-9.

10 Magnusson C, Baron J, Persson I, et al. Body size in different periods of life and breast cancer risk in post-menopausal women. Int J Cancer 1998;76(1):29-34.

For peer review only

For peer review only - http://bmjopen.bmj.com/site/about/guidelines.xhtml

11 Eliassen AH, Colditz GA, Rosner B, et al. Adult weight change and risk of postmenopausal breast cancer. JAMA 2006;296(2):193-201.

12 Parker ED, Folsom AR. Intentional weight loss and incidence of obesity-related cancers: the Iowa Women's Health Study. Int J Obes Relat Metab Disord 2003;27(12):1447-52.

13 Radimer KL, Ballard-Barbash R, Miller JS, et al. Weight change and the risk of late-onset breast cancer in the original Framingham cohort. Nutrition and cancer 2004;49(1):7-13.

14 Morimoto LM, White E, Chen Z, et al. Obesity, body size, and risk of postmenopausal breast cancer: the Women's Health Initiative (United States). Cancer Causes Control 2002;13(8):741-51.

15 Barnes-Josiah D, Potter JD, Sellers TA, et al. Early body size and subsequent weight gain as predictors of breast cancer incidence (Iowa, United States). Cancer Causes Control 1995;6(2):112-8.

16 Connolly BS, Barnett C, Vogt KN, et al. A meta-analysis of published literature on waist-to-hip ratio and risk of breast cancer. Nutr Cancer 2002;44(2):127-38.

17 Huang Z, Willett WC, Colditz GA, et al. Waist circumference, waist : hip ratio, and risk of breast cancer in the Nurses' Health Study. Am J Epidemiol 1999;150(12):1316-24.

18 Mannisto S, Pietinen P, Pyy M, et al. Body-size indicators and risk of breast cancer according to menopause and estrogen-receptor status. Int J Cancer 1996;68(1):8-13.

19 Ogundiran TO, Huo D, Adenipekun A, et al. Case-control study of body size and breast cancer risk in Nigerian women. Am J Epidemiol 2010;172(6):682-90.

20 Wu AH, Yu MC, Tseng CC, et al. Body size, hormone therapy and risk of breast cancer in Asian-American women. Int J Cancer 2007;120(4):844-52.

21 Kaaks R, Van Noord PA, Den Tonkelaar I, et al. Breast-cancer incidence in relation to height, weight and body-fat distribution in the Dutch "DOM" cohort. Int J Cancer 1998;76(5):647-51.

For peer review only

For peer review only - http://bmjopen.bmj.com/site/about/guidelines.xhtml

22 Nemesure B, Wu SY, Hennis A, et al. Body size and breast cancer in a black population--the Barbados National Cancer Study. Cancer Causes Control 2009;20(3):387-94.

23 Folsom AR, Kushi LH, Anderson KE, et al. Associations of general and abdominal obesity with multiple health outcomes in older women: the Iowa Women's Health Study. Arch Intern Med 2000;160(14):2117-28.

24 Fagherazzi G, Chabbert-Buffet N, Fabre A, et al. Hip circumference is associated with the risk of premenopausal ER-/PR- breast cancer. Int J Obes (Lond) 2012;36(3):431-9.

25 Muti P, Stanulla M, Micheli A, et al. Markers of insulin resistance and sex steroid hormone activity in relation to breast cancer risk: a prospective analysis of abdominal adiposity, sebum production, and hirsutism (Italy). Cancer Causes Control 2000;11(8):721-30.

26 Sonnenschein E, Toniolo P, Terry MB, et al. Body fat distribution and obesity in pre- and postmenopausal breast cancer. Int J Epidemiol 1999;28(6):1026-31.

27 Lahmann PH, Hoffmann K, Allen N, et al. Body size and breast cancer risk: findings from the European Prospective Investigation into Cancer And Nutrition (EPIC). Int J Cancer 2004;111(5):762-71.

28 den Tonkelaar I, Seidell JC, Collette HJ, et al. Obesity and subcutaneous fat patterning in relation to breast cancer in postmenopausal women participating in the Diagnostic Investigation of Mammary Cancer Project. Cancer 1992;69(11):2663-7.

29 Petrek JA, Peters M, Cirrincione C, et al. Is body fat topography a risk factor for breast cancer? Ann Intern Med 1993;118(5):356-62.

30 Pischon T, Nothlings U, Boeing H. Obesity and cancer. Proc Nutr Soc 2008;67(2):128-45.

For peer review only - http://bmjopen.bmj.com/site/about/guidelines.xhtml

31 Menon U, Gentry-Maharaj A, Ryan A, et al. Recruitment to multicentre trials--lessons from UKCTOCS: descriptive study. Bmj 2008;337:a2079.

32 Fourkala EO, Gentry-Maharaj A, Burnell M, et al. Histological confirmation of breast cancer registration and self-reporting in England and Wales: a cohort study within the UK Collaborative Trial of Ovarian Cancer Screening. Br J Cancer 2012;106(12):1910-6.

33 Gentry-Maharaj A, Fourkala EO, Burnell M, et al. Concordance of National Cancer Registration with self-reported breast, bowel and lung cancer in England and Wales: a prospective cohort study within the UK Collaborative Trial of Ovarian Cancer Screening. Br J Cancer 2013;109(11):2875-9.

34 Veronesi U, Boyle P, Goldhirsch A, et al. Breast cancer. Lancet 2005;365(9472):1727-41.

35 Aune D, Greenwood DC, Chan DS, et al. Body mass index, abdominal fatness and pancreatic cancer risk: a systematic review and non-linear dose-response meta-analysis of prospective studies. Ann Oncol 2012;23(4):843-52.

36 Yang TY, Cairns BJ, Allen N, et al. Postmenopausal endometrial cancer risk and body size in early life and middle age: prospective cohort study. Br J Cancer 2012;107(1):169-75.

37 Canchola AJ, Chang ET, Bernstein L, et al. Body size and the risk of ovarian cancer by hormone therapy use in the California Teachers Study cohort. Cancer Causes Control 2010;21(12):2241-8.

38 Hartz A, He T, Rimm A. Comparison of adiposity measures as risk factors in postmenopausal women. J Clin Endocrinol Metab 2012;97(1):227-33.

39 Kabat GC, Kim M, Hunt JR, et al. Body mass index and waist circumference in relation to lung cancer risk in the Women's Health Initiative. Am J Epidemiol 2008;168(2):158-69.

40 Moore LL, Bradlee ML, Singer MR, et al. BMI and waist circumference as predictors of lifetime colon cancer risk in Framingham Study adults. Int J Obes (Lond) 2004;28(4):559-67.

For peer review only

41 Bjorntorp P. "Portal" adipose tissue as a generator of risk factors for cardiovascular disease and diabetes. Arteriosclerosis 1990;10(4):493-6.

42 Calle EE, Kaaks R. Overweight, obesity and cancer: epidemiological evidence and proposed mechanisms. Nat Rev Cancer 2004;4(8):579-91.

For peer review only

For peer review only - http://bmjopen.bmj.com/site/about/guidelines.xhtml

Tables

Table 1A. Baseline characteristics and hazard ratios for breast cancer for adiposity measures and potential confounders (continuous variables).

Factor	Mean	Standard deviation (SD)	25th percentile	50th percentile	75th percentile	Hazard Ratio (HR)	Lower 95%CI	Upper 95%CI	p-value
Skirt size at age 25 years	12.509	2.533	12	12	14	1.008	0.962	1.056	0.736
Skirt size at study-entry	15.133	3.452	12	14	16	1.045	**1.011**	**1.081**	**0.01**
CSS	0.334	0.359	0.191	0.287	0.533	1.283	**1.09**	**1.51**	**0.003**
BMI	26.449	4.783	23.203	25.598	28.821	1.061	**1.006**	**1.119**	**0.029**
IMD	0.011	1.01	-0.685	-0.327	0.458	1.008	0.952	1.068	0.778
Age at Last Period	48.975	6.009	46.264	49.977	52.703	1.024	**1.014**	**1.035**	**<0.001**
Age at First Period	12.959	1.607	12	13	14	1.02	0.983	1.06	0.293

CSS=Change of skirt size per 10 years
BMI=Body mass index at recruitment (5kg/m2)
CI=Confidence interval

For peer review only

Table 1B. Baseline characteristics and hazard ratios for breast cancer for potential confounders (categorical variables).

Categorical variable		All Women N (%)	Hazard ratio	Lower 95%CI	Upper 95%CI	p-value
Ethnicity	White	90,011 (97.35)	ref			
	Black	832 (0.90)	1.307	0.622	2.75	0.48
	Asian	490 (0.53)	0.827	0.443	1.541	0.549
	Chinese	166 (0.18)	0.554	0.078	3.936	0.555
	Other	669 (0.72)	0.693	0.288	1.668	0.413
Education	No O-Level	56,856 (61.24)	ref			
	O-Level	35,986 (38.76)	1.208	1.07	1.363	0.002
	No A-level	78,436 (84.48)	ref			
	A-Level	14,406 (15.52)	1.204	1.03	1.407	0.02
	No Clerical or commercial qualification	67,075 (72.25)	ref			
	Clerical or commercial qualification	25,767 (27.75)	1.085	0.953	1.236	0.219
	No Nursing or teaching	79,668 (85.81)	ref			
	Nursing or teaching	13,174 (14.19)	1.051	0.888	1.243	0.565
	No Degree college/University	74,640 (80.39)	ref			
	Degree college/University	18,202 (19.61)	1	0.859	1.163	0.996
	No other than above qualification	64,090 (69.03)	ref			
	Other than above qualification	28,752 (30.97)	0.828	0.724	0.947	0.006

For peer review only

For peer review only - http://bmjopen.bmj.com/site/about/guidelines.xhtml

Hysterectomy with ovarian conservation at recruitment	No	76,318 (82.20)	ref			
	Yes	16,524 (17.80)	0.961	0.821	1.125	0.621
Oral contraceptive pill use	No	37,083 (39.94)	ref			
	Yes	55,759 (60.06)	0.97	0.852	1.106	0.654
Hormone replacement therapy use at recruitment	No	73, 544 (79.21)	Ref			
	Yes	19,298 (20.79)	1.12	0.971	1.289	0.118
Hormone replacement therapy use at study-entry	No	85,866 (92.50)	ref			
	Yes	6,963 (7.50)	1.232	**1**	**1.517**	**0.049**
Sterilisation	No	74,550 (80.30)	ref			
	Yes	18,292 (19.70)	0.951	0.817	1.108	0.523
Infertility treatment	No	72,731 (78.34)	ref			
	Yes	20,111 (21.66)	1.459	**1.103**	**1.93**	**0.008**
Pregnancies less than 6 months	0	64,126 (69,90)	ref			
	1	18,896 (20.60)	1.124	0.971	1.302	0.116
	2	5,596 (6.10)	0.966	0.742	1.256	0.795
	3	1,790 (1.95)	1.521	**1.055**	**2.192**	**0.024**
	4	649 (0.71)	1.690	0.955	2.990	0.071
	5	678 (0.74)	0.957	0.454	2.014	0.907
Pregnancies more than 6 months	0	10,986 (11.87)	ref			
	1	11,297 (12.18)	1.023	0.821	1.276	0.837
	2	40,890 (44.17)	0.768	**0.640**	**0.923**	**0.005**
	3	20,007 (21.61)	0.767	**0.624**	**0.944**	**0.012**
	4	6,612 (7.14)	0.824	0.626	1.084	0.625
	5	2,804 (3.03)	0.594	0.386	0.915	0.385
Alcohol (units per week) at study entry	None	21,086 (22.97)				
	Less than 1	16,084 (17.52)	1.011	0.835	1.226	0.905

For peer review only - http://bmjopen.bmj.com/site/about/guidelines.xhtml

	1 to 3	18,717 (20.39)	1.062	0.886	1.275	0.511
	4 to 6	13,813 (15.05)	1.039	0.851	1.267	0.71
	7 to 10	11,056 (12.04)	0.949	0.762	1.181	0.64
	11 to 15	6,549 (7.13)	0.985	0.758	1.279	0.91
	16 to 30	2,990 (3.26)	1.271	0.92	1.745	0.146
	21+	1,501 (1.64)	1.331	0.866	2.044	0.192
Smoking	0	51,470 (56.27)	ref			
	250	21,661 (23.68)	0.958	0.825	1.113	0.574
	500	9,632 (10.53)	1.032	0.847	1.259	0.752
	750	8,706 (9.52)	1.153	0.947	1.403	0.156
Relatives Breast Cancer History	No	72, 731 (78.34)	ref			
	Yes	20,111 (21.66)	1.48	**1.298**	**1.687**	**<0.0005**
Relatives Ovarian Cancer History	No	88,878 (95.73)	ref			
	Yes	3,964 (4.27)	0.946	0.699	1.279	0.718

For peer review only - http://bmjopen.bmj.com/site/about/guidelines.xhtml

Table 2. Pearson correlation coefficients between skirt size at 25, and at study entry (follow-up questionnaire completion), change in skirt size (CSS) per 10 years and body mass index (BMI) at recruitment.

	Skirt size at 25	Skirt size at study-entry	BMI at recruitment	CSS
Skirt size at 25	1.000			
Skirt size at study-entry	0.606 (p<0.0005)	1.000		
BMI	0.330 (p<0.0005)	0.643 (p<0.0005)	1.000	
CSS	-0.153 (p<0.0005)	0.693 (P<0.0005)	0.497 (p<0.0005)	1.000

For peer review only

For peer review only - http://bmjopen.bmj.com/site/about/guidelines.xhtml

Table 3. Confounder-adjusted Cox model estimates for various adiposity measures and their association with breast cancer risk.

Measures of adiposity	Effective sample size	Number of events	Hazard ratio	Lower 95%CI	Upper 95%CI	p-value
full model with individual adiposity measure						
CSS	87334	1013	1.33	1.121	1.579	0.001
Skirt size at 25	87783	1017	1.006	0.958	1.056	0.829
Skirt size at study-entry	87661	1018	1.051	**1.014**	**1.089**	0.006
BMI at recruitment	87615	1021	1.076	**1.012**	**1.136**	0.009
full model with specific adiposity measure combinations						
CSS	86678	1008	1.266	1.041	1.538	0.018
BMI			1.037	0.97	1.109	0.287
Skirt size at study-entry	87003	1013	1.037	0.992	1.085	0.109
BMI			1.037	0.964	1.116	0.329
full model with one year time lag						
CSS	86284	650	1.422	**1.149**	**1.758**	0.001
full model with multiple imputation model						
CSS	92834	1090	1.3	1.102	1.534	0.002

CSS=Change of skirt size per 10 years
BMI=Body mass index at recruitment per 5kg/m2
CI=Confidence interval
Full model means adjusted for all potential confounders: Index of Multiple Deprivation (IMD), age at first and last period, number of pregnancies, hysterectomy, sterilisation, infertility, breast and ovarian cancer family history, oral contraceptive use (pill) use, hormone replacement therapy (HRT) use (from recruitment questionnaire); education, skirt size, continuing use of HRT, smoking, alcohol use, health status and cancer diagnosis (from study-entry questionnaire)

For peer review only - http://bmjopen.bmj.com/site/about/guidelines.xhtml

Table legends

Table 1A. Baseline characteristics and hazard ratios for breast cancerBC of the identified factors (continuous variables).

Table 1B. Baseline characteristics and hazard ratios for breast cancerBC of the identified factors (categorical variables).

Table 2. Correlation between skirt size (SS) at twenties, at follow-up (study-entry), change of SS (CSS) per 10 year and body mass index (BMI).

Table 3. Confounder-adjusted Cox model estimates for various adiposity measures and their association with breast cancer risk.

Figure legends

Figure 1. Relative hazards for a range of skirt size (SS) changes per 10 years (CSS) with 95% confidence bands.

Figure 2. Distribution for skirt size (SS) at twenties, skirt size at current entry-study, BMI at recruitment and change of skirt size (CSS) every 10 years.

Supplementary Figure Legends

Supplementary Figure S1. Kernel density plot of age at study entry questionnaire.

Supplementary Figure S21. Plot of the scaled Schoenfeld residuals for change of skirt size (CSS) with smoother.

For peer review only

For peer review only - http://bmjopen.bmj.com/site/about/guidelines.xhtml

Supplementary Figure S32. Estimated survival functions for 3 values of change of skirt SS (CSS) (CSS=0, CSS=1, CSS=0.287 (median CSS)) with other covariate values set to their mean value. Solid lines represent estimated curves based on the Cox model, dashed lines represent estimated curves based on the exponential model.

For peer review only

For peer review only - http://bmjopen.bmj.com/site/about/guidelines.xhtml

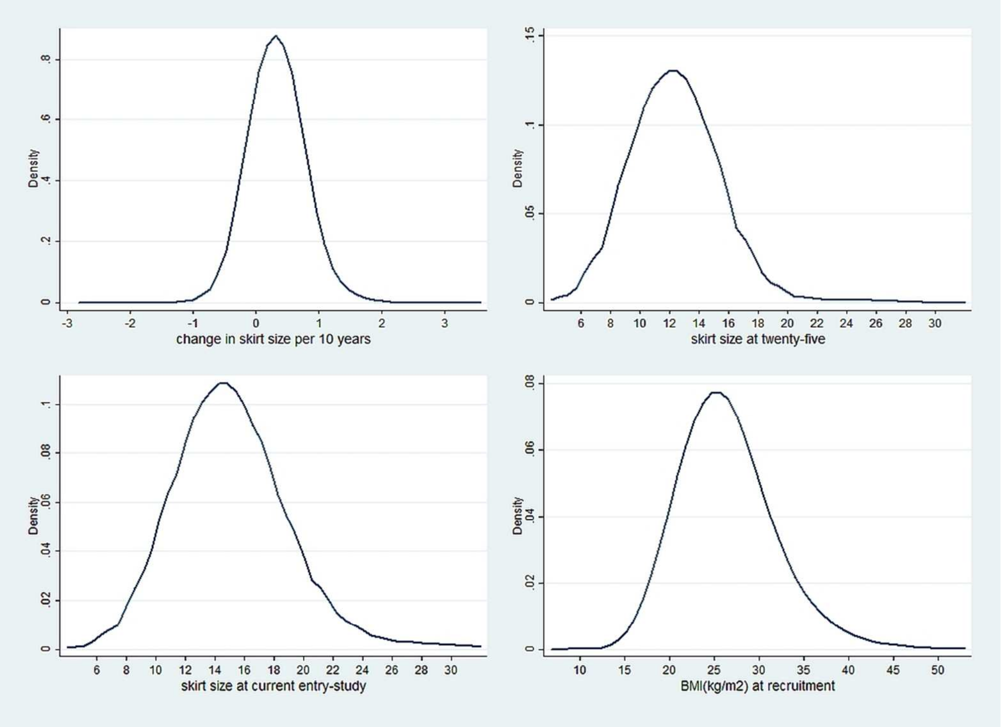

Relative hazards for a range of skirt size (SS) changes per 10 years (CSS) with 95% confidence bands.
90x65mm (300 x 300 DPI)

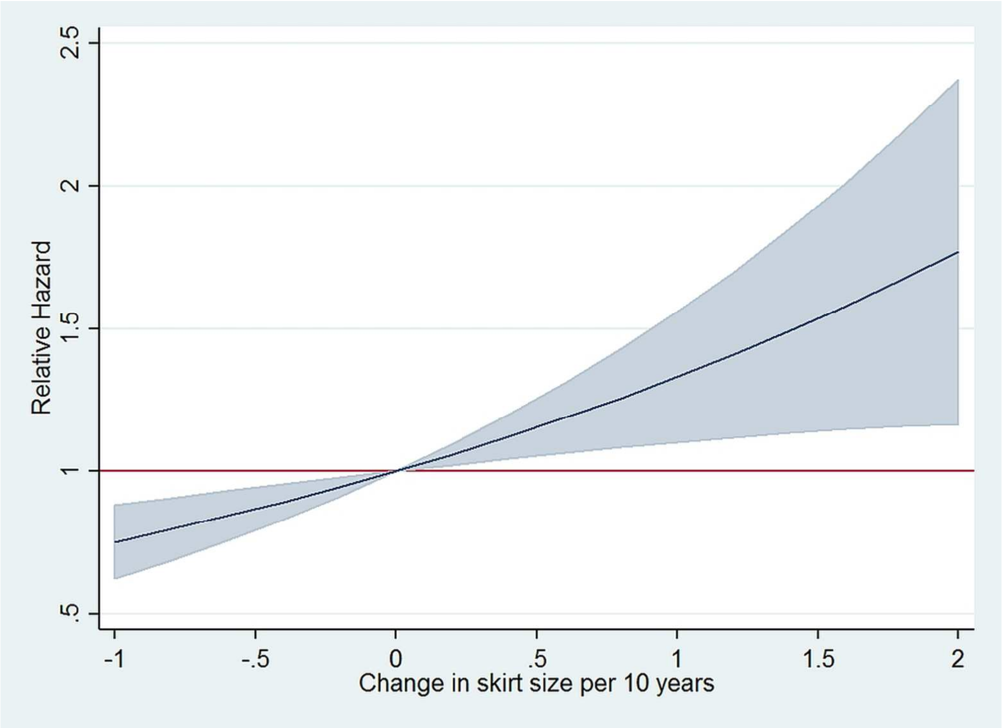

Distribution for skirt size (SS) at twenties, skirt size at current entry-study, BMI at recruitment and change of skirt size (CSS) every 10 years.
90x65mm (300 x 300 DPI)

For peer review only

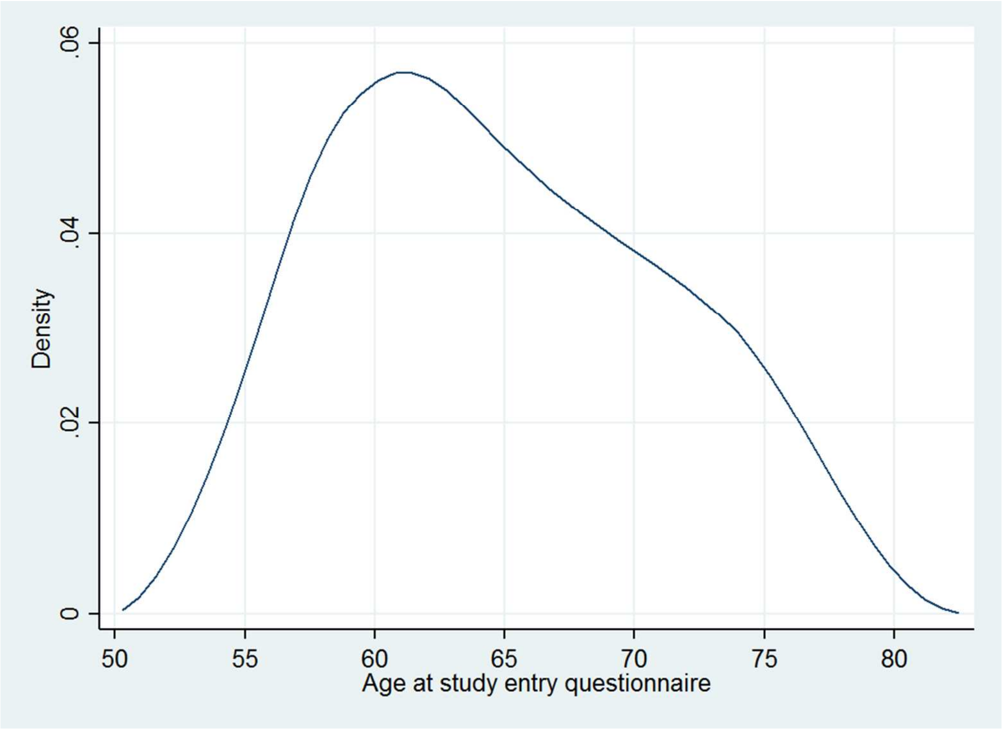

Supplementary Figure S1. Kernel density plot of age at study entry questionnaire.
370x269mm (72 x 72 DPI)

For peer review only

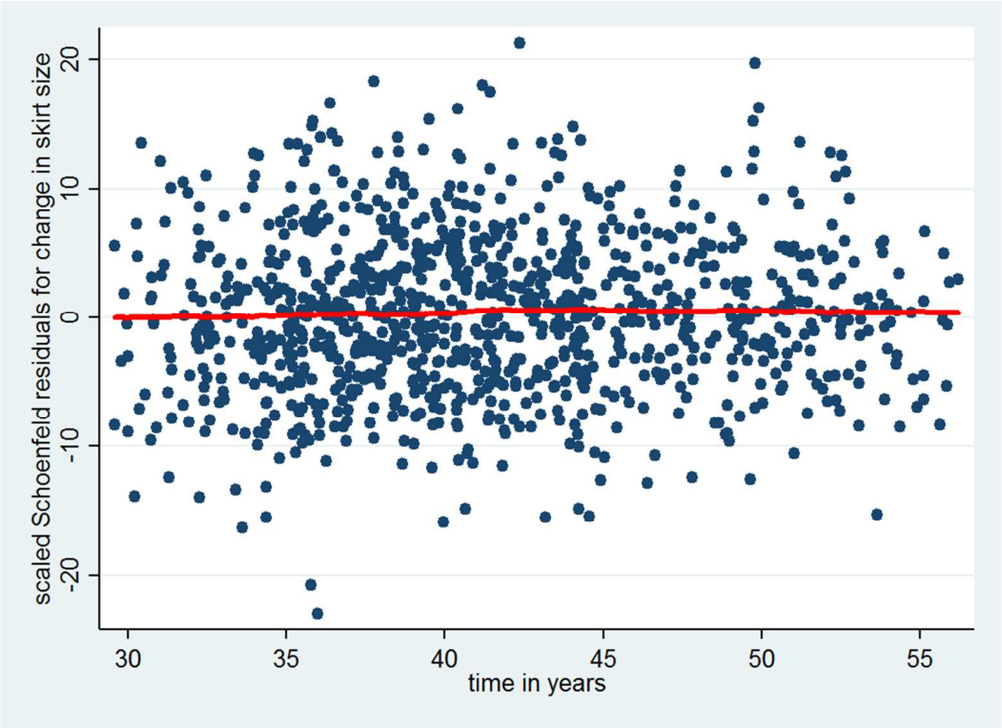

Supplementary Figure S2. Plot of the scaled Schoenfeld residuals for change of skirt size (CSS) with smoother.
370x269mm (72 x 72 DPI)

For peer review only

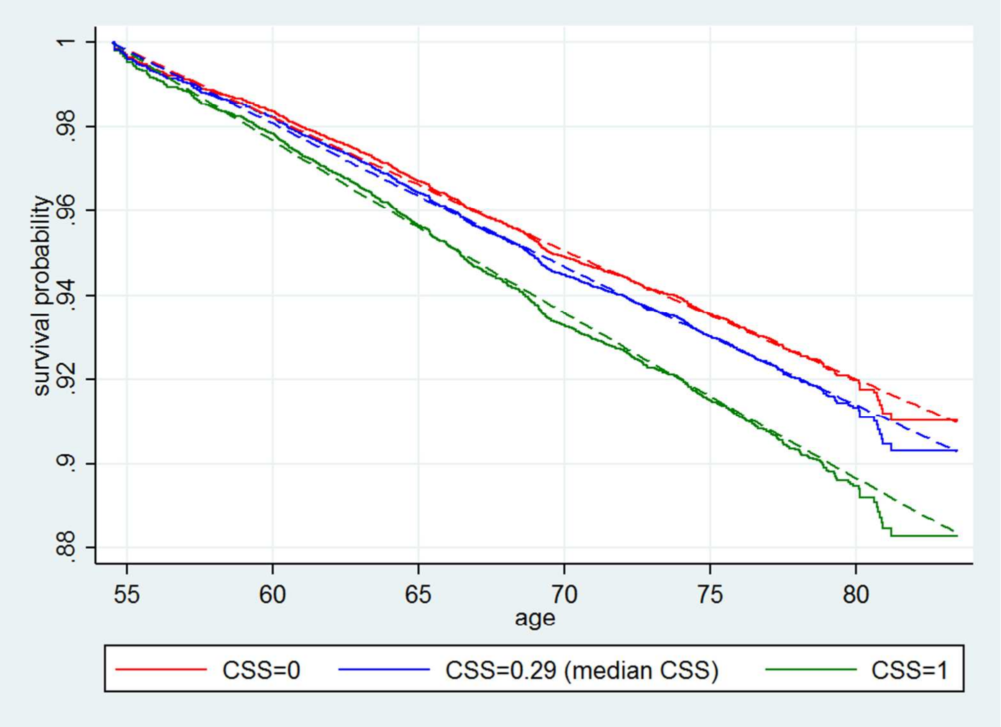

Supplementary Figure S3. Estimated survival functions for 3 values of change of skirt SS (CSS) (CSS=0, CSS=1, CSS=0.287 (median CSS)) with other covariate values set to their mean value. Solid lines represent estimated curves based on the Cox model, dashed lines represent estimated curves based on the exponential model.

370x269mm (72 x 72 DPI)

STROBE 2007 (v4) Statement—Checklist of items that should be included in reports of *cohort studies*

Section/Topic	Item #	Recommendation	Reported on page #
**Title and abstract**	1	(*a*) Indicate the study's design with a commonly used term in the title or the abstract	1-3
		(*b*) Provide in the abstract an informative and balanced summary of what was done and what was found	2-3
**Introduction**			
Background/rationale	2	Explain the scientific background and rationale for the investigation being reported	5-6
Objectives	3	State specific objectives, including any prespecified hypotheses	6
**Methods**			
Study design	4	Present key elements of study design early in the paper	7-8
Setting	5	Describe the setting, locations, and relevant dates, including periods of recruitment, exposure, follow-up, and data collection	7-8
Participants	6	(*a*) Give the eligibility criteria, and the sources and methods of selection of participants. Describe methods of follow-up	7-8
		(*b*) For matched studies, give matching criteria and number of exposed and unexposed	N/A
Variables	7	Clearly define all outcomes, exposures, predictors, potential confounders, and effect modifiers. Give diagnostic criteria, if applicable	7-9
Data sources/ measurement	8*	For each variable of interest, give sources of data and details of methods of assessment (measurement). Describe comparability of assessment methods if there is more than one group	*7-9*
Bias	9	Describe any efforts to address potential sources of bias	11
Study size	10	Explain how the study size was arrived at	7-9
Quantitative variables	11	Explain how quantitative variables were handled in the analyses. If applicable, describe which groupings were chosen and why	9-10
Statistical methods	12	(*a*) Describe all statistical methods, including those used to control for confounding	9
		(*b*) Describe any methods used to examine subgroups and interactions	N/A
		(*c*) Explain how missing data were addressed	10-11
		(*d*) If applicable, explain how loss to follow-up was addressed	8 and 10-11
		(*e*) Describe any sensitivity analyses	10-11
**Results**			

For peer review only

Participants	13*	(a) Report numbers of individuals at each stage of study—eg numbers potentially eligible, examined for eligibility, confirmed eligible, included in the study, completing follow-up, and analysed	12
		(b) Give reasons for non-participation at each stage	12
		(c) Consider use of a flow diagram	Not provided but described in the manuscript as stated above
Descriptive data	14*	(a) Give characteristics of study participants (eg demographic, clinical, social) and information on exposures and potential confounders	12
		(b) Indicate number of participants with missing data for each variable of interest	12-13 and 15
		(c) Summarise follow-up time (eg, average and total amount)	12
Outcome data	15*	Report numbers of outcome events or summary measures over time	12-13 and 15
Main results	16	(*a*) Give unadjusted estimates and, if applicable, confounder-adjusted estimates and their precision (eg, 95% confidence interval). Make clear which confounders were adjusted for and why they were included	13-14
		(*b*) Report category boundaries when continuous variables were categorized	N/A
		(*c*) If relevant, consider translating estimates of relative risk into absolute risk for a meaningful time period	15
Other analyses	17	Report other analyses done—eg analyses of subgroups and interactions, and sensitivity analyses	15
**Discussion**			
Key results	18	Summarise key results with reference to study objectives	19
**Limitations**			
Interpretation	20	Give a cautious overall interpretation of results considering objectives, limitations, multiplicity of analyses, results from similar studies, and other relevant evidence	16
Generalisability	21	Discuss the generalisability (external validity) of the study results	17-18
**Other information**			
Funding	22	Give the source of funding and the role of the funders for the present study and, if applicable, for the original study on which the present article is based	19

*Give information separately for cases and controls in case-control studies and, if applicable, for exposed and unexposed groups in cohort and cross-sectional studies.

For peer review only

Note: An Explanation and Elaboration article discusses each checklist item and gives methodological background and published examples of transparent reporting. The STROBE checklist is best used in conjunction with this article (freely available on the Web sites of PLoS Medicine at http://www.plosmedicine.org/, Annals of Internal Medicine at http://www.annals.org/, and Epidemiology at http://www.epidem.com/). Information on the STROBE Initiative is available at www.strobe-statement.org.

For peer review only
